# The phylogenomics of CRISPR-Cas system and revelation of its features in *Salmonella*

**DOI:** 10.1038/s41598-020-77890-6

**Published:** 2020-12-03

**Authors:** Simran Krishnakant Kushwaha, Narra Lakshmi Sai Bhavesh, Bahaa Abdella, Chandrajit Lahiri, Sandhya Amol Marathe

**Affiliations:** 1 Department of Biological Sciences, Birla Institute of Technology and Science (BITS), Pilani, Rajasthan India; 2 Department of Biological Sciences, Sunway University, Petaling Jaya, Selangor Malaysia; 3 Faculty of Aquatic and Fisheries Sciences, Kafrelsheikh University, Kafrelsheikh, Egypt

**Keywords:** Computational biology and bioinformatics, Evolution, Microbiology

## Abstract

*Salmonellae* display intricate evolutionary patterns comprising over 2500 serovars having diverse pathogenic profiles. The acquisition and/or exchange of various virulence factors influences the evolutionary framework. To gain insights into evolution of *Salmonella* in association with the CRISPR-Cas genes we performed phylogenetic surveillance across strains of 22 *Salmonella* serovars. The strains differed in their CRISPR1-leader and *cas* operon features assorting into two main clades, CRISPR1-STY/*cas*-STY and CRISPR1-STM/*cas*-STM, comprising majorly typhoidal and non-typhoidal *Salmonella* serovars respectively. Serovars of these two clades displayed better relatedness, concerning CRISPR1-leader and *cas* operon, across genera than between themselves. This signifies the acquisition of CRISPR1/Cas region could be through a horizontal gene transfer event owing to the presence of mobile genetic elements flanking CRISPR1 array. Comparison of CRISPR and *cas* phenograms with that of multilocus sequence typing (MLST) suggests differential evolution of CRISPR/Cas system. As opposed to broad-host-range, the host-specific serovars harbor fewer spacers. Mapping of protospacer sources suggested a partial correlation of spacer content with habitat diversity of the serovars. Some serovars like serovar Enteritidis and Typhimurium that inhabit similar environment/infect similar hosts hardly shared their protospacer sources.

## Introduction

Genus *Salmonella* is classified into two species, *Salmonella enterica (S. enterica)* and *S. bongori*. *S. enterica* evolved into six subspecies (subsp.) namely, *enterica*, *salamae*, *arizonae*, *diarizonae*, *houtenae* and *indica*^1^. The host-range for serovars of *S. enterica* subsp. *enterica* vary from broad-host-range to host-adapted and host-restricted^2^ pertinent to within-host evolution^3^. Before divergence, *S. bongori* and *S. enterica* acquired *Salmonella* pathogenicity island 1 (SPI-1)^4^ and later *S. enterica* laterally acquired SPI-2 thereby, enhancing its virulence potential^4^. As per the adopt-adapt model of bacterial speciation^5^, the adopted lateral gene(s) divert the evolutionary path promoting bacterial adaptation and consequently increasing its fitness^6^. Over time, both species horizontally acquired multiple virulence factors progressively enhancing their pathogenicity^3^.

Clustered Regularly Interspaced Short Palindromic Repeats (CRISPR) and a set of CRISPR-associated (*cas*) genes are suggested to be acquired by horizontal gene transfer (HGT) event^7,8^. The Cas1 and Cas2 proteins are essential for spacer acquisition from invading mobile genetic elements (MGE)^1^ while all Cas proteins participate in primed adaptation to update the invaders’ memory^9^. The newly acquired spacers are added at the leader proximal end of the CRISPR array^1^. Cas proteins work in conjunction with the CRISPR-RNA to carry out the interference step^2^. CRISPR-Cas system has been related to the bacterial virulence potential^10–13^. The number of CRISPR array are negatively correlated with pathogenic potential of *Escherichia coli* where, the reduction in CRISPR activity is proposed to promote HGT favouring its evolution^14^. Conversely, some reports demonstrate a positive correlation between the CRISPR and pathogenicity owing to virulence genes regulation^10,13,15^. In *S. enterica* subsp. *enterica* serovar Enteritidis, Cas3 modulates biofilm formation and virulence by regulating quorum sensing genes^13^. Further, in *Salmonella* and *E. coli*, 53% of CRISPR protospacers traced to chromosomes^8^ suggesting a potential role of the CRISPR-Cas system in endogenous gene regulation^16^ and possibly pathogenesis^13^.

*S. enterica* possesses type I–E CRISPR system comprising a *cas* operon and two CRISPR arrays, CRISPR1 and CRISPR2^17^, separated by ~ 16 kb^18^. The *cas* operon present in proximity to the CRISPR1 array^19^ contains 8 *cas* genes. Two distinct *cas* gene profiles has been observed with reported incongruence between the *cas* and whole genome phylogeny^20^. Similar nonconformity is noted for CRISPR array^21^. Contrarily, a phylogenetic congruence of the CRISPR loci and whole genome was obtained for strains of *S. enterica* subsp. *enterica* serovar Gallinarum biovar Pullorum^22^. Fricke et al*.* observed partial correlation between the CRISPR arrays and phylogeny of *S. enterica* isolates^23^. Studies on the phylogeny of CRISPR-Cas system have been done in other bacteria as well suggesting its role in shaping the accessory genome^24^. To test the association of CRISPR-Cas system with the serovar host/habitat diversity, we studied the evolutionary pattern of CRISPR-Cas system across strains of *Salmonella*. A graphic map of the spacers for 133 strains across 22 serovars belonging to two species of *Salmonella* provided a comprehensive view of its structural composition and configuration. The strains assorted into two groups with respect to the CRISPR1-leader and *cas* operon features. This divergence was analyzed in comparison to multi-locus sequence typing (MLST) based on the seven housekeeping genes. Spacer versatility was assessed with respect to protospacer source.

## Results

### Diversity of the CRISPR arrays in *Salmonella*

We extracted all possible CRISPR1 and CRISPR2 arrays in correct orientation for 133 *Salmonella* strains (Table S1, supplementary methodology). *S. bongori* and *S. enterica* subsp. *enterica* contained both CRISPR arrays while subsp. *arizonae* and *diarizonae,* had only one array. One, out of the six examined strains of subspecies *arizonae* had an intact CRISPR array.

**Figure 1:**
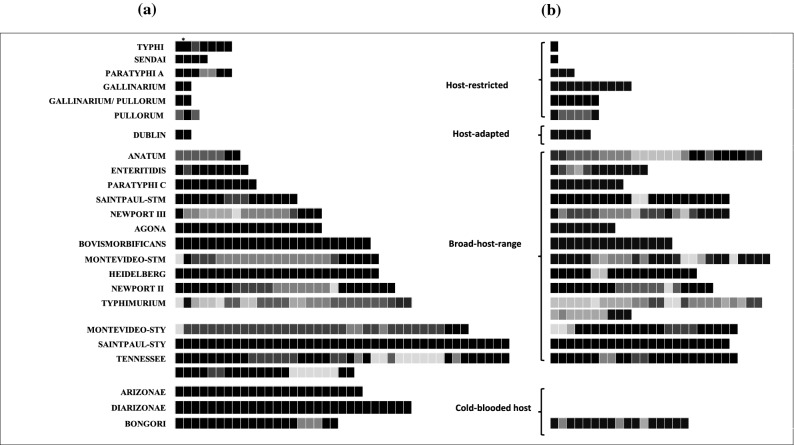
Graphic map of spacer conservation in CRISPR1 (**a**) and CRISPR2 (**b**) array for *Salmonella* serovars. The shades of grey represent the conservation percentage of a given spacer in all the strains of the respective serovar where, the darker box indicate the presence of spacer in most of the strains (black: 100%) while, the lighter box indicate the presence of spacer in a few strains. *Indicates merging of two spacers in a few strains of serovar Typhi.

We mapped the spacer sequences (Fig. S1) of all strains, illustrating the blueprint of spacer conservation among the strains within and across the serovars. The acquisition of spacers is in a precise fashion with conservation of spacer arrangement for a specific serovar. However, a few spacers are absent from CRISPR array(s) of some strains. The spacers of serovars Enteritidis, Heidelberg, and Typhi are highly conserved among their respective strains, whereas the serovars Typhimurium, Newport, Anatum, Montevideo, and Tennessee had significant variability in the spacer composition. (Figs. 1 and S1). Among all strains, we identified 440 and 330 unique spacers within the 2221 and 2211 spacers of CRISPR1 and CRISPR2 arrays, respectively. The average abundance of spacers for CRISPR1 and CRISPR2 is 15.3 and 12.6, respectively (Table S2). CRISPR1 array of serovar Tennessee str. ATCC 10722 (63 spacers) and CRISPR2 array of serovar Typhimurium str. USDA-ARS-USMARC-1880 (35 spacers) are the largest (Fig. S1). CRISRP1 array of serovar Anatum, Dublin, Gallinarum, Pullorum and, Gallinarum/Pullorum (two spacers), and CRISPR2 array of serovars Sendai and Typhi (one spacer) are the shortest (Fig. S1). We observed duplication and triplication of spacer(s) in some serovars (Fig. S1a,b).

Strikingly, the analysis of the CRISPR arrays in serovars Montevideo and Saintpaul separated the respective strains into two groups each with two distinct sets of unique and conserved spacers (Table S1). For serovar Montevideo, the two groups comprised eight (later defined as Montevideo-STM) and nine strains (later defined as Montevideo-STY) (Fig. S1). However, CRISPR arrays of all the analyzed strains of serovar Saintpaul (that we define as Saintpaul-STM), except strain SARA26 (an outlier, that we define as Saintpaul-STY), had similar spacer composition. These results suggest the serovars Montevideo and Saintpaul could be polyphyletic with respect to CRISPR1 loci, similar to that reported for serovar Newport^25^. Notably, the broad-host-range serovars have multiple spacers, while the host-specific serovars have few spacers (Fig. 1 and Tables S1, S2).

The direct repeat (DR) sequence is conserved within respective array across all the serovars except for the presence of few SNPs (Figs. S2a, c, e). The last DR is degenerate^26^ (Figs. S2b, d, f) with significant variation near the 3′ end.

### Phylogeny and classification of the CRISPR loci

**Figure 2:**
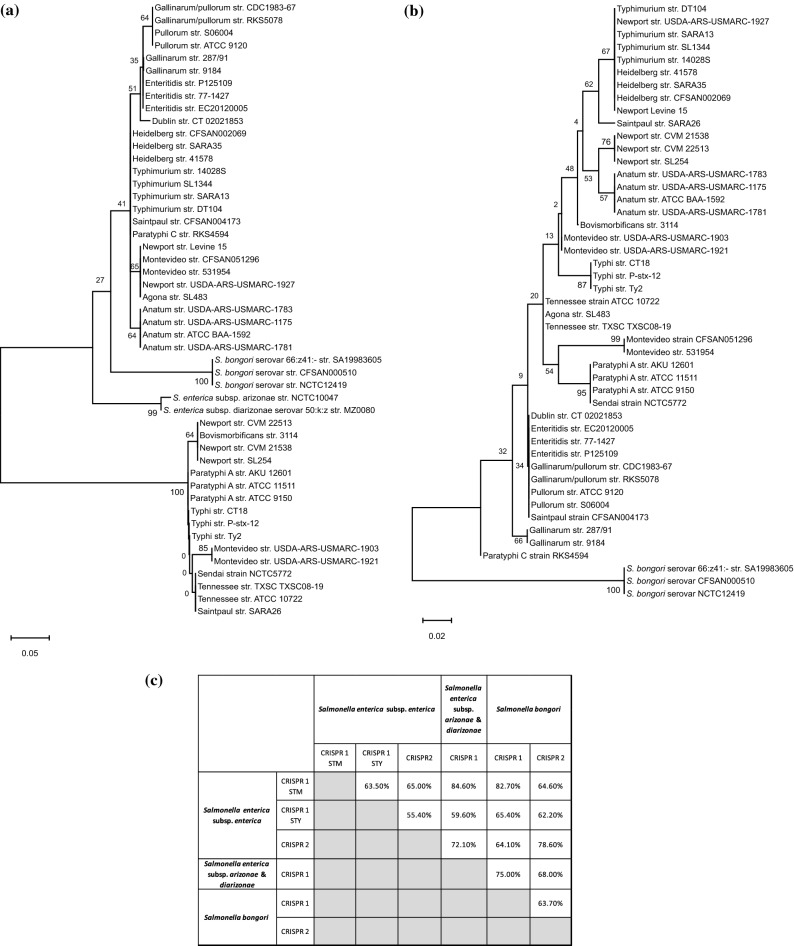
The phylogeny and conservation of CRISPR-leaders, CRISPR1 (**a**), and CRISPR2 (**b**) across *Salmonella* serovars. The CRISPR-leader sequences were aligned using MUSCLE and the phylogenetic tree was constructed using ML. **(c)** A matrix depicting the inter-species and inter-subspecies conservation of the leader sequence of both the CRISPR arrays. The values represent the percent nucleotide identity with respect to the entire query cover. The reference strains are *S. enterica* subsp. *enterica* serovar Typhimurium str.14028S, Typhi str. CT18, *S. enterica* subsp. *arizonae* str. NCTC10047*, S. enterica* subsp. *diarizonae* str. MZ0080 and *S. bongori* str. SA19983605.

**Figure 3:**
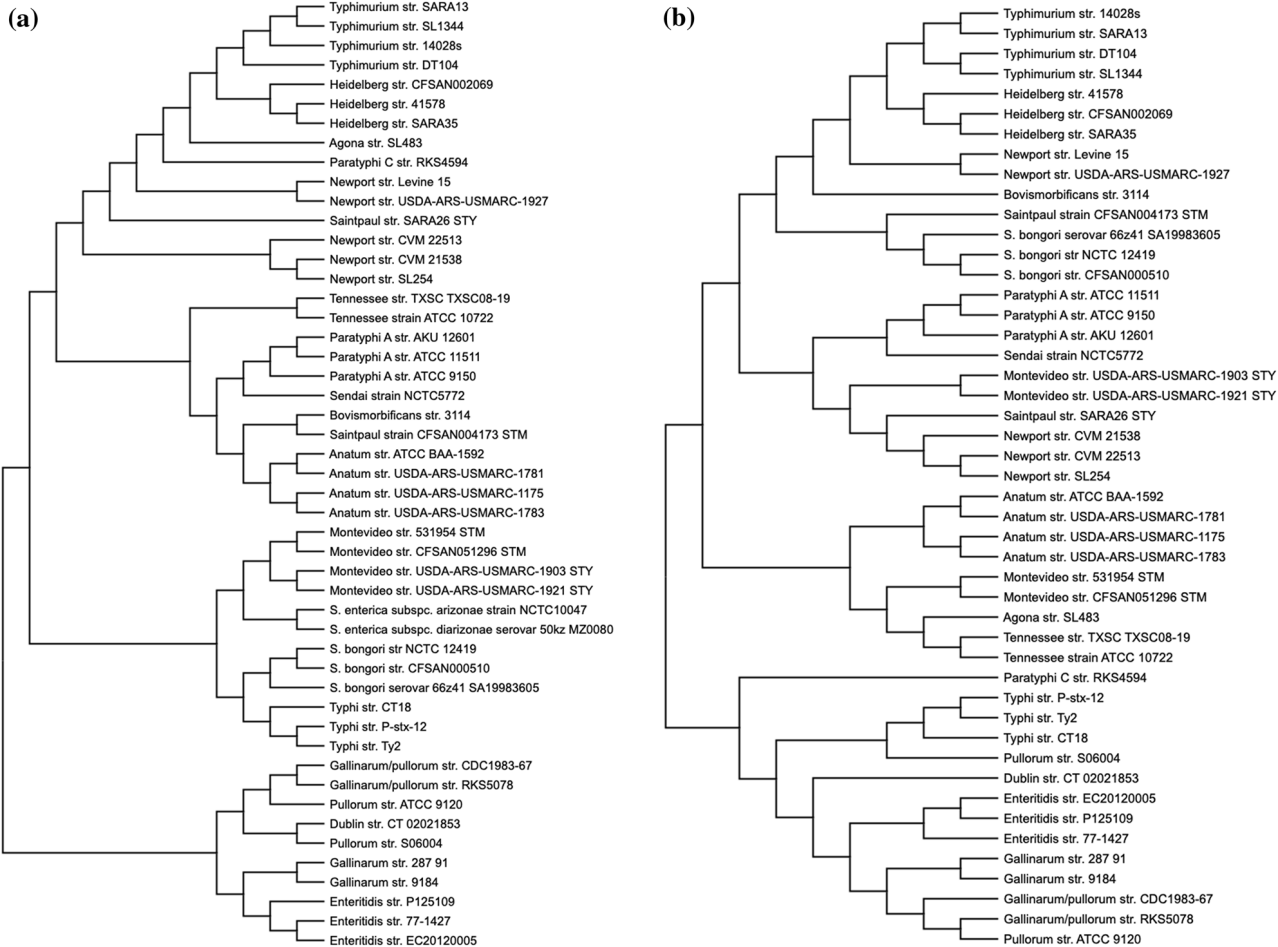
The phylogeny of CRISPR spacers. Neighbour-joining tree was constructed based on distance matrix analysis of the spacer content of the CRISPR1 (**a**) and CRISPR2 (**b**) array. Serovars Heidelberg, Newport III and Typhimurium; Paratyphi A and Sendai; and Dublin, Enteritidis, Gallinarum, Pullorum and Gallinarum/Pullorum club together in both trees and are named as HNT, PS and DEGP clade.

Further analysis was performed on 49 shortlisted strains representing different species, subspecies and serovars with varied host-range (Table S1). Minimum number of strains of each serovar were chosen to represent almost all combinations of the spacers. To understand the evolutionary pattern of *Salmonella* serovars concerning the CRISPR loci, we generated phylogenetic trees for the leader sequences (Fig. 2) and spacers (Fig. 3).

#### Evolutionary studies of the CRISPR leader

For the leader phenogram, the topology has been observed in most of the clades and sub-clades, as evidenced by their high level of confidence from either the bootstrap values or the aLRT (approximate likelihood ratio test) scores. The CRISPR1-leader tree had two distinct clades majorly comprising typhoidal and non typhoidal *Salmonella* serovars^20^ (Fig. 2 and S3a). Thus, we classified the corresponding CRISPR loci as CRISPR1-STM and CRISPR1-STY, respectively. The strains of serovars Saintpaul and Montevideo harboring these loci were accordingly defined as Saintpaul-STM/Montevideo-STM, and Saintpaul-STY/ Montevideo-STY. The CRISPR1-STM clade included strains that are host-adapted, host-restricted or have broad-host-range (Fig. 2a and Table S1–S2)^27^. The CRISPR1-STY/*cas*-STY clade also contain the serovars Montevideo, Newport-II, Tennessee, Bovismorbificans and Saintpaul having broad-host-range^28,29^ and association with outbreaks of human salmonellosis^30–32^.

In CRISPR2-leader phenogram (Fig. 2b and S3b), *S. bongori* emerged as an outgroup for the entire tree, and serovar Paratyphi-C seems to have evolved distinctly from other serovars of *S. enterica* subsp. *enterica*. The topology and sub-lineages were very distinct from that of the CRISPR1-leader tree with intermixing of serovars of the two distinct clades. For example, serovar Saintpaul-STY grouped with serovars Typhimurium, Newport-III and Heidelberg whereas, Sendai and Paratyphi-A grouped with Montevideo-STM while Newport-II clubbed with Anatum. This suggests different evolutionary trajectories of both the CRISPR loci.

#### Categorization of the leader sequence in the light of CRISPR leader phylogeny

The leader sequence analysis suggests serovars of *S. enterica* subsp. *enterica* have two distinct types of CRISPR1-leaders (Fig. 2a and Fig. S3a), justifying their divergence in two clades. One of the leader sequences is identical to that of Newport-II^18^ and is present in all the serovars of CRISPR1-STY clade. Serovars Enteritidis, Gallinarum, Pullorum and Gallinarum/Pullorum have < 98% leader identity, thus, cluster in the CRISPR1-leader tree (Fig. 2a). On similar grounds, other serovars cluster or separate from each other. The CRISPR1-leader of *S. bongori,* and *S. enterica* subsp. *arizonae* and subsp. *diarizonae* maximally matched with that of CRISPR1-STM (Fig. 2a and Fig. S3a) and hence grouped in the CRISPR1-STM clade.

The CRISPR2-leader sequence is highly conserved (with a few SNPs) among all the serovars of *S. enterica* subsp. *enterica* (Fig. 2b and Fig. S3b) justifying their segregation from *S. bongori.* The variations due to SNPs explain the serovar clustering in the CRISPR2-leader tree. For instance, the leaders of serovars Paratyphi-A and Typhi having 94% sequence similarity segregated into separate clades while the serovars Paratyphi-A and Sendai clubbed together with 100% similarity.

#### Evolutionary study of CRISPR arrays

The phylogeny of CRISPR arrays was studied with respect to the spacer content. Only ~ 8.6–9.6% of unique spacers (37/440: CRISPR1 and 32/330: CRISPR2) were shared by two or more serovars (Fig. S1c-d). Thus, the spacer trees were constructed based on presence-absence matrix. In both the CRISPR1- and CRISPR2- spacer trees, serovars Enteritidis, Dublin, Gallinarum, Gallinarum/Pullorum and Pullorum formed one clade (clade-DEGP) while the other serovars formed the second (Fig. 3). In CRISPR2-spacer tree, serovar Typhi and Paratyphi-C grouped with clade-DEGP sharing anchor spacer with these serovars (Fig S1d). The second clade had three distinct subclades with serovar composition of two (named as HNT and PS, Fig. 3 and Fig. S5) was partially constant: serovars Heidelberg, Newport-III and Typhimurium in clade-HNT and serovars Paratyphi-A and Sendai in clade-PS. Serovars within these clade (clade-DEGP) and sub-clade (clade-HNT & clade-PS) share many spacers of both the arrays (Fig. S1c,d). However, the other serovars show spacer match with random serovars (Figs. S1c,d and S5) and hence cluster differently in both the spacer trees. *S. enterica* subsp. *arizonae* and *diarizonae* (both possessing only CRISPR1 array) and *S. bongori* associated with poikilotherms do not form a separate clade but intermix with the serovars of *S. enterica* infecting endotherms.

In CRISPR1-spacer tree, serovars Agona, Newport-II, Paratyphi-C and Saintpaul-STY grouped with clade-HNT as they share anchor spacer with these serovars (Fig S1c). Serovars Anatum, Bovismorbificans, Saintpaul-STM and Tennessee clubbed with clade-PS, while serovars Typhi and Montevideo groupped with the species/subspecies that are associated with poikilotherms. In CRISPR2-spacer tree, *S. bongori*, serovar Bovismorbificans and Saintpaul-STM grouped with clade-HNT while serovars Newport-II, Saintpaul-STY and Montevideo-STY with clade-PS as they share anchor spacer with Paratyphi-A (Fig. S1d). Serovars Agona, Montevideo-STM, Anatum and Tennessee formed a separate sub-clade. Serovars Anatum and Tennessee grouped in both the trees but had different relationship with other clades.

#### MLST phenogram and its association with the CRISPR array

**Figure 4:**
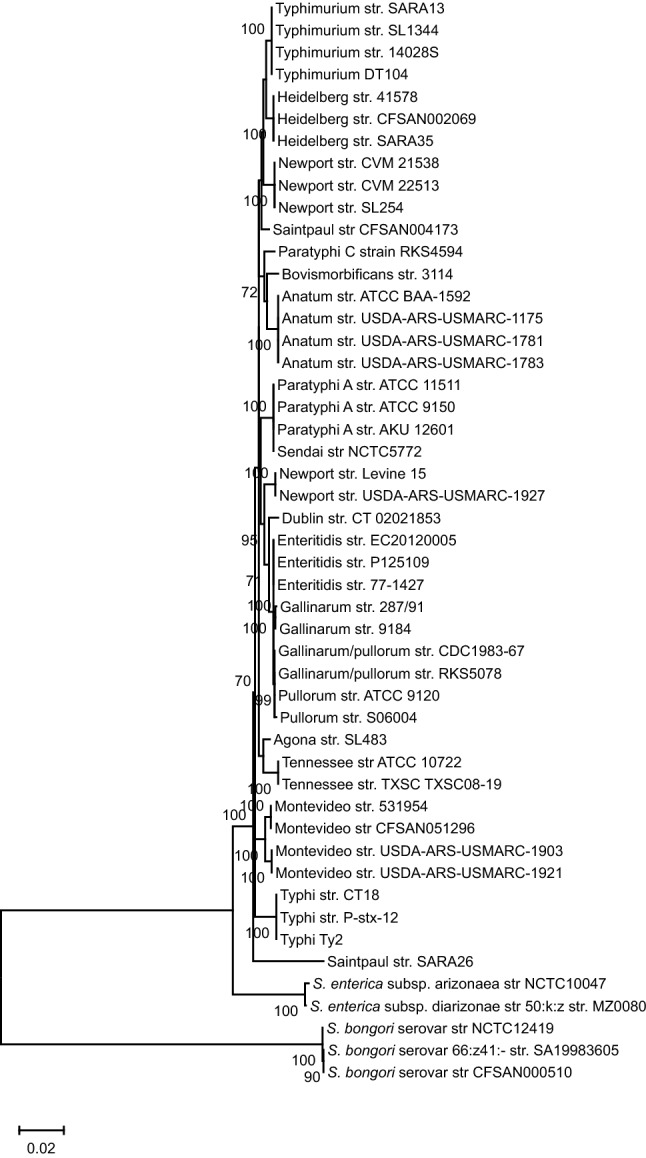
The MLST phylogeny. The phylogenetic tree was constructed using the concatenated sequences of seven housekeeping genes-*purE, hemD, aroC, dnaN, hisD, thrA*, and *sucA*. The sequences were aligned using MUSCLE and phylogenetic tree was constructed by ML.

MLST is considered as a robust and widely accepted phylogenetic reflection of the species taxonomy^33^. Hence, we generated a reference MLST tree for the shortlisted strains (Table S1) using concatenated allelic data of seven housekeeping genes (Fig. 4). *S. bongori* separated out as a distinct clade from other *S. enterica* serovars. All other serovars formed lineages within a serovar-specific cluster depicting to have evolved together as an individual taxon except serovar Saintpaul and Newport. Serovar Saintpaul str. SARA26 separated from all serovars of subspecies *enterica* and str. CFSAN004173 clustered with Typhimurium/Heidelberg/Newport-II group. In this light, serovar Saintpaul turns out to be polyphyletic like serovar Newport^34^. Serovar Paratyphi-A is closer to serovar Typhimurium with 98.8% similarity in the seven genes than to serovar Typhi (98.6% similarity). The CRISPR and MLST phenograms are discordant with respect to their topology thereby signifying differential evolutionary path of the CRISPR loci (possibly due to a plausible acquisition of CRISPR loci through HGT) than that of the housekeeping genes. Serovars Montevideo-STM and Montevideo-STY possess the same housekeeping genes but differ in CRISPR arrays segregating in two groups in CRISPR phenograms.

### Phylogeny and classification of the *cas* operon

#### Diversification of *cas* operon and its association with the CRISPR1 array

Two distinct *cas* gene arrangements were obtained for the strains comprising CRISPR1-STY and CRISPR1-STM clades. Thus, the *cas* operon of the respective categories were denoted as *cas-*STY and *cas-*STM. For *cas-*STY, the *cas3* gene is present as a complement and is singled out from the other *cas* genes by a gap of 357 nucleotides (561 for serovar Montevideo-STY) (Fig. S6). For *cas-*STM, the *cas* genes are contiguous but the *cas3* gene of serovar Montevideo-STM and *S. enterica* subsp. *arizonae* is degenerate having a premature stop codon. Moreover, we noticed structural heterogeneity within the *cas-*STM operon across CRISPR1-STM strains, with respect to its position in both the CRISPR loci and the *cas* gene composition (Fig. S6). The *cas* operon of *S. bongori, S. enterica* subsp. *enterica*, subsp. *arizonae* and subsp. *diarizonae* were termed as *cas*-STM.B, *cas*-STM.E, *cas*-STM.A, and *cas*-STM.D, respectively.

#### Evolutionary studies and conservation of *cas* operon in *Salmonella*

**Figure 5:**
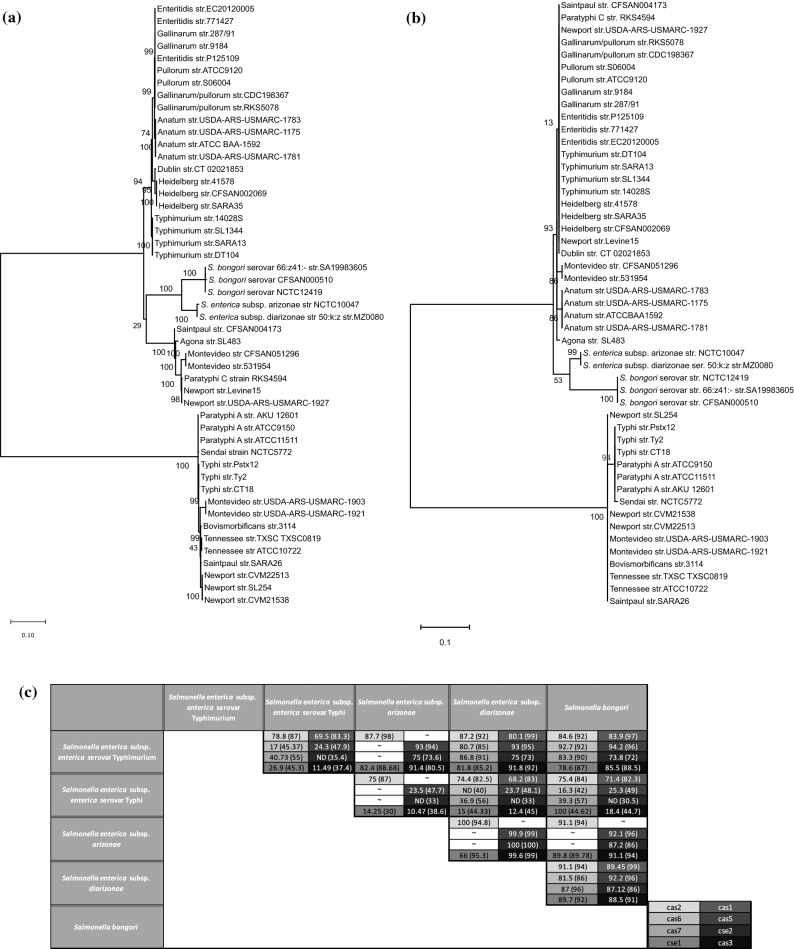
The phylogeny and conservation of *cas* genes. (**a**,**b**) Phylogeny of *cas* genes across *Salmonella* serovars for entire *cas* operon (**a**) and the *cas3* gene (**b**). The sequences were aligned using MUSCLE, and the phylogenetic trees were constructed by ML. (**c**) Conservation of all the individual *cas* gene and Cas protein sequences. The amino acid percent conservation is depicted in parenthesis. The term ‘ND’ represents no nucleotide sequence similarity based on the default parameter of the tool Nucleotide-BLAST. The bootstrap values are indicated at each node. The reference strains used were *S. enterica* subsp. *enterica* serovar Typhimurium str.14028S, Typhi str. CT18, *S. enterica* subsp. *arizonae* str. NCTC10047, *S. enterica* subsp. *diarizonae* str. MZ0080 and *S. bongor*i str. SA19983605.

The *cas* operon’s heterogeneity was further assessed through phylogenetic analysis of the *cas3* gene and the entire *cas* operon (Fig. 5 and supplementary Fig. S8). Two clades and the clustering of serovars obtained in both the phenograms is far more analogous with the CRISPR1- leader phenogram. To gain insights into the serovar clustering in *cas* genes, we performed a detailed comparative analysis of *cas* operon. The analysis of all *cas* genes considered in concatenation revealed the highest nucleotide similarity (99%) between subspecies *arizonae* and *diarizonae* and lowest (28.6%) between the *cas-*STM and *cas-*STY groups (Fig. S7). Between the latter groups, *cas1* shares the highest similarity (74.4–78.8% nucleotide and 82.5–87% amino acid match) while *cse2* shares the lowest similarity (no significant nucleotide match and 35% amino acid identity) (Fig. 5). The Cas3 nuclease of *cas*-STM showed poor nucleotide (10.47–18.4%) and amino acid (37.4–45%) match with that of *cas*-STY category. However, the functionally important domains- HD domain (~ 48%), helicase C-terminal domain (~ 77%), and the DEAD-box (~ 81%) (Fig. S9a) were similar. The *cse1* gene, was quite distinct between the *cas-*STM and *cas-*STY categories. The functionally important residues of Cse1 from *E. coli* include Gly (157), glycine-loop residues (159–161), Lys (268), Asn (353), Glu (354) and Ala (355) required for the recognition of PAM sequences^35^ and lysine residues (289–290) for recruiting Cas3 protein^35^. Most of these residues are conserved across the *cas*-STM and *cas*-STY categories (Fig. S9b) indicating that even though the Cse1 and Cas3 differs significantly between these serovars, their functionality remains conserved.

### Inter-genus analysis of the CRISPR-Cas system

**Figure 6:**
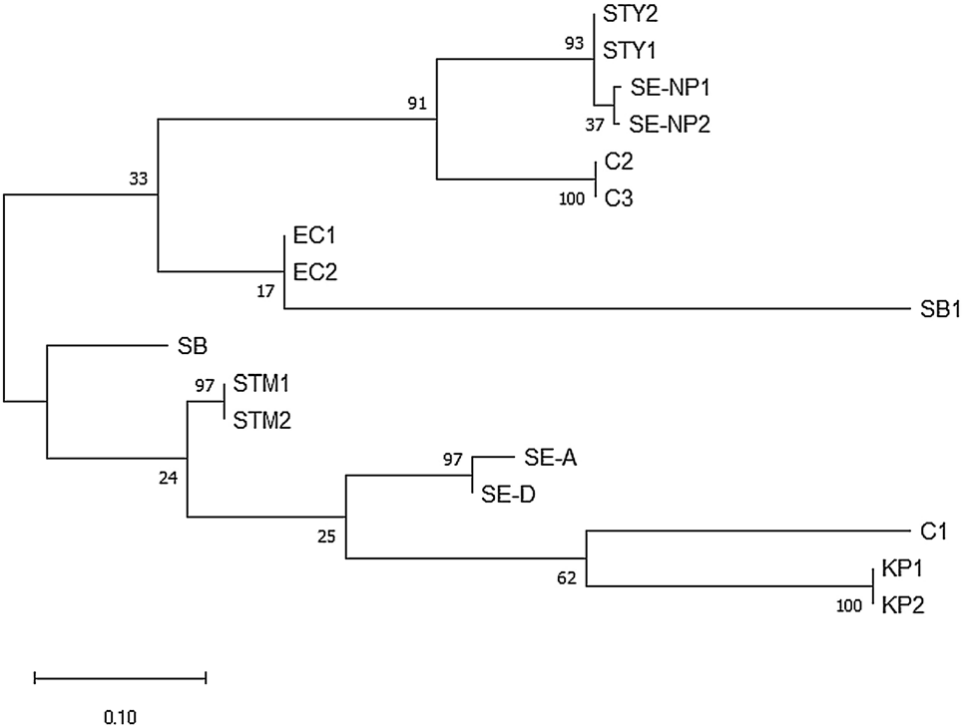
The Phylogeny of the CRISPR1-leader sequence of 17 strains of species of *Enterobacteriaceae* family. The CRISPR1-leader sequences were aligned using MUSCLE, and the phylogenetic tree was constructed by ML. The bootstrap values are indicated at each node. KP—*Klebsiella pneumoniae*, C—*Citrobacter*, SE-A—*S. enterica* subsps. *arizonae*, SE-D—*S. enterica* subsps. *diarizonae*, STM—*S. enterica* subsp. *enterica* serovar Typhimurium, STY—*S. enterica* subsp. *enterica* serovar Typhi, SE-NP—*S. enterica* subsp. *enterica* serovar Newport, SB—*S. bongori*, SB1—*Shigella boydii* and EC—*Escherichia coli*.

The evolutionary history of the CRISPR and *cas* loci across all species of *Enterobacteriaceae* family was studied through comparative sequence analysis and phylogenetics. Through local alignment, we found that the CRISPR1-leader of *Salmonella* showed substantial match across strains of *Escherichia, Citrobacter, Shigella,* and *Klebsiella* (Table S4), all occurring in similar habitats and possessing type I-E CRISPR-Cas system^36,37^. However, the CRISPR2-leader matched only with *Klebsiella*. Thus, we constructed a CRISPR1-leader phenogram with representative strains belonging to these genera (Table S3), and some strains of CRISPR1-STM and CRISPR1-STY clades. The phylogenetic tree diverged into two main clades (Fig. 6) similar to the CRISPR1-leader tree of *Salmonella* with the same signature serovars. The strains of CRISPR1-STY category grouped with *Escherichia, Shigella* and some strains of *Citrobacter* (Fig. 6) while the strains of CRISPR1-STM clustered with *Klebsiella,* and a strain of *Citrobacter* (Fig. 6)*.*

The *cas-*STM operon showed ~ 75% similarity with that of the species *Klebsiella pneumoniae* (str. TGH10)*, Citrobacter freundii* (sp. CFNIH3)*,* and *Shigella boydii* (str. ATCC 49812)*,* which is significantly higher than that with *cas*-STY (28.6%). On the contrary, the *cas-*STY operon displayed ~ 84% similarity with *Citrobacter freundii* (sp. CFNIH9) and *Citrobacter* (sp. 30_2)*.* Intriguingly, the *ca*s-STY showed only a 12% match with *E. coli*.

### CRISPR-Cas system is flanked with MGE

**Figure 7:**
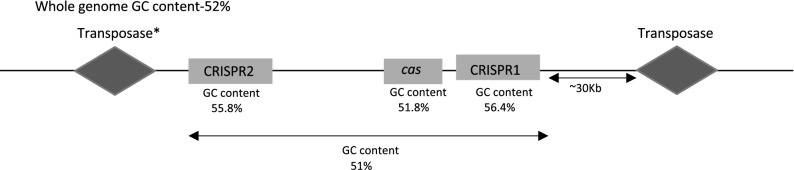
Generalised representation of the signature genes involved in horizontal gene transfer. All *Salmonella* serovars except serovars Bovismorbificans and Gallinarum/Pullorum contain the transposase gene upstream of CRISPR1 loci. *—transposase upstream of CRISPR2 is present only in serovars Typhi and Typhimurium.

To decipher the probable involvement of HGT, we screened the presence of the signature MGE namely, helicase, transposase, and integrase^7,38^ in the proximity of the CRISPR-Cas region of *Salmonella*. To this end, we also analyzed the GC content of this region in comparison to the whole genome. We found that 18 out of 20 serovars*,* (with representative strains of each considered) showed truncated/probable transposase at a position 30 kb upstream of the CRISPR1 loci (Fig. 7 and Table S5). The transposable elements are not uniformly found within ± 30 kb of any region in the genome (Table S6) suggesting CRISPR could have been possibly acquired via transposition. The GC content of the CRISPR arrays for most of the serovars was higher than the GC content of the whole genome except for a few serovar with smaller arrays which had lower GC content due to AT rich leader sequence (Fig. 7 and Table S5). A transposase gene was also present upstream of CRISPR2 array in serovars Paratyphi-A and Typhi. Moreover, a helicase gene was found to be present downstream of the CRISPR2 array in the serovars Typhi and Typhimurium.

### Mapping protospacer sources of CRISPR spacers

We mapped the protospacer sources (plasmids, phages and viruses) using CRISPRTarget tool^39^ and compared across serovars (Fig. S11 and Table S7). Common protospacer sources were observed majorly for the serovars sharing spacers with each other. For example, serovars Heidelberg and Typhimurium shared sufficiently high protospacer sources compared to other serovar pairs. Thus, even though the serovars inhabit/infect similar habitats/hosts e.g. serovar Enteritidis and Typhimurium they differ in their protospacer sources. Protospacers were not traced for a substantial proportion (~ 36% ± 14.8–15.6) of spacers (Table S7). No correlation was observed between number of spacers and protospacers especially for arrays with high spacer content (Fig. S12).

In serovar Typhimurium str. 14028s, 236 spacer- and Cascade-binding sites were identified using ChIP-seq of Cas5^40^. After mapping these sites on the complete genome of serovar Typhimurium str. 14028s (supplementary methodology) we found that some of these sites corresponds to virulence genes like *sseA, bcsA, iro, ent, sptP*, etc. (Table S8). This suggests a potential regulation of pathogenic traits by CRISPR-Cas system.

## Discussion

The evolutionary mechanisms in bacteria are highly complex with environmental factors intricately modulating the genome architecture and functionality. Further, HGT and recombination events significantly influence the evolutionary framework of the bacteria. Our study probes into the evolution of *Salmonella* with respect to CRISPR-Cas system that influences genome evolution^16^ and bacterial virulence^13^. We categorized the CRISPR-Cas system into two types, namely, CRISPR1-STM/*cas*-STM and CRISPR1-STY/*cas*-STY based on the phylogenetic segregation and differences in the CRISPR1-leader and *cas* genes features of the strains studied. Similar segregation pattern was observed with a large set of 128 strains (Fig. S10).

The CRISPR–Cas evolution is portrayed as complex having modular character hindering its forthright categorization based on the serovar host-range and geographical location. Both the serovars Newport-II and Newport-III, infect primates, reptiles and aves^41^ but still segregate into two separate clades in the CRISPR1-leader tree. Serovar Typhimurium strain SARA13 and Saintpaul SARA26 were isolated from the same geographic location, viz. France (GenBank database), but segregated into CRISPR1-STM and CRISPR1-STY clades, respectively. The conservation of array within strains of all the serovars, irrespective of the geographic location, suggests CRISPR acquisition to be a primeval event.

The chronicles of battles between the bacteria and the invading MGE are registered as spacers in the CRISPR arrays. The spacer conservation was weak across the serovars but significant within themselves except for those of serovars Montevideo, Newport and Saintpaul. However, spacer variability was observed within a few serovars like Typhimurium and Newport-III showing some variations in their CRISPR1-spacer composition (Fig. S1). Thus, the acquisition of the spacers could be a primitive event, with different selection pressures operating on different serovars to maintain the spacer composition. One elucidation is, the spacer composition of the system could potentially leverage protection against invading MGE^16^ or pathogenic potential possibly through endogenous gene regulation^10,13,42^ as implicated elsewhere^10,13,16,42^, thereby resulting in the spacers preservation. This polymorphism of spacers, across serotypes, finds utility in serotyping^43,44^.

The CRISPR1- and CRISPR2- spacer trees were distinct from each other. However, some serovars (clade-HNT, clade-PS, and clade-DEGP) were consistently grouped in all the CRISPR and *cas* trees implying a highly conserved CRISPR-Cas system within the serovar-group. For example, serovar Heidelberg have 66% of CRISPR1- and 100% of CRISPR2- spacers identical with those of the serovar Typhimurium. This may indicate a recent divergence of these serovars in the evolutionary timeline of *Salmonella*. Notably, some serovars like Bovismorbificans, Anatum, Saintpaul, Montevideo, and Typhi grouped differently in CRISPR-leader and -spacer phenograms. This indicates random spacer acquisition/loss or multiple HGT-events in these serovars. Further, spacer tree analyses suggest that the grouping and segregation of the serovars is independent of host-specificity and their habitat. For example, a primate specific serovar Typhi clubbed with bird/cattle-specific serovars. Moreover, the serovars with similar host-range or habitat largely have non-overlapping protospacer sources (comprising MGE, Fig. S11).

The anchor spacer gives an indirect correlation of the last common ancestor (LCA) for the array and is generally conserved for a particular serovar^18^. Many serovars of the clades in the spacer tree share the anchor spacer (Figs. 3 and S1c,d), thereby suggesting an LCA for the array in each clade. However, for some serovars other spacers, but not the anchor spacer, are shared. For instance, the serovar Gallinarum shares CRISPR1 spacers with Enteritidis but not the anchor spacer, implicating the loss of some common spacers including the anchor spacer. Serovar Bovismorbificans share five CRISPR1 spacers with serovar Saintpaul-STM, and anchor spacer with serovar Newport-II thereby indicating divergence from Newport-II and recombination with Saintpaul-STM.

The *cas* genes of the strains, in the *cas*-STM and *cas*-STY categories, are highly similar within each category but differ from the other, except for the *cas1* and *cas2* genes required for spacer acquisition^45^. However, the key residues of Cse1 and the functional domains of Cas3 are conserved indicating the conservation of their functionality. The strains, comprising *cas*-STM and *cas*-STY, are identical to CRISPR1-STM and CRISPR1-STY, respectively. This is empirical, as the CRISPR1 array and the *cas* operon are juxtaposed. Furthermore, the strains belonging to CRISPR1-STY/*cas-*STY category showed higher substitutions per sequence site (Fig. 5), implying the plasticity for new alterations.

The size of the spacer set for a given serovar is proportional to its host-range (Fig. 1). Ubiquitous serovars like Typhimurium, Newport-II, Tennessee, and Heidelberg have huge spacer set while host-specific/adapted serovars like Typhi, Sendai, Gallinarum, Dublin possess a few spacers. Considering the role of spacers in regulating endogenous genes^46^ and preventing invading MGE^16^ we put forward two possible hypotheses. The spacer versatility in broad-host-range serovars can be due to the exposure to a wide range of environments and/or it permits regulation of different genes. In both cases, the bacteria possibly gains advantage of adapting to multiple stress factors like attack by MGE and hostile host conditions. All the spacers of the host-specific serovars Gallinarum, Pullorum, and Gallinarum/Pullorum are present in serovar Enteritidis (a broad-host-range serovar) along with some additional spacers further testifying the hypotheses. The sources of protospacers (MGE) among these serovars are reasonably common (Fig. S11). Moreover, even though serovar Enteritidis^47^ is a broad-host-range serovar and share the habitats (e.g. mammalian gut) with that of serovar Typhimurium^47^ and Heidelberg^48^ they hardly have common protospacer source. Further, the binding of Cascade complex along with endogenous crRNA to > 100 chromosomal targets in *E.coli*^49^ and *S. enterica* subsp. *enterica* serovar Typhimurium^39^ indicate regulation of gene expression by CRISPR-Cas system. A further support to endogenous gene regulation is obtained through the results of Cui et al*.*^13^ showing regulation of virulence and biofilm genes by CRISPR-Cas system.

Among the host-specific/adapted serovars, the primate-specific serovars, namely, Typhi, Paratyphi-A, and Sendai, have a CRISPR1-STY/*cas*-STY system. The remaining four serovars are specific to poultry or cattle containing the CRISPR1-STM/*cas*-STM system. We propose that CRISPR1-STY/*cas*-STY system may provide some advantage to serovars of CRISPR1-STY clade. This would be either to prevent MGE invasion or regulate endogenous genes in primate (a restricted host for typhoidal serovars) gut. Nevertheless, the serovars do not have common protospacer source, possibly indicating some advantage in endogenous gene regulation. However, in-depth analyses and further research are warranted to understand any advantage of having a CRISPR1-STY/*cas*-STY system in these serovars.

The incongruence in CRISPR and *cas* trees with the MLST tree implies a plausible event of HGT. Similar incongruency with the CRISPR-Cas system of whole genome phylogeny is also reported elsewhere^20,21^. A truncated transposase, ~ 30 kb upstream of the CRISPR1 array and a high GC content of the CRISPR array possibly hints the occurrence of HGT event^50,51^. A further support is evidenced through the histone-like nucleoid-structuring protein (H-NS) mediated regulation of *cas* operon in *S. enterica* subsp. *enterica* serovar Typhi^52^. H-NS is associated with HGT, acting as a transcriptional silencer of horizontally acquired genes by binding to the AT rich DNA and blocking RNA polymerase^3^. One may possibly argue the regulation of CRISPR array by H-NS through its AT-rich leader as reported for *E. coli*^3,53^. Thus, H-NS could have originally silenced the CRISPR-Cas system and later evolved to regulate the functioning of *cas* operon and the CRISPR arrays. However, validation of such mechanism in other strains of *Salmonella* needs further accreditation.

*S. bongori*, *S. enterica* subsp. *arizonae* and subsp. *diarizonae,* cluster in ‘CRISPR1-STM’ and ‘*cas*-STM’ clades of CRISPR1 and the *cas* phenograms, thereby reflecting a higher similarity with CRISPR1-STM/*cas*-STM than with CRISPR1-STY/*cas*-STY (Figs. 2 and 5) Interestingly, the CRISPR1-STM/*cas*-STM and CRISPR1-STY/*cas*-STY sequences showed better relatedness with other genera of *Enterobacteriaceae* family namely, *Escherichia*, *Klebsiella, Shigella,* and *Citrobacter* than with each other (Fig. 6). More than 600 strains belonging to *Escherichia*, *Shigella,* and *Klebsiella* have the CRISPR/Cas system that matched with CRISPR1-STM/*cas*-STM (Table S4). Nevertheless, few strains of the *Enterobacteriaceae* family (*Klebsiella* & *Citrobacter*) contain both CRISPR1-STM and CRISPR1-STY array and *cas* operon. Moreover, in *C. freundii* complex sp. CFNIH3, a truncated transposase was found 30 kb upstream of the CRISPR1 loci. The region between transposase and CRISPR1 shared 40% similarity with that of *S. enterica* subsp. *enterica* serovar Typhimurium, indicating an occurrence of HGT event (Fig. S13). The split of *Salmonella* serovars into two separate clades and clubbing of serovar of CRISPR1-STM with *Shigella* and *E. coli* was also observed in the Cas1 phylogram reported by Touchon et al*.*^8^ thus, conforming to our results.

With the comprehensive analysis of all the results, we put forward the following hypotheses for evolution of CRISPR-Cas system in *Salmonella*. Given that a good proportion of *Escherichia, Shigella,* and *Klebsiella* strains harbor CRISPR1-STM/*cas*-STM type leader and operon (Table S4), we hypothesize that the LCA of the array for *Enterobacteriaceae* family could have been CRISPR1-STM/*cas*-STM type. Moreover, after the divergence from these genera, *Salmonella* could have laterally acquired its CRISPR2 array, as there exists no similarity in their leader sequences, while leaders of *S. enterica* and *S. bongori* are 78% identical and well conserved. *S. enterica* subsp. *arizonae* and subsp. *diarizonae* do not have a CRISPR2 array, which could have been possibly lost in due course of evolution. Many strains of subsp. *arizonae* do not contain the CRISPR1 array suggesting its loss as well. We also observed substantial conservation of CRISPR2-leader throughout *S. enterica subsp. enterica*. With this background, we propose the following. Apparently one, few or all the serovars belonging to the CRISPR1-STY/*cas*-STY clade could have acquired CRISPR1-STY leader and *cas*-STY operon from an unknown source, possibly by HGT event in the gut of primates, subsequently transmitting amongst other *Salmonella* strains or other genera whereas the CRISPR2 leader remained unaffected. However, one cannot rule out similar possibility for CRISPR1-STM/*cas*-STM type system. The inheritance of the CRISPR1-STY/*cas*-STY system perhaps renders competitive advantage in primate gut to the strains possessing it, in terms of its pathogenicity and enhanced survival in hostile conditions. Further investigation of CRISPR-Cas evolution across the *Enterobacteriaceae* family is warranted to strengthen our hypothesis.

The results of our study hold prospects in providing insights into the evolution of *Salmonella* that has diverse host-specificity, linking various regulatory networks with the CRISPR-Cas system.

## Materials and methods

### Sequence data collection

The CRISPR and *cas* loci of 133 *Salmonella* strains were obtained in correct orientation after retrieving the data from GenBank and CRISPR-Cas++ database^54^. For details, refer to supplementary material. For MLST, sequences of seven housekeeping genes namely*, purE, hemD, aroC, dnaN, hisD, thrA* and *sucA* were retrieved from BIGSdb software^55^, and the unannotated ones were extracted from the genome’s annotation files using customized written bash script. The composite sequence tags were allocated for the allelic profiles of these seven genes.

The CRISPR leader and *cas* operon sequences of 17 strains comprising of genus *Salmonella, Escherichia, Citrobacter, Shigella, and Klebsiella* were extracted using the CRISPR-Cas++ database/CRISPRCasFinder and matched with the *Salmonella’s* CRISPR leader sequences. The criteria of CRISPR1 Leader- 65% nucleotide similarity, and Cas-35% nucleotide similarity was chosen as the values are higher than the match obtained between the CRISPR1 and Cas- STM and STY category.

### Analysis of the CRISPR-Cas components

To create spacer maps of the CRISPR arrays, the spacers were aligned and similarity calculated. A similarity of 90% was considered to maximize their homology across the *Salmonella* strains to construct the spacer map. The intra- and inter-serovar spacer conservation were estimated using python scripts. The orientation of the individual *cas* genes was traced and the sequence similarity calculated using a custom python script. The amino acid sequences of Cse1 and the essential domains of Cas3 protein (HD domain, helicase C terminal domain, and the DEAD-box) of *Salmonella* were extracted from the Uniprot database and aligned with the reported sequences of *E. coli* using the tool Clustal Omega.

The sequence logo for the CRISPR leader and DR sequences^54^ were generated using the tool WEBLOGO ver 2.8.2^56^. The MGEs elements were manually checked 50 kb upstream and downstream of each CRISPR loci using the annotated GenBank files. Further, the GC content of the CRISPR-Cas components, and the whole genome was computed using python script.

### Phylogenetic analysis

For the CRISPR leader and *cas* operon, multiple sequence alignment were performed on the aforesaid sequences by MUSCLE version 3.6 with default parameters^57^ integrated into Molecular Evolutionary Genetics Analysis version 10 (MEGA X)^58^. All positions with alignment gaps and missing data were excluded (complete deletion option). The resulting alignments of respective groups of sequences were used to construct each phylogenetic tree using the Maximum Likelihood (ML) method^59^ guided by the most suitable evolutionary model proposed by Bayesian approach^60^. The trees were given confidence with a bootstrap value of 1000 iterations. The substitution models and the parameters used for the reconstructed trees were Tamura-Nei model with Gamma distribution for MLST; Tamura 3-parameter model for CRISPR1-leader and CRISPR2-Leader and Kimura2-parameter model along with gamma distribution for concatenated *cas* genes and *cas3* gene. The Newick format of the trees was used for further visualization and analyses through MEGA X. All trees were drawn to scale, and the branch lengths were calculated as the number of substitutions per site.

The phenograms for the CRISPR1 and CRISPR2 spacers was constructed based on presence-absence matrix. The spacers for each strain were considered as present if they had 90% sequence similarity. Using this a Jaccard similarity matrix was created. The Jaccard distance was computed on the basis of this matrix and the phenogram was created using the neighbour joining method in MEGAX^58^.

For topology validation, the phylogenetic trees for *Salmonella* were also reconstructed using the program PHYML version 3.1^61^ with statistical tests for branch support The statistical parametric analysis of Shimodaira-Hasegawa re-estimation of log-likelihood was adopted to get the consensus maximum likelihood tree. The general time reversible substitution models were kept uniform for all the trees generated. Curation of the multiply aligned sequences was done through GBlocks, having, a stringent selection of many contiguous non-conserved positions being disallowed^62^.

### Protospacer analysis

The spacer sequences for a particular serovar was extracted from the CRISPR-Cas++ database in the .fna format and the data of all the strains was combined to obtain a unique set of spacers. The files were then uploaded in the CRISPRTarget tool^39^ to get the protospacer target hits. The data was extracted for Genbank Phage, RefSeq-Plasmid and IMGVR databases. A heat map was created by matching the accession number (as detailed in supplementary methodology) of the protospacer targets.

## Supplementary information

Supplementary Information.
