# Supplementary material for: The phylogenomics of CRISPR-Cas system and revelation of its features in Salmonella
Source: Sci Rep. 2020 Dec 3;10:21156. doi: 10.1038/s41598-020-77890-6 (PMC7712790; doi:10.1038/s41598-020-77890-6)
Supplement: Supplementary file 1 — Supplementary Information. [file 41598_2020_77890_MOESM1_ESM.pdf]

Supplementary material for "The Phylogenomics of CRISPR-Cas System and revelation of its features in *Salmonella*"

Simran Krishnakant Kushwaha[1*], Narra Lakshmi Sai Bhavesh[1#], Bahaa Abdella[2,3#], Chandrajit Lahiri[2] and Sandhya Amol Marathe[1*]

Supplementary materials include:

Table S1-S8

Supplementary figure legends

Figures S1-S13

Supplementary Methodology

Supplementary Table S1. Name, accession number for whole genome sequence and spacer details for the strains analysed in the study.

STRAIN NAME	ACCESSION NUMBER	No. OF SPACERS-CRISPR1	No. OF SPACERS-CRISPR2
Salmonella enterica subsp. *enterica*			
Serovar Anatum	Broad-host range		
**USDA-ARS-USMARC-1175**	**CP007483.2**	**2**	**26**
USDA-ARS-USMARC-1676	CP014620.1	2	19
USDA-ARS-USMARC-1677	CP014663.2	2	21
USDA-ARS-USMARC-1727	CP014621.2	2	12
USDA-ARS-USMARC-1728	CP014664.1	2	25
USDA-ARS-USMARC-1735	CP007584.2	8	8
USDA-ARS-USMARC-1736	CP014657.1	8	26
USDA-ARS-USMARC-1765	CP014659.2	2	21
USDA-ARS-USMARC-1766	CP014665.1	8	24
**USDA-ARS-USMARC-1781**	**CP014666.2**	**8**	**20**
**USDA-ARS-USMARC-1783**	**CP014661.1**	**2**	**25**
**ATCC BAA-1592**	**CP007531.1**	**8**	**20**
CDC 06-0532	CP007211.2	8	20
Serovar Enteritidis	Broad-host range		
**77-1427**	**CP007598.1**	**9**	**8**
CDC_2010K_0968	CP007528.1	9	11
EC20090641	CP007249.2	9	11
EC20090698	CP007248.1	9	11
EC20110221	CP007247.1	9	11
EC20110354	CP007175.1	9	12
EC20110355	CP007250.1	9	12
EC20110356	CP007262.1	9	12
EC20110357	CP007261.1	9	11
EC20110358	CP007260.1	9	11
EC20110360	CP007258.1	9	12
EC20110361	CP007263.1	9	12
EC20111095	CP007254.1	9	12
EC20111174	CP007253.1	9	12
EC20111175	CP007252.1	9	12
EC20120002	CP007329.2	9	12
**EC20120005**	**CP007267.2**	**9**	**12**
EC20120008	CP007245.1	9	11
EC20121175	CP007269.2	9	12
**P125109**	**AM933172.1**	**8**	**10**
Serovar Saintpaul	Broad-host range		
**CFSAN004173**	**CP019204.1**	**15**	**20**
CFSAN004174	CP019206.1	15	20
CFSAN004175	CP019172.1	15	20
SGB23	CP023166.1	11	22
**SARA26**	**CP017727.1**	**41**	**22**
Serovar Heidelberg	Broad-host range		
CFSAN002064	CP005995.1	25	18
**41578**	**CP004086.1**	**25**	**16**
B182	CP003416.1	25	18
**CFSAN002069**	**CP005390.2**	**25**	**18**
**SARA35**	**CP019176.1**	**25**	**18**
SL476	CP001120.1	25	18
Serovar Typhi	Host-restricted- Primates		
**P-stx-12**	**CP003278.1**	**6**	**1**
**Ty2**	**AE014613.1**	**6**	**1**
**CT18**	**AL513382.1**	**6**	**1**
1016889	LT904893.1	6	1
1036491	LT904885.1	6	1
129-0238-M	LT904888.1	6	1
ERL034151	LT904867.1	7	1
Serovar Newport II	Broad-host range		
**CVM 21538**	**CP010282.1**	**26**	**19**
CVM 21550	CP010283.1	26	19
CVM 22425	CP010279.1	26	19
CVM 22462	CP010280.1	26	19
**CVM 22513**	**CP010281.1**	**20**	**19**
CVM N1543	CP010284.1	26	19
CVM N18486	CP009561.1	17	12
**SL254**	**CP001113.1**	**26**	**19**
USMARC-S3124.1	CP006631.1	26	19
WA_14882	CP016357.1	25	19
Serovar Newport III	Broad-host range		
CFSAN001660	CP016010.1	15	20
CDC 2009K-1331	CP025248.1	13	16
CDC 2012K-0938	CP025246.1	4	20
Levine 1	CP015923.1	13	19
**USDA-ARS-USMARC-1927**	**CP007216.2**	**18**	**10**
**Levine 15**	**CP015924.1**	**5**	**16**
Serovar Agona	Broad-host range		
**SL483**	**CP001138.1**	**18**	**8**
Serovar Bovismorbificans	Broad-host range		
**3114**	**HF969015.2**	**24**	**15**
Serovar Paratyphi A	Host-restricted-Primates		
**AKU_12601**	**FM200053.1**	**7**	**3**
**ATCC 11511**	**CP019185.1**	**7**	**3**
**ATCC 9150**	**CP000026.1**	**5**	**3**
Serovar Gallinarum	Host-restricted- Poultry and other avains		
**287/91**	**AM933173.1**	**2**	**10**
**9184**	**CP019035.1**	**2**	**10**
Serovar Pullorum	Host-restricted- Poultry		
**ATCC 9120**	**CP012347.1**	**2**	**6**
**S06004**	**CP006575.1**	**2**	**2**
Serovar Gallinarum/Pullorum	Host-restricted- Poultry		
**CDC1983-67**	**CP003786.1**	**2**	**6**
**RKS5078**	**CP003047.1**	**2**	**6**
Serovar Typhimurium	Broad-host range		
USDA-ARS-USMARC-1808	CP014969.1	10	26
USDA-ARS-USMARC-1810	CP014982.2	21	31
USDA-ARS-USMARC-1880	CP014981.1	11	35
USDA-ARS-USMARC-1896	CP014977.1	28	24
USDA-ARS-USMARC-1898	CP014971.2	22	32
USDA-ARS-USMARC-1899	CP007235.2	8	19
**str. 14028S**	**CP001363.1**	**22**	**26**
str. 798	CP003386.1	22	22
CDC 2009K-1640	CP014975.1	10	26
CDC 2009K-2059	CP014983.1	28	24
CDC 2010K-1587	CP014965.1	27	24
CDC 2011K-0870	CP007523.1	11	34
CDC 2011K-1702	CP014967.1	10	26
D23580	LS997973.1	21	18
**DT104**	**HF937208.1**	**10**	**26**
DT2	HG326213.1	20	26
L-3553	AP014565.1	22	15
**SARA13**	**CP017728.1**	**17**	**25**
**SL1344**	**FQ312003.1**	**14**	**23**
ST4/74	CP002487.1	14	23
T000240	AP011957.1	20	32
UK-1	CP002614.1	14	23
CFSAN001921	CP006048.1	27	24
Serovar Sendai	Host-restricted- Primates		
**NCTC5772**	**NZ_UGVR01000002.1**	**4**	**1**
Serovar Tennessee	Broad-host range		
**ATCC 10722**	**CP025218.1**	**63**	**23**
CFSAN001387	CP014994.1	47	21
CFSAN070643	CP024168.1	63	23
CFSAN076210	CP033345.1	47	21
PIR00537	CP025217.1	47	23
CFSAN070645	CP024164.1	59	21
**TXSC_TXSC08-19**	**CP007505.1**	**41**	**23**
Serovar Dublin	Host-adapated-Cattle		
**CT_02021853**	**CP001144.1**	**2**	**5**
Serovar Paratphi C	Host-restricted- Primates (not frequent) and known to infect other animals like pigs		
**RKS4594**	**CP000857.1**	**10**	**9**
Serovar Montevideo	Broad-host range		
**CFSAN051296**	**CP029336.1**	**24**	**17**
**531954**	**CP029035.1**	**24**	**17**
507440-20	CP007530.1	24	17
CDC 07-0954	CP017974.1	11	25
CDC 08-1942	CP017975.1	6	21
CDC 2010K-0257	CP020912.1	24	17
CDC 2011K-1674	CP017976.1	11	21
CDC 2012K-1544	CP017977.1	24	17
CDC 2013K-0218	CP017978.1	6	21
FCC0123	CP040379.1	4	22
CDC 2009K-0792	CP020752.1	35	20
USDA-ARS-USMARC-1900	CP017970.1	36	22
USDA-ARS-USMARC-1901	CP017971.1	34	20
USDA-ARS-USMARC-1904	CP017972.1	33	20
USDA-ARS-USMARC-1912	CP017973.1	35	16
**USDA-ARS-USMARC-1903**	**CP007222.1**	**35**	**23**
**USDA-ARS-USMARC-1921**	**CP007540.2**	**35**	**23**
Salmonella enterica subsps. arizonae	Host-cold-blooded animals, mainly reptiles and sheeps		
**NCTC10047**	**LR134156.1**	**23**	**ABSENT**
Salmonella enterica subsps. diarizonae	Host-cold-blooded animals, mainly reptiles and sheeps		
**50 MZ0080**	**CP022142.1**	**29**	**ABSENT**
Salmonella bongori	Host-cold-blooded animals, have been isolated from humans but very rare		
**CFSAN000510**	**CP045766.1**	**20**	**17**
**NCTC12419**	**LR134137.1**	**20**	**17**
**66 SA19983605**	**CP022120.1**	**20**	**17**

The strains represented in bold were considered for further analysis. The strains highlighted in grey for serovars Montevideo and Saintpaul represent the ones having distinct spacer sets.

Supplementary Table S2. The statistics of the spacer index for the serovars under consideration.

			No. of strains analysed	CRISPRI*			CRISPR2*		
				Minimum	Maximum	Average	Minimum	Maximum	Average
Salmonella enterica subsp. *enterica*	Host-resticted	Typhi	7	6	7	6.14	1	1	1
		Paratyphi A	3	5	7	6.33	3	3	3
		Gallinarum	2	2	2	2	10	10	10
		Pullorum	2	2	2	2	2	6	4
		Gallinarum/Pullorum	2	2	2	2	6	6	6
		Sendai	1	4	4	4	1	1	1
	Host-adapted	Dublin	1	2	2	2	5	5	5
	Broad-host range	Anatum	13	2	8	4.77	8	26	20.25
		Paratyphi C	1	10	10	10	9	9	9
		Heidelberg	6	25	25	25	16	18	17.67
		Newport II	10	17	26	24.4	12	19	18.3
		Newport III	6	4	18	11.33	10	20	16.83
		Enteritidis	20	8	9	8.95	8	12	11.35
		Typhimurium	23	8	28	17.95	15	35	25.39
		Bovismorbificans	1	24	24	24	15	15	15
		Tennessee	7	41	63	52.43	21	23	22.14
		Montevideo	17	4	36	23.59	16	25	19.94
		Saintpaul	5	11	4 1	19.4	20	22	20.8
		Agona	1	18	18	18	8	8	8
	Cold-blooded hosts	*Salmonella enterica* subsp. *arizonae*	1	23	23	23		-	-
		Salmonella enterica subsp. *diarizonae*	1	29	29	29	-	-	-
		Salmonella bongori	3	20	20	20	17	17	17
		Total				15.29			12.58

* Each serovar has one CRISPR1 and one CRISPR2 array except *S. enterica* subsp. *arizonae* and *diarizonae*

Supplementary Table S3. Name and accession number for whole genome sequence for the strains analysed in the study.

SPECIES	ACCESSION NUMBER	ABBREVATION
Salmonella bongori str. 66 SA19983605	CP022120.1	SB
Salmonella enterica subsps. *arizonae* str. NCTC10047	LR134156.1	SE-A
Salmonella enterica subsps. *diarizonae* 50 MZ0080	CP022142.1	SE-D
Salmonella enterica subsp. *enterica* serovar Typhimurium str. 14028s	CP001363.1	STM1
Salmonella enterica subsp. *enterica* serovar Typhimurium str. SARA13	CP017728.1	STM2
Salmonella enterica subsp. *enterica* serovar Typhi str. Ty2	AE014613.1	STY1
Salmonella enterica subsp. *enterica* serovar Typhi str. CT18	AL513382.1	STY2
Salmonella enterica subsp. *enterica* serovar Newport str. CVM21538	CP010282.1	SE-NP1
Salmonella enterica subsp. *enterica* serovar Newport str. CVM22513	CP010281.1	SE-NP2
Klebsiella pneumoniae subsp. *pneumoniae* strain TGH10	CP012744.1	KP1
Klebsiella pneumoniae strain INF235-sc-2280127	CP031817.1	KP2
Shigella boydii strain ATCC 49812	CP026836.1	SB1
Citrobacter freundii complex sp. CFNIH3	CP026235.1	C1
Citrobacter freundii complex sp. CFNIH9	CP026238.1	C2
Citrobacter sp. 30_2	CP022311.1	C3
Escherichia coli strain 52148	CP050382.1	EC1
Escherichia coli strain SCU-118	CP051716.1	EC2

The CRISPR1 leader sequences of these strains were used for the phylogenetic analysis. The abbreviations are the key to the figure.

Supplementary Table S4. Strains of *Klebsiella*, *Escherichia* and *Shigella* matching with CRISP1-STM leader and *cas*-STM.

	Escherchia	*Klebsiella*	*Shigella*
CRISPR1-STM Leader	491	105	12
Cas-STM	~1000	~100	~50

Values indicate number of strains.

Supplementary Table S5. MGE candidates flanking the CRISPR-Cas system.

	Genome location (Loci start and Loci end)			MGE	Percentage GC Content				
	CRISPR2	*cas*	CRISPR1	Transposase/ Helicase	CRISPR2 loci	*cas* operon	CRISPR1 loci	CRISPR-Cas	Whole genome
Paratyphi A str. AKU_12601	2902111-2902322	2885645-2894598	2885105-2885560	2856664-2857091 and 3007579-3008037	51.3*	49.9	53.2*	49	52.18
Newport II str. SL254	3073142-3074328	3056558-3064452	3054859-3056473	3024723-3025160	54.2	50.4	55.6	51	52.22
Newport III str. USDA-ARS-USMARC-1927	975001-975639	983339-991789	991886-993012	1020178-1020414	51.3	52	57.2	51	52.18
Heidelberg str. SL476	3069137-3070263	3052976-3061435	3051217-3052879	3022734-3022874	56.3	53.1	56.2	53	52.07
Enteritidis str. EC20121175	2967508-2968269	2951453-2959906	2950779-2951356	2923727-2923816	55.8	52.8	55.7	51	52.17
Gallinarum str. 9184	1224776-1225415	1233429-1241471	1241469-1241716	1271625-1271848	56.8	53.3	47.6*	51	52.2
Pullorum str. ATCC 9120	3871330-3871722	3855356-3863734	3855036-3855283	3827981-3828070	53.6*	52.9	48.4*	51	52.19
Gallinarum/Pullorum str. CDC1983-67	2947545-2947937	2931570-2939948	2931250-2931497		53.6*	52.9	48.4*	51	52.23
Montevideo-STY str. USDA-ARS-USMARC-1900	1003049-1004420	1011949-1021097	1021182-1023406	1050505-1050672	57.7	50.4	57	52	52.35
Montevideo-STM str. CDC 2010K-0257	992948-994014	1010602-1010052	1010149-1011641	1038743-1038910	56.6	52.4	55.9	51	52.21
Bovismorbificans str. 3114	2976895-2977839	2960410-2969354	2958839-2960331		54	50	55.6	50	52.16
Anatum str. CDC 06-0532	970192-971440	979153-987612	987709-988225	1018009-1018429	56.2	52.8	55.5	51	52.18
Tennessee str. ATCC 10722	963889-965320	972914-981858	981943-985815	1015542-1015960	59.3	50.4	57.1	52	52.23
Saintpaul-STM str. CFSAN004173	946615-947863	955563-964025	964122-965066	993235-993458	57.2	52	57.4	51	52.21
Saintpaul-STY str. SARA26	944744-946114	953779-962723	962808-965337	993600-993740	58.2	50.3	56.5	51	52.05
Dublin str. CT_02021853	3137409-3137742	3121348-3129807	3121100-3121350	3090967-3091107	51.6*	53	44.6*	51	52.18
Agona str. SL483	3005517-3006033	2989328-2997778	2988105-2989231	2956649-2956789 and 2975984-2977192	54.2	52	56.3	51	52.08
Typhimurium str. CFSAN001921	473159-474652	482228-490687	490784-492564	522104-522291 and 423309-425144	55.9	50.4	56.9	51	52.17
Typhi str. CT18	2943208-2943716	2926652-2935104	2926182-2926567	2898592-2899034 and 3013615-3014073	39.8*	50.4	57.3	50	52.05
Subsp. *arizonae*		962736-971156	971253-972682			52.9	57.4	52	51.38
Subsp. *diarizonae*		1126557-1134989	1135086-1136883			52.9	56.7	53	51.54
S. *bongori*	922139-923204	930621-939089	939186-940434		53.4	52.4	54.5	50	51.33

*The lower GC content of CRISPR arrays due to the AT-rich leader sequences are represented by asterisks

Supplementary Table S6. MGE present in the whole genome of *S. enterica* subsp. *enterica* serovar Typhimurium str. LT2 and serovar Typhi str. TY2

Salmonella Typhimurium str. LT2			*Salmonella* Typhi str. TY2		
**Gene Start**	**Gene End**		**Gene Start**	**Gene End**	
110002	112908	Helicase	109791	112697	Helicase
218296	220783	Helicase	115562	116002	putative IS element transposase
340748	340953	Transposase	116022	117230	putative IS element transposase
341175	341460	Integrase	271067	271525	transposase for insertion sequence element IS200
369198	369998	Transposase	222741	225182	Helicase
405293	408272	Helicase	280079	280537	transposase for insertion sequence element IS200
489948	490109	Transposase	305010	306344	Helicase
537933	538874	Transposase	464716	465174	transposase for insertion sequence element IS200
613596	613797	Integrase	528278	528736	transposase for insertion sequence element IS200
613753	614167	Transposase	673526	673984	transposase for insertion sequence element IS200
781631	782197	Integrase	712019	713779	Helicase
885578	886953	Helicase	1070157	1070768	Helicase
887150	889305	Helicase	1070777	1071787	Helicase
962615	963952	Transposase Integrase	1139104	1141014	Helicase
969994	971585	Helicase	1189317	1189775	transposase for insertion sequence element IS200
1025163	1025627	Transposase	1220753	1221211	transposase for insertion sequence element IS200
1026090	1026773	Transposase Integrase	1265128	1265586	transposase for insertion sequence element IS200
1098231	1099531	Transposase Integrase	1450560	1451018	transposase for insertion sequence element IS200
1166406	1168476	Helicase	1598822	1602724	Helicase
1214284	1215158	Helicase	1647002	1647460	transposase for insertion sequence element IS200
1731328	1735230	Helicase	1745409	1745867	transposase for insertion sequence element IS200
1748282	1749655	Helicase	1802131	1802589	transposase for insertion sequence element IS200
1918512	1920433	Helicase	1895018	1895476	transposase for insertion sequence element IS200
1956851	1957714	Transposase	1898230	1898688	transposase for insertion sequence element IS200
1957832	1958200	Integrase	1903289	1905343	Helicase
1967023	1967919	Integrase	1970990	1972330	Integrase
1989051	1990071	Helicase	2473258	2473716	transposase for insertion sequence element IS200
1990070	1990693	Helicase	2534377	2534835	transposase for insertion sequence element IS200
2045732	2046196	Transposase	2735202	2736233	Integrase
2324547	2326307	Helicase	3012372	3012830	transposase for insertion sequence element IS200
2579330	2579794	Transposase	3043685	3045148	Integrase
2622291	2622652	Transposase	3052442	3053338	Integrase
2731352	2731902	Transposase	3077594	3078052	transposase for insertion sequence element IS200
2775590	2776825	Integrase	3085185	3085643	transposase for insertion sequence element IS200
2782896	2784240	Helicase	3108025	3108483	transposase for insertion sequence element IS200
2876819	2877844	Integrase	3287491	3289431	Helicase
2878160	2879237	Integrase	3440746	3442575	Helicase
2900435	2901318	Integrase	3447448	3449610	Helicase
2905473	2906075	Integrase	3450462	3451364	Integrase
2906456	2906923	Integrase	3482595	3483860	Helicase
2907070	2907343	Transposase	3485500	3487524	Helicase
2908424	2910413	Helicase	3501128	3502180	Integrase
2910673	2910984	Transposase	3894835	3896916	Helicase
2910967	2911847	Transposase	4296365	4297780	Helicase
3081922	3082490	Transposase	4395333	4396697	Helicase
3084047	3086721	Helicase	4489831	4490856	Integrase
3144282	3146128	Helicase	4511590	4512603	Integrase
3146114	3149659	Helicase	4524556	4525815	Integrase
3152718	3156100	Helicase	4666742	4668007	Integrase
3194324	3194788	Transposase	4689323	4693072	Helicase
3204431	3205339	Integrase	4693110	4695647	Helicase
3317055	3317570	Helicase			
3446192	3448132	Helicase			
3635168	3635632	Transposase			
3936873	3938974	Helicase			
4049846	4051066	Integrase			
4119776	4121800	Helicase			
4123440	4124705	Helicase			
4153785	4154702	Integrase			
4155554	4157716	Helicase			
4162571	4164418	Helicase			
4466589	4468022	Helicase			
4559426	4559890	Transposase			
4702530	4702982	Transposase			
4731324	4731619	Integrase			
4731975	4735496	Helicase			

Supplementary Table S7. Mapping protospacer sources of CRISPR spacers

			CRISPR1					CRISPR2				
		Species	No. of spacers having protospacers	No. of spacers not displaying any protospacers	Total No. of spacers	Percent spacers having protospacers	Percent spacers not displaying protospacers	No. of spacers having protospacers	No. of spacers not displaying any protospacers	Total No. of spacers	Percent spacers having protospacers	Percent spacers not displaying protospacers
Salmonella enterica subsp. *enterica*	Host-resticted	Typhi	5	2	7	71.43	28.57	1	0	1	100.00	0.00
		Paratyphi A	5	2	7	71.43	28.57	2	1	3	66.67	33.33
		Gallinarum	1	1	2	50.00	50.00	6	4	10	60.00	40.00
		Pullorum	2	1	3	66.67	33.33	3	3	6	50.00	50.00
		Gallinarum/Pullorum	2	0	2	100.00	0.00	3	3	6	50.00	50.00
		Sendai	4	0	4	100.00	0.00	1	0	1	100.00	0.00
	Host-adapted	Dublin	1	1	2	50.00	50.00	2	3	5	40.00	60.00
	Broad-host range	Anatum	4	4	8	50.00	50.00	18	8	26	69.23	30.77
		Paratyphi C	6	4	10	60.00	40.00	4	5	9	44.44	55.56
		Heidelberg	18	7	25	72.00	28.00	13	5	18	72.22	27.78
		Newport II	16	11	27	59.26	40.74	15	6	21	71.43	28.57
		Newport III	10	8	18	55.56	44.44	12	9	21	57.14	42.86
		Enteritidis	4	5	9	44.44	55.56	9	3	12	75.00	25.00
		Typhimurium	16	13	29	55.17	44.83	25	12	37	67.57	32.43
		Bovismorbificans	17	7	24	70.83	29.17	9	6	15	60.00	40.00
		Tennessee	37	26	63	58.73	41.27	16	7	23	69.57	30.43
		Montevideo-STM	16	9	25	64.00	36.00	12	15	27	44.44	55.56
		Montevideo-STY	26	10	36	72.22	27.78	18	5	23	78.26	21.74
		Saintpaul-STM	10	5	15	66.67	33.33	13	9	22	59.09	40.91
		Saintpaul-STY	27	14	41	65.85	34.15	13	9	22	59.09	40.91
		Agona	8	10	18	44.44	55.56	5	3	8	62.50	37.50
	Cold-blooded hosts	*Salmonella enterica* subsp. *arizonae*	18	5	23	78.26	21.74	NA	NA	NA	NA	NA
		Salmonella enterica subsp. *diarizonae*	18	11	29	62.07	37.93	NA	NA	NA	NA	NA
		Salmonella bongori	9	11	20	45.00	55.00	9	8	17	52.94	47.06
		AVERAGE				63.92	36.08				64.07	35.93
		STANDARD DEVIATION				14.80	14.80				15.6	15.6

Supplementary Table S8. Mapping the ChIP-seq of Cas5 on the whole genome of *S. enterica* subsp. *enterica* serovar Typhimurium str. 14028S

ChIP-seq Peak Center	Gene name	Function
**648399**	***entF***	**Enterobactin synthetase, component F[1]**
**658872**	***entE***	**Enterobactin synthetase, component E[2]**
**1482744**	***ttrS***	**Tetrathionate sensor histidine kinase[3]**
**1048355**	***ompF***	**Forms pores that allow passive diffusion of small molecules across the outer membrane[4]**
**2724461**	***sseA***	**Type III secretion system chaperone[5]**
**2747746**	***hmpA***	**Is involved in NO detoxification in an aerobic process, termed nitric oxide dioxygenase (NOD) reaction that utilizes O2Êand NAD(P)H to convert NO to nitrate, which protects the bacterium from various noxious nitrogen compounds[6]**
**2242087**	***wcaI***	**GDP-mannose mannosyl hydrolase[7]**
**2937813**	***iroC***	**ABC transporter ATP-binding protein[8]**
**3042704**	***sptP***	**Effector proteins function to alter host cell physiology and promote bacterial survival in host tissues[9]**
**3210092**	***stdB***	**Fimbrial biogenesis outer membrane usher protein[10]**
**3525028**	***sspA***	**important for enteropathogenicity[11]**
**3815385**	***bcsA***	**Catalytic subunit of cellulose synthase. It polymerizes uridine 5'-diphosphate glucose to cellulose, which is produced as an extracellular component for mechanical and chemical protection at the onset of the stationary phase, when the cells exhibit multicellular behavior[12]**
**3976737**	***mgtB***	**Mediates magnesium influx to the cytosol[13]**
**4161855**	***cyaA***	**Adenylate cyclase is involved in the regulation of cyclase activity by the carbon source[14]**
**4547246**	***basS***	**Member of the two-component regulatory system BasS/BasR. Autophosphorylates and activates BasR by phosphorylation[15]**
**4861926**	***sthD***	**Putative fimbrial subunit[16]**
**4862974**	***sthB***	**Fimbrial assembly protein[17]**
28623	*bcfD*	Cell adhesion
37110		putative 5'-nucleotidase
67716		putative citrate-sodium symporter
74672	*dapB*	Catalyzes the conversion of 4-hydroxy-tetrahydrodipicolinate (HTPA) to tetrahydrodipicolinate
78290	*carB*	Carbamoyl-phosphate synthase large chain
81565	*caiF*	DNA binding
85304	*caiB*	Catalyzes the reversible transfer of the CoA moiety from gamma-butyrobetainyl-CoA to L-carnitine to generate L-carnitinyl-CoA and gamma-butyrobetaine.
113296	*hepA*	Transcription regulator that activates transcription by stimulating RNA polymerase (RNAP) recycling in case of stress conditions such as supercoiled DNA or high salt concentrations.
114993	*polB*	DNA polymerase
134582	*leuL*	Involved in control of the biosynthesis of leucine.
136070	*leuO*	Probable HTH-type transcriptional regulator
137573	*ilvI*	Acetolactate synthase isozyme 3 large subunit
162127		
166935	*hofC*	Protein transport protein
171781		putative cytoplasmic protein
188885	*acnB*	Aconitate hydratase-Involved in the catabolism of short chain fatty acids
254924		30S ribosomal protein S2
259664	*dxr*	Catalyzes the NADP-dependent rearrangement and reduction of 1-deoxy-D-xylulose-5-phosphate (DXP) to 2-C-methyl-D-erythritol 4-phosphate (MEP)
260309	*uppS*	Ditrans,polycis-undecaprenyl-diphosphate synthaseÊ
270914	*dnaE*	DNA polymerase III is a complex, multichain enzyme responsible for most of the replicative synthesis in bacteria.
283323	*proS*	Proline--tRNA ligase
284920		
292686	*rrlH*	~
316369		putative cytoplasmic protein
331550		putative cytoplasmic protein
354530	*fadE*	Catalyzes the dehydrogenation of acyl-coenzymes A (acyl-CoAs) to 2-enoyl-CoAs, the first step of the beta-oxidation cycle of fatty acid degradation.
360797		
385712		pseudogene
391414		putative outer membrane protein
444928	*yajF*	FructoKinase
461724	*tgt*	Queuine tRNA-ribosyltransferase
503419	*tig*	Involved in protein export. Acts as a chaperone by maintaining the newly synthesized protein in an open conformation.
510542	*cypD*	Peptidylprolyl isomerase
530680	*acrB*	Multidrug efflux pump subunit drug efflux protein complex with broad substrate specificity that uses the proton motive force to export substrates.
533628	*acrA*	Multidrug efflux pump subunit drug efflux protein complex with broad substrate specificity that uses the proton motive force to export substrates.
547471		hypothetical protein
565916	*ybbO*	NAD(P)-dependent oxidoreductase
608634	*fimD*	Involved in the export and assembly of FimA fimbrial subunits across the outer membrane.
704128	*mrdA*	Catalyzes cross-linking of the peptidoglycan cell wall
781326	*ybgL*	Catalyzes the cleavage of 5-oxoproline to form L-glutamate coupled to the hydrolysis of ATP to ADP and inorganic phosphate.
799842	*sdhA*	Succinate dehydrogenase flavoprotein
805852	*sucB*	E2 component of the 2-oxoglutarate dehydrogenase (OGDH) complex which catalyzes the second step in the conversion of 2-oxoglutarate to succinyl-CoA and CO_2
862272	*bioF*	8-amino-7-oxononanoate synthase
911440		hypothetical protein
995967	*ftsK*	Essential cell division protein that coordinates cell division and chromosome segregation.
1027515	*ycaI*	Lactamase_B domain-containing protein
1071391		
1086398		minor tail protein
1088277		attachment/invasion protein
1126386	*helD*	DNA Helicase
1146356		hypothetical protein
1173716		putative transcriptional regulator
1175296		putative sodium/glucose cotransporter
1197202	*mdoH*	Involved in the biosynthesis of osmoregulated periplasmic glucans
1230875	*rluC*	Ribosomal large subunit pseudouridine synthase C
1237741	*fabG*	Catalyzes the NADPH-dependent reduction of beta-ketoacyl-ACP substrates to beta-hydroxyacyl-ACP products, the first reductive step in the elongation cycle of fatty acid biosynthesis
1250794	*nagZ*	Plays a role in peptidoglycan recycling by cleaving the terminal beta-1,4-linked N-acetylglucosamine (GlcNAc)
1319463		phage tail component H-like protein
1339141		
1362889		
1371489	*yeaH*	~
1379980	*yeaA*	Peptide methionine sulfoxide reductase
1393323	*argD_1*	Catalyzes the transamination of N2-succinylornithine and alpha-ketoglutarate into N2-succinylglutamate semialdehyde and glutamate
1409579		hypothetical protein
1427820	*pheT*	Phenylalanine--tRNA ligase beta subunit
1435560	*aroH*	Stereospecific condensation of phosphoenolpyruvate (PEP) and D-erythrose-4-phosphate (E4P) giving rise to 3-deoxy-D-arabino-heptulosonate-7-phosphate (DAHP).
1573929	*ynfM*	Permease
1575922	*mlc*	~
1599510	*ydfG*	NADP-dependent dehydrogenase with broad substrate specificity acting on 3-hydroxy acids
1609053	*ydeA*	Involved in the efflux of sugars.
1613004	*yneH*	Glutaminase
1662035	*fdnG*	Formate dehydrogenase allows E.coli to use formate as major electron donor during anaerobic respiration, when nitrate is used as electron acceptor
1707334		putative benzoate membrane transport protein
1749855		
1875758	*narX*	Sensor protein
1902575		putative cytochrome oxidase subunit I
1966259		
2022871		
2152720	*cbiP*	Cobyric acid synthase Catalyzes amidations at positions B, D, E, and G on adenosylcobyrinic A,C-diamide. NH2 groups are provided by glutamine, and one molecule of ATP is hydrogenolyzed for each amidation.
2174789	*pduJ*	Carboxysome shell protein
2286468		
2328373		
2340192	*galS*	Repressor of the mgl operon. Binds galactose and D-fucose as inducers. GalS binds to an operator DNA sequence within its own coding sequence
2402408	*ccmF_1*	Cytochrome c-type biogenesis protein F1
2454950	*ais*	Lipopolysaccharide core heptose(II)-phosphate phosphatase isinvolved in the pathway lipopolysaccharide metabolism, which is part of Bacterial outer membrane biogenesis.
2502812	*pta*	Involved in acetate metabolism
2562245	*pgtC*	Required for PgtP expression, it may act jointly with the PgtA/PgtB signaling proteins.
2612828	*hemF*	Involved in the heme biosynthesis
2615387		putative cytoplasmic protein
2676031	*guaB*	Catalyzes the conversion of inosine 5'-phosphate (IMP) to xanthosine 5'-phosphate (XMP), the first committed and rate-limiting step in the de novo synthesis of guanine nucleotides, and therefore plays an important role in the regulation of cell growth.
2690387	*ratB*	Involved in toxin-antitioxin system
2696323	*ratA*	Ribosome association toxin
2708378	*ispG*	4-hydroxy-3-methylbut-2-en-1-yl diphosphate synthase
2723080		putative inner membrane lipoprotein
2759144	*yfhK*	Histidine Kinase
2772009		putative permease
2795645		phage tail component H-like protein
2852910	*rrlG*	~
2878401	*recN*	May be involved in recombinational repair of damaged DNA
2932715		

2935299	*hin*	DNA-invertase hin
2946034		
2959109	*ygaF*	Multi functional fusion protein
2968949		putative inner membrane protein
2993084	*alaS*	Catalyzes the attachment of alanine to tRNA(Ala)
3007285	*ygbD*	One of at least two accessory proteins for anaerobic nitric oxide (NO) reductase
3090276	*truD*	tRNA pseudouridine synthase D
3092114		hypothetical protein
3093634	*cysN*	Sulfate adenylyltransferase subunit 1-May be the GTPase, regulating ATP sulfurylase activity.
3110917	*cysJ*	Component of the sulfite reductase complex that catalyzes the 6-electron reduction of sulfite to sulfide.
3125034	*rumA*	23S rRNA (uracil(1939)-C(5))-methyltransferase
3132946	*ygcY*	Glucarate dehydratase
3151410	*fucU*	Involved in the anomeric conversion of L-fucose.
3153348	*ygdE*	Catalyzes the 2'-O-methylation at nucleotide C2498 in 23S rRNA.
3226541	*ygfZ*	tRNA modifying protein
3227626	*yqfA*	Cytolysis
3305087		putative amino acid transporter
3335029		
3408554	*ygjQ*	DUF218 domain-containing protein
3422879	*yhaO*	amino acid transmembrane transport
3435898	*garL*	Catalyzes the reversible retro-aldol cleavage of both 5-keto-4-deoxy-D-glucarate and 2-keto-3-deoxy-D-glucarate to pyruvate and tartronic semialdehyde.
3468956	*infB*	Translation initiation factor IF-2
3471285	*yhbC*	Ribosome maturation factor
3475679	*glmM*	Catalyzes the conversion of glucosamine-6-phosphate to glucosamine-1-phosphate
3477559	*folP*	Catalyzes the condensation of para-aminobenzoate (pABA) with 6-hydroxymethyl-7,8-dihydropterin diphosphate (DHPt-PP) to form 7,8-dihydropteroate (H2Pte)
3479495	*hflB*	Acts as a processive, ATP-dependent zinc metallopeptidase for both cytoplasmic and membrane proteins.
3487508	*ispB*	Octaprenyl diphosphate synthase
3513963	*gltD*	Glutamate synthase
3523435	*yhcK*	HTH-type transcriptional repressor
3558707		hypothetical protein
3560950	*yhdH*	Putative oxidoreductase
3583298	*rrlD*	~
3589371	*smg*	~
3602356		
3618205	*yheO*	Putative reulator
3619144	*fkpA*	Peptidyl-prolyl cis-trans isomerase
3635929		Hypothetical protein
3658225	*hofQ*	DNA transporter
3685793	*malQ*	Transfers a segment of a (1->4)-alpha-D-glucan to a new position in an acceptor, which may be glucose or a (1->4)-alpha-D-glucan.
3690088	*malP*	Alpha-1,4 glucan phosphorylase
3729165		
3773648	*pitA*	Phosphate transporter
3783533	*gor*	Glutathione reductase
3795727	*yhjD*	Inner membrane protein
3820332	*yhjU*	Cellulose biosynthesis protein
3850991		hypothetical protein
3851150		
3856794		putative outer membrane lipoprotein
3862386	*xylR*	Catalyzes the phosphorylation of N-acetyl-D-glucosamine (GlcNAc) derived from cell-wall degradation, yielding GlcNAc-6-P
3863537	*bax*	~
3865552	*malS*	Alpha-amylase
3878721		putative regulatory protein
3904893		Hypothetical protein
3905779		putative mandelate racemase
3934110	*kdtA*	Involved in lipopolysaccharide (LPS) biosynthesis.
3940960	*rph*	Phosphorolytic exoribonuclease that removes nucleotide residues following the -CCA terminus of tRNA and adds nucleotides to the ends of RNA molecules by using nucleoside diphosphates as substrates.
3949816	*spoT*	(P)ppGpp synthetase II
3961073	*yicJ*	MFS transporter
3963291		Hypothetical protein
3990958		putative glycosyl hydrolase
4029473	*ccmF_2*	Cytochrome c-type biogenesis protein F2
4043153	*dgoT*	D-galactonate transport protein
4058622	*dnaA*	Plays an important role in the initiation and regulation of chromosomal replication.
4094038	*atpI*	ATP F0F1 synthase subunit I
4094227	*atpI*	ATP F0F1 synthase subunit I
4095913	*gidA*	NAD-binding protein involved in the addition of a carboxymethylaminomethyl (cmnm) group at the wobble position (U34) of certain tRNAs, forming tRNA-cmnm5s2U34.
4116354	*rrlC*	~
4134314	*rep*	ATP-dependent DNA helicase
4151518	*wecF*	Catalyzes the synthesis of Und-PP-GlcNAc-ManNAcA-Fuc4NAc (Lipid III), the third lipid-linked intermediate involved in ECA synthesis.
4162972		putative inner membrane protein
4186636		putative arylsulfatase regulator
4212456	*rrlA*	~
4230132	*glnA*	Catalyzes the ATP-dependent biosynthesis of glutamine from glutamate and ammonia.
4243194	*yihQ*	Alpha-glucosidase
4248738	*yihV*	Sulfofructose kinase
4285755		
4312781	*hslU*	ATPase subunit of a proteasome-like degradation complex; this subunit has chaperone activity.
4315832	*cytR*	DNA-binding transcriptional regulator
4341382	*frwC*	PTS system fructose-like IIC component
4345376	*pflC*	Pyruvate formate lyase II activase
4353316	*argC*	Catalyzes the NADPH-dependent reduction of N-acetyl-5-glutamyl phosphate to yield N-acetyl-L-glutamate 5-semialdehyde.
4367423	*rrlB*	~
4383934	*rpoC*	DNA-dependent RNA polymerase catalyzes the transcription of DNA into RNA using the four ribonucleoside triphosphates as substrates.
4410803	*rrlE*	Probably a phage tail protein
4422051	*metH*	Methionine synthase
4422597	*metH*	Methionine synthase
4425744		
4428326		putative cytoplasmic protein
4453813	*pgi*	Glucose-6-phosphate isomerase
4498488		putative inner membrane protein
4510031		putative inner membrane protein
4512158		putative inner membrane protein
4554554		
4563616	*dcuS*	Histidine kinase
4595778	*frdC*	Two distinct, membrane-bound, FAD-containing enzymes are responsible for the catalysis of fumarate and succinate interconversion; fumarate reductase is used in anaerobic growth, and succinate dehydrogenase is used in aerobic growth.
4601159	*yjeM*	Inner membrane transporter
4603171	*yjeP*	Mechanosensitive channel protein
4606105	*psd*	Catalyzes the formation of phosphatidylethanolamine (PtdEtn) from phosphatidylserine (PtdSer)
4608075	*orn*	3'-to-5' exoribonuclease specific for small oligoribonucleotides
4619855	*hflK*	HflK could encode or regulate a protease
4630083	*yjfK*	Putative cytoplasmic protein
4651224	*ytfG*	NmrA domain-containing protein
4659723	*ytfM*	Autotransporter assembly factor
4672673		sugar transporter
4687149		putative carbohydrate kinase
4695198	*pmbA*	Metalloprotease PmbA
4696115	*cybC*	Soluble cytochrome b562-Electron-transport protein of unknown function.
4725096	*argI*	Reversibly catalyzes the transfer of the carbamoyl group from carbamoyl phosphate (CP) to the N(epsilon) atom of ornithine (ORN) to produce L-citrulline.
4743769		
4745250		putative DNA helicase
4747780		putative DNA helicase
4748007		putative DNA helicase
4778370	*yjiE*	Cell density-dependent motility repressor
4798141	*mrr*	Mrr_cat domain-containing protein
4804820		putative transcriptional regulator
4821700	*fhuF*	Ligase
4836394	*deoA*	The enzymes which catalyze the reversible phosphorolysis of pyrimidine nucleosides
4847924	*radA*	DNA-dependent ATPase involved in processing of recombination intermediates, plays a role in repairing DNA breaks.
4857384	*creB*	Response regulator in two-component regulatory system with CreC

Gene information was obtained from NCBI. The italicized sites don’t match with any gene regions. The genes in bold correspond to virulence genes (suggested by literature).

SUPPLEMENTARY FIGURE LEGENDS

Fig. S1 Spacer conservation of various serovars of *Salmonella*. Spacer conservation in **a.** CRISPR1 array and **b.** CRISPR2 array across *Salmonella* serovars. **c & d.** Inter-serovar spacer conservation in both, **c.** the CRISPR1 and **d.** CRISPR2 arrays. The colour code for a particular column represents spacer sequences with greater than 90% nucleotide similarity. The number denotes the position of the spacer from the leader sequence. The direct repeats have been eliminated for simplicity. The asterisk (*) represents the host-specific serovars. The duplication and triplication is depicted as a pattern in the coloured box. The images were generated using Microsoft Excel 365.

Fig. S2. The sequence logo of the consensus DR sequence and last direct repeat. The DR sequence of all the strains belonging to the CRISPR1-STM, CRISPR1-STY and CRISPR2 were considered and the web-logo of these were generated. The last direct repeat (distal to the leader sequence) was observed to contain SNPs. **a.** CRISPR1-STM consensus DR**, b.** CRISPR1-STM last DR**, c.** CRISPR1-STY consensus DR**, d.** CRISPR1-STY last DR**, e.** CRISPR2 consensus DR, and **f.** CRISPR2 last DR. The sequence logos were generated using WEBLOGO ver 2.8.2 available at https://weblogo.berkeley.edu/

Fig. S3. The phylogenetic trees of a. CRISPR1 leader and b. CRISPR2 leader. The phylogenetic tree were constructed using PHYML version 3.1 with statistical tests for branch support. The values indicate the aLRT scores.

Fig. S4. The sequence logos of the leader sequence for each CRISPR array. The leader sequence of all the strains were aligned and a Web-logo was generated. The CRISPR1 leader sequence of *Salmonella enterica* subsp. *enterica* is of two types CRISPR1-STM and CRISPR1-STY. The CRISPR2 leader sequence is present and highly conserved among all the serovars except *Salmonella enterica* subsp. *arizonae* and subsp. *diarizonae*. **a.** CRISPR1-STM *S. enterica* subsp. *enterica,* **b**. CRISPR1 *S. enterica* subsp. *arizonae* and subsp. *diarizonae enterica* **c.** CRISPR1 *Salmonella bongori* **d.** CRISPR1-STY *S. enterica* **e.** CRISPR2 *S. enterica* subsp. *enterica.* **f**. CRISPR2 *Salmonella bongori.* The sequence logos were generated using WEBLOGO ver 2.8.2 available at https://weblogo.berkeley.edu/

Fig. S5. Heat map representing the spacer conservation for a. CRISPR1 array b. CRISPR2 array c. both the CRISPR arrays combined. The unique set of CRISPR spacers of each serovar was compared with the remaining serovars for their presence. The spacers showing 90% sequence similarity were considered to be same. Based on this the proportion of spacers shared across serovars was analysed and heat map was made. The heat maps were created using the tool Seaborn version 0.11 available at https://seaborn.pydata.org/[1].

Fig. S6. Orientation of the CRISPR array and the *cas* operon in *Salmonella.* Five types of arrangements were evident in *Salmonella*. The *cas*-STY arrangement present in strains belonging to the CRISPR1-STY clade. The *cas*-STM type operon were subdivided into four types - *S. enterica* subsp. *enterica* (*cas*-STM, belong to CRISPR1-STM clade), *S. bongori (cas*-STM.B)*, S. enterica* subsp. *enterica*, subsp. *arizonae* (*cas*-STM.A) and subsp. *diarizonae* (*cas*-STM.D). All the strains of serovar Montevideo-STM and *S. enterica* subps. *diarizonae* str. MZ0080 (used in our study) contain a non-sense mutation in *cas3* and is represented by an asterisk (*). *Salmonella bongori* str. SA19983605 (used in our study) does not contain the *cas7* gene and is depicted by a hash (#) and all strains of *S. enterica* subsp. *arizonae* contain a stop codon in the *cas3* operon represented by a thunderbolt.

Fig. S7. The percentage conservation of the entire *cas* operon. The values in the matrix represent the percentage nucleotide match between the categories. The reference strains used were *S. enterica* serovar Typhimurium str.14028S, Typhi str. CT18, *S. enterica* subsp. *arizonae* str. NCTC10047, *S. enterica* subsp. *diarizonae* str. MZ0080 and *S. bongor*i str. SA19983605.

Fig. S8. The phylogenetic tree of *cas3* gene. The phylogenetic tree were constructed using PHYML version 3.1 with statistical tests for branch support. The values indicate the aLRT scores.

Fig. S9. The multiple sequence alignment of Cas3 and Cse1 protein by Clustal Omega. a. Cas3 The domains were predicted using InterPro (https://www.ebi.ac.uk/interpro/). The alignment was done using NCBI BLAST. The similarity between the three domains- HD domain, DEAD box and Helicase C-terminal are indicated by yellow, green and blue boxes respectively. **b. Cse1** The bold red color represents fully conserved residues (*) and strongly similar groups with a score >0.5 in Gonnet PAM 250 matrix (:) and the red color represent groups of weekly similar properties with a score <0.5 (.) for the residues essential for PAM recognition. The residues essential for Cas3 protein recruitment is indicated by the red box

Fig. S10. The phylogeny of CRISPR1 leader. The CRISPR1 leader sequences of 128 strains were aligned using MUSCLE and the phylogenetic trees was constructed by Maximum Likelihood.

Fig. S11. Heat map for sharing of protospacer source by pairs of serovars for spacers belonging to a. CRISPR1 array and **b. CRISPR2 array.** The protospacer sources - phages, plasmids and DNA viruses were extracted using the tool CRISPR target. The protospacers were matched among all serovars and a map of same targets were created. The heat maps were created using the tool Seaborn version 0.11 available at https://seaborn.pydata.org/[1].

Fig. S12. Graph representing the relationship between the no. of spacers and no. of protospacer for a. CRISPR1 array and b. CRISPR2 array. The X-axis values is in the increasing number of spacers. Y-axis represent the values of obtained protospacer matches corresponding to all the spacers of the array. The graphs were created using GraphPad Prism 8 available at https://www.graphpad.com/

Fig. S13. Sequence alignment of the region between the transposase and CRISPR1 in *S. enterica* subsp. *enterica* serovar Typhimurium and *Citrobacter freundii* complex sp. CFNIH3. The region between the transposase and CRISPR1 was extracted from NCBI database. The sequences were then aligned using NCBI nucleotide BLAST. 81.98% identity was obtained for 51% query cover yielding nearly 40% sequence similarity.

Figure S1

a.

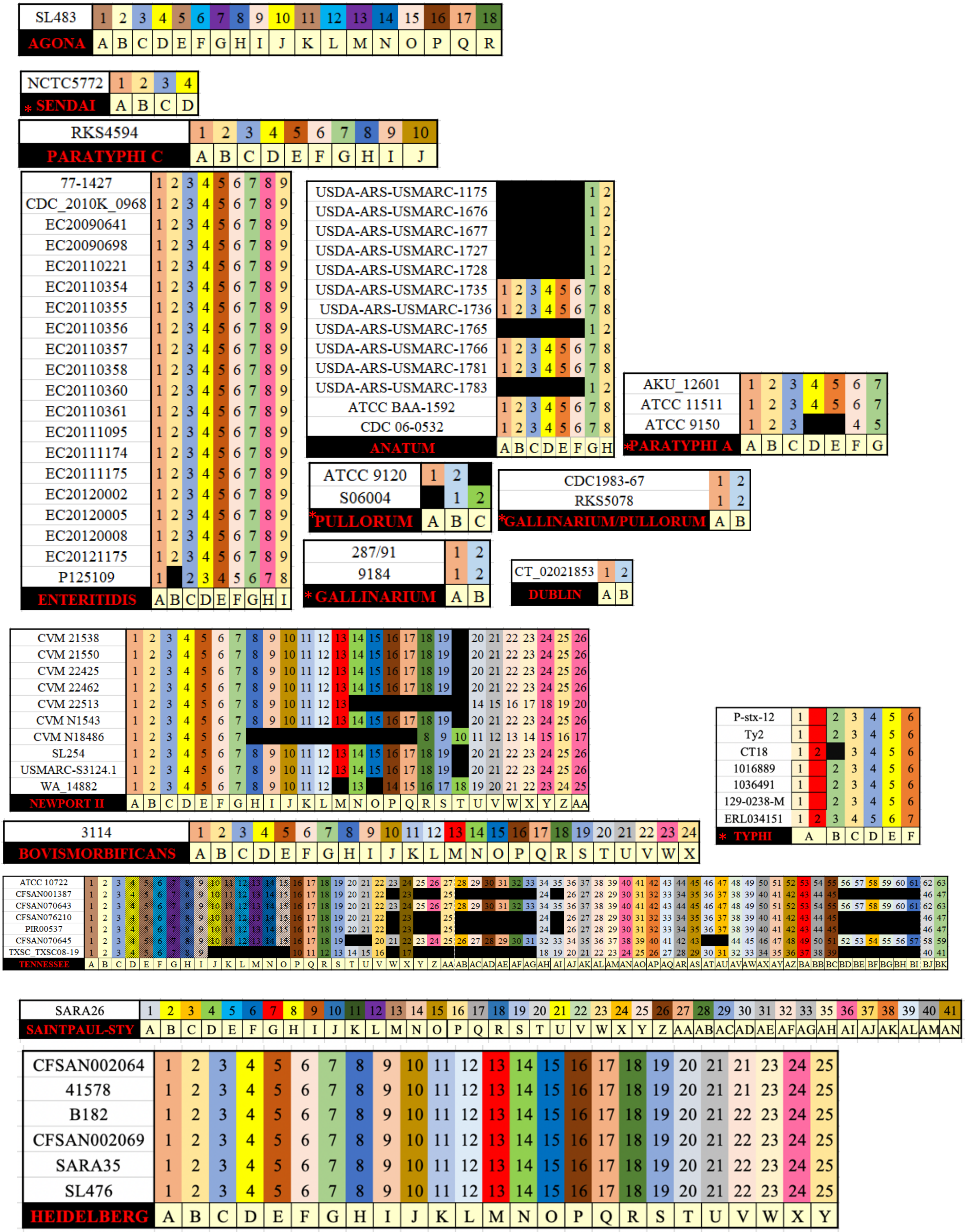
SL483
AGONA
NCTC5772
* SENDAI
RKS4594
PARATYPHI C
77-1427
CDC_2010K_0968
EC20090641
EC20090698
EC20110221
EC20110354
EC20110355
EC20110356
EC20110357
EC20110358
EC20110360
EC20110361
EC20111095
EC20111174
EC20111175
EC20120002
EC20120005
EC20120008
EC20121175
P125109
ENTERITIDIS
USDA-ARS-USMARC-1175
USDA-ARS-USMARC-1676
USDA-ARS-USMARC-1677
USDA-ARS-USMARC-1727
USDA-ARS-USMARC-1728
USDA-ARS-USMARC-1735
USDA-ARS-USMARC-1736
USDA-ARS-USMARC-1765
USDA-ARS-USMARC-1766
USDA-ARS-USMARC-1781
USDA-ARS-USMARC-1783
ATCC BAA-1592
CDC 06-0532
ANATUM
AKU_12601
ATCC 11511
ATCC 9150
*PARATYPHI A
ATCC 9120
S06004
*PULLORUM
CDC1983-67
RKS5078
*GALLINARIUM/PULLORUM
287/91
9184
* GALLINARIUM
CT_02021853
DUBLIN
CVM 21538
CVM 21550
CVM 22425
CVM 22462
CVM 22513
CVM N1543
CVM N18486
SL254
USMARC-S3124.1
WA_14882
NEWPORT II
P-stx-12
Ty2
CT18
1016889
1036491
129-0238-M
ERL034151
* TYPHI
3114
BOVISMORBIFICANS
ATCC 10722
CFSAN001387
CFSAN070643
CFSAN076210
PIR00537
CFSAN070645
TXSC_TXSC08-19
TENNESSEE
SARA26
SAINTPAUL-STY
CFSAN002064
41578
B182
CFSAN002069
SARA35
SL476
HEIDELBERG

USDA-ARS-USMARC-1808		1																			2	3	4	5	6	7	8	9	10
USDA-ARS-USMARC-1810		1						2			3	4	5	6	7	8	9	10	11	12	13	14	15	16	17	18	19	20	21
USDA-ARS-USMARC-1880		1	2															3	4	5	6	7	8				9	10	11
USDA-ARS-USMARC-1896	1	2	3	4	5	6	7	8	9	10	11	12	13	14	15	16		17	18	19	20	21	22	23	24	25	26	27	28
USDA-ARS-USMARC-1898		1	2	3		4	5	6	7	8	9	10	11	12		13	14		15	16	17	18	19				20	21	22
USDA-ARS-USMARC-1899		1					2	3			4	5	6															7	8
str. 14028S		1					2	3			4	5	6	7	8	9	10	11	12	13	14	15	16	17	18	19	20	21	22
str. 798		1					2	3			4	5	6	7	8	9	10	11	12	13	14	15	16	17	18	19	20	21	22
CDC 2009K-1640		1																			2	3	4	5	6	7	8	9	10
CDC 2009K-2059	1	2	3	4	5	6	7	8	9	10	11	12	13	14	15	16		17	18	19	20	21	22	23	24	25	26	27	28
CDC 2010K-1587		1	2	3	4		5	6	7	8	9	10	11	12	13	14	15	16	17	18	19	20	21	22	23	24	25	26	27
CDC 2011K-0870		1	2															3	4	5	6	7	8				9	10	11
CDC 2011K-1702		1																			2	3	4	5	6	7	8	9	10
D23580		1					2	3			4	5	6	7	8	9	10	11	12	13	14		15	16	17	18	19	20	21
DT104		1																			2	3	4	5	6	7	8	9	10
DT2		1					2	3			4	5	6	7	8	9	10	11	12		13	14	15	16	17	18		19	20
L-3553		1					2	3			4	5	6	7	8	9	10	11	12	13	14	15	16	17	18	19	20	21	22
SARA13		1					2	3			4	5	6	7						8	9	10	11	12	13	14	15	16	17
SL1344		1					2	3			4	5	6	7						8				9	10	11	12	13	14
ST4/74		1					2	3			4	5	6	7						8				9	10	11	12	13	14
T000240		1	2	3				4		5	6	7	8	9		10	11	12	13	14	15	16	17				18	19	20
UK-1		1					2	3			4	5	6	7			8	9	10	11	12	13	14						
CFSAN001921		1	2	3	4		5	6	7	8	9	10	11	12	13	14	15	16	17	18	19	20	21	22	23	24	25	26	27
TYPHIMURIUM	A	B	C	D	E	F	G	H	I	J	K	L	M	N	O	P	Q	R	S	T	U	V	W	X	Y	Z	AA	AB	AC

CFSAN001660	1	2	3	4	5	6	7				8	9	10	11	12	13	14	15
CDC 2009K-1331	1	2	3						4	5	6	7	8	9	10	11	12	13
CDC 2012K-0938	1															2	3	4
Levine 1	1	2	3						4	5	6	7	8	9	10	11	12	13
USDA-ARS-USMARC-1927	1	2	3	4	5	6	7	8	9	10	11	12	13	14	15	16	17	18
Levine 15	1														2	3	4	5
NEWPORT III	A	B	C	D	E	F	G	H	I	J	K	L	M	N	O	P	Q	R

CFSAN000510	1	2	3	4	5	6	7	8	9	10	11	12	13	14	15	16	17	18	19	20
NCTC12419	1	2	3	4	5	6	7	8	9	10	11	12	13	14	15	16	17	18	19	20
66 SA19983605	1	2	3	4	5	6	7	8	9	10	11	12	13	14	15	16	17	18	19	20
BONGORI	A	B	C	D	E	F	G	H	I	J	K	L	M	N	O	P	Q	R	S	T

50 MZ0080	1	2	3	4	5	6	7	8	9	10	11	12	13	14	15	16	17	18	19	20	21	22	23	24	25	26	27	28	29
DIARIZONAE	A	B	C	D	E	F	G	H	I	J	K	L	M	N	O	P	Q	R	S	T	U	V	W	X	Y	Z	AA	AB	AC

NCTC10047	1	2	3	4	5	6	7	8	9	10	11	12	13	14	15	16	17	18	19	20	21	22	23
ARIZONAE	A	B	C	D	E	F	G	H	I	J	K	L	M	N	O	P	Q	R	S	T	U	V	W

CFSAN004173	1	2	3	4	5	6	7	8	9	10	11	12	13	14	15
CFSAN004174	1	2	3	4	5	6	7	8	9	10	11	12	13	14	15
CFSAN004175	1	2	3	4	5	6	7	8	9	10	11	12	13	14	15
SGB23	1	2	3	4	5	6					7	8	9	10	11
SAINTPAUL-STM	A	B	C	D	E	F	G	H	I	J	K	L	M	N	O

FCC0123		1	2	3	4	5	6	7	8	9	10	11	12	13	14	15	16	17	18	19	20	21	22
CDC 2009K-0792				1	2	3	4	5	6	7	8	9	10	11	12	13	14	15	16	17	18	19	20
USDA-ARS-USMARC-1900		1	2	3	4	5	6	7	8	9	10	11	12	13	14	15	16	17	18	19	20	21	22
USDA-ARS-USMARC-1901				1	2	3	4	5	6	7	8	9	10	11	12	13	14	15	16	17	18	19	20
USDA-ARS-USMARC-1904				1	2	3	4	5	6	7	8	9	10	11	12	13	14	15	16	17	18	19	20
USDA-ARS-USMARC-1912				1	2	3	4	5	6	7	8	9	10	11					12	13	14	15	16
USDA-ARS-USMARC-1903	1	2	3	4	5	6	7	8	9	10	11	12	13	14	15	16	17	18	19	20	21	22	23
USDA-ARS-USMARC-1921	1	2	3	4	5	6	7	8	9	10	11	12	13	14	15	16	17	18	19	20	21	22	23
MONTEVIDEO-STY	A	B	C	D	E	F	G	H	I	J	K	L	M	N	O	P	Q	R	S	T	U	V	W

CFSAN051296	1	2	3	4	5						6		7	8	9	10				11	12	13		14	15	16	17
531954	1	2	3	4	5						6		7	8	9	10				11	12	13		14	15	16	17
507440-20	1	2	3	4	5						6		7	8	9	10				11	12	13		14	15	16	17
CDC 07-0954	1	2	3	4	5	6			7	8	9	10	11	12	13	14	15	16	17	18	19	20	21	22	23	24	25
CDC 08-1942	1	2	3	4	5	6	7	8	9	10	11	12	13	14	15						16	17		18	19	20	21
CDC 2010K-0257	1	2	3	4	5						6		7	8	9	10				11	12	13		14	15	16	17
CDC 2011K-1674	1	2	3	4	5	6		7	8	9			10	11	12	13			14	15	16	17		18	19	20	21
CDC 2012K-1544	1	2	3	4	5						6		7	8	9	10				11	12	13		14	15	16	17
CDC 2013K-0218	1	2	3	4	5	6	7	8	9	10	11	12	13	14	15						16	17		18	19	20	21
MONTEVIDEO-STM	A	B	C	D	E	F	G	H	I	J	K	L	M	N	O	P	Q	R	S	T	U	V	W	X	Y	Z	AA

b.

USDA-ARS-USMARC-1175	1	2	3	4	5	6	7	8	9	10	11	12	13	14	15	16	17	18	19	20	21	22	23	24	25	26
USDA-ARS-USMARC-1676	1	2								3	4	5	6	7	8	9	10	11	12	13	14	15	16	17	18	19
USDA-ARS-USMARC-1677						1	2	3	4	5	6	7	8	9	10	11	12	13	14	15	16	17	18	19	20	21
USDA-ARS-USMARC-1727	1	2	3															4	5	6	7	8	9	10	11	12
USDA-ARS-USMARC-1728	1	2	3	4	5	6	7	8	9		10	11	12	13	14	15	16	17	18	19	20	21	22	23	24	25
USDA-ARS-USMARC-1735																		1	2		3	4	5	6	7	8
USDA-ARS-USMARC-1736	1	2	3	4	5	6	7	8	9	10	11	12	13	14	15	16	17	18	19	20	21	22	23	24	25	26
USDA-ARS-USMARC-1765	1	2	3	4	5	6			7	8	9	10	11				12	13	14	15	16	17	18	19	20	21
USDA-ARS-USMARC-1766	1	2	3	4	5	6	7	8	9	10	11	12	13	14	15	16	17	18	19	20	21	22	23	24		
USDA-ARS-USMARC-1781	1	2	3	4	5	6	7	8	9	10							11	12	13	14	15	16	17	18	19	20
USDA-ARS-USMARC-1783	1	2	3	4	5	6	7	8	9		10	11	12	13	14	15	16	17	18	19	20	21	22	23	24	25
ATCC BAA-1592	1	2	3	4	5	6	7	8	9	10							11	12	13	14	15	16	17	18	19	20
CDC 06-0532	1	2	3	4	5	6	7	8	9	10							11	12	13	14	15	16	17	18	19	20
ANATUM	A	B	C	D	E	F	G	H	I	J	K	L	M	N	O	P	Q	R	S	T	U	V	W	X	Y	Z

77-1427	1					2	3	4	5	6	7	8
CDC_2010K_0968	1	2	3		4	5	6	7	8	9	10	11
EC20090641	1	2	3		4	5	6	7	8	9	10	11
EC20090698	1	2	3		4	5	6	7	8	9	10	11
EC20110221	1	2	3		4	5	6	7	8	9	10	11
EC20110354	1	2	3	4	5	6	7	8	9	10	11	12
EC20110355	1	2	3	4	5	6	7	8	9	10	11	12
EC20110356	1	2	3	4	5	6	7	8	9	10	11	12
EC20110357	1	2	3		4	5	6	7	8	9	10	11
EC20110358	1	2	3		4	5	6	7	8	9	10	11
EC20110360	1	2	3	4	5	6	7	8	9	10	11	12
EC20110361	1	2	3	4	5	6	7	8	9	10	11	12
EC20111095	1	2	3	4	5	6	7	8	9	10	11	12
EC20111174	1	2	3	4	5	6	7	8	9	10	11	12
EC20111175	1	2	3	4	5	6	7	8	9	10	11	12
EC20120002	1	2	3	4	5	6	7	8	9	10	11	12
EC20120005	1	2	3	4	5	6	7	8	9	10	11	12
EC20120008	1	2	3		4	5	6	7	8	9	10	11
EC20121175	1	2	3	4	5	6	7	8	9	10	11	12
P125109	1	2			3	4	5	6	7	8	9	10
ENTERITIDIS	A	B	C	D	E	F	G	H	I	J	K	L

CFSAN002064	1	2	3	4	5	6	7	8	9	10	11	12	13	14	15	16	17	18
41578	1	2	3	4	5			6	7	8	9	10	11	12	13	14	15	16
B182	1	2	3	4	5	6	7	8	9	10	11	12	13	14	15	16	17	18
CFSAN002069	1	2	3	4	5	6	7	8	9	10	11	12	13	14	15	16	17	18
SARA35	1	2	3	4	5	6	7	8	9	10	11	12	13	14	15	16	17	18
SL476	1	2	3	4	5	6	7	8	9	10	11	12	13	14	15	16	17	18
HEIDELBERG	A	B	C	D	E	F	G	H	I	J	K	L	M	N	O	P	Q	R

CVM 21538	1	2	3	4	5	6	7	8	9	10	11	12	13	14			15	16	17	18	19
CVM 21550	1	2	3	4	5	6	7	8	9	10	11	12	13	14			15	16	17	18	19
CVM 22425	1	2	3	4	5	6	7	8	9	10	11	12	13	14			15	16	17	18	19
CVM 22462	1	2	3	4	5	6	7	8	9	10	11	12	13	14			15	16	17	18	19
CVM 22513	1	2	3	4	5	6	7	8	9	10	11	12	13	14			15	16	17	18	19
CVM N1543	1	2	3	4	5	6	7	8	9	10	11	12	13	14			15	16	17	18	19
CVM N18486	1	2	3	4	5	6	7	8										9	10	11	12
SL254	1	2	3	4	5	6	7	8	9	10	11	12	13	14			15	16	17	18	19
USMARC-S3124.1	1	2	3	4	5	6	7	8	9	10	11	12	13	14			15	16	17	18	19
WA_14882	1	2	3	4	5	6	7	8			9	10	11	12	13	14	15	16	17	18	19
NEWPORT II	A	B	C	D	E	F	G	H	I	J	K	L	M	N	O	P	Q	R	S	T	U

NCTC5772	1
*SENDAI	A

287/91	1	2	3	4	5	6	7	8	9	10
9184	1	2	3	4	5	6	7	8	9	10
*GALLINARIUM	A	B	C	D	E	F	G	H	I	J

ATCC 9120	1	2	3	4	5	6
S06004	1					2
*PULLORUM	A	B	C	D	E	F

CT_02021853	1	2	3	4	5
DUBLIN	A	B	C	D	E

CDC1983-67	1	2	3	4	5	6
RKS5078	1	2	3	4	5	6
* GALLINARIUM/PULLORUM	A	B	C	D	E	F

AKU_12601	1	2	3
ATCC 11511	1	2	3
ATCC 9150	1	2	3
*PARATYPHI A	A	B	C

CFSAN004173	1	2	3	4	5	6	7	8	9	10			11	12	13	14	15	16	17	18	19	20
CFSAN004174	1	2	3	4	5	6	7	8	9	10			11	12	13	14	15	16	17	18	19	20
CFSAN004175	1	2	3	4	5	6	7	8	9	10			11	12	13	14	15	16	17	18	19	20
SGB23	1	2	3	4	5	6	7	8	9	10	11	12	13	14	15	16	17	16	19	20	21	22
SAINTPAUL-STM	A	B	C	D	E	F	G	H	I	J	K	L	M	N	O	P	Q	R	S	T	U	V

SARA26	1	2	3	4	5	6	7	8	9	10	11	12	13	14	15	16	17	18	19	20	21	22
SAINTPAUL-STY	A	B	C	D	E	F	G	H	I	J	K	L	M	N	O	P	Q	R	S	T	U	V

P-stx-12	1
Ty2	1
CT18	1
1016889	1
1036491	1
129-0238-M	1
ERL034151	1
*TYPHI	A

USDA-ARS-USMARC-1808	1	2	3	4	5			6	7	8	9	10	11	12	13			14	15	16	17	18	19	20	21	22	23								24	25	26
USDA-ARS-USMARC-1810	1	2	3				4	5		6	7	8	9	10	11			12	13	14	15	16	17	18	19	20	21	22	23	24	25	26	27	28	29	30	31
USDA-ARS-USMARC-1880	1	2	3	4	5	6	7	8	9	10	11	12	13	14	15			16	17	18	19	20	21	22	23	24	25	26	27	28	29	30	31	32	33	34	35
USDA-ARS-USMARC-1896													1	2	3	4	5	6	7	8	9	10		11	12	13	14	15	16	17	18	19	20	21	22	23	24
USDA-ARS-USMARC-1898		1	2	3	4	5	6	7	8	9	10	11	12	13	14			15		16	17	18		19	20	21	22	23	24	25	26	27	28	29	30	31	32
USDA-ARS-USMARC-1899	1	2	3				4	5	6	7	8	9																10	11	12	13	14	15	16	17	18	19
str. 14028S	1	2										3	4	5	6			7	8	9	10	11	12	13	14	15	16	17	18	19	20	21	22	23	24	25	26
str. 798			1						2		3	4	5	6	7			8	9	10	11	12	13	14	15	16	17	18	19						20	21	22
CDC 2009K-1640	1	2	3	4	5			6	7	8	9	10	11	12	13			14	15	16	17	18	19	20	21	22	23								24	25	26
CDC 2009K-2059													1	2	3	4	5	6	7	8	9	10		11	12	13	14	15	16	17	18	19	20	21	22	23	24
CDC 2010K-1587													1	2	3	4	5	6	7	8	9	10		11	12	13	14	15	16	17	18	19	20	21	22	23	24
CDC 2011K-0870	1	2	3	4	5	6	7	8	9	10	11	12	13	14	15			16		17	18	19	20	21	22	23	24	25	26	27	28	29	30	31	32	33	34
CDC 2011K-1702	1	2	3	4	5			6	7	8	9	10	11	12	13			14	15	16	17	18	19	20	21	22	23								24	25	26
D23580			1				2	3	4	5	6	7	8	9															10	11	12	13	14	15	16	17	18
DT104	1	2	3	4	5			6	7	8	9	10	11	12	13			14	15	16	17	18	19	20	21	22	23								24	25	26
DT2	1	2	3				4	5	6	7								8	9	10	11	12		13	14	15	16	17	18	19	20	21	22	23	24	25	26
L-3553	1	2	3				4	5		6	7	8	9	10	11			12																	13	14	15
SARA13	1										2	3	4	5	6			7	8	9	10	11	12	13		14	15	16	17	18	19	20	21	22	23	24	25
SL1344			1						2	3	4	5	6	7	8			9	10	11	12	13	14	15	16	17	18	19	20						21	22	23
ST4/74			1						2	3	4	5	6	7	8			9	10	11	12	13	14	15	16	17	18	19	20						21	22	23
T000240		1	2	3	4	5	6	7	8	9	10	11		12	13			14	15	16	17	18		19	20	21	22	23	24	25	26	27	28	29	30	31	32
UK-1	1	2										3	4	5	6			7	8	9	10				11	12	13	14	15	16	17	18	19	20	21	22	23
CFSAN001921													1	2	3	4	5	6	7	8	9	10		11	12	13	14	15	16	17	18	19	20	21	22	23	24
TYPHIMURIUM	A	B	C	D	E	F	G	H	I	J	K	L	M	N	O	P	Q	R	S	T	U	V	W	X	Y	Z	AA	AB	AC	AD	AE	AF	AG	AH	AI	AJ	AK

CFSAN001660	1	2	3	4	5	6	7	8	9	10	11	12	13	14	15		16	17	18	19	20
CDC 2009K-1331	1		2	3	4	5	6	7	8		9	10	11	12				13	14	15	16
CDC 2012K-0938	1	2	3	4	5	6	7	8	9	10	11	12	13	14	15		16	17	18	19	20
Levine 1	1		2	3	4	5	6	7	8		9	10	11	12	13	14	15	16	17	18	19
USDA-ARS-USMARC-1927	1	2												3	4	5	6	7	8	9	10
Levine 15	1	2	3	4	5	6				7	8	9	10	11			12	13	14	15	16
NEWPORT III	A	B	C	D	E	F	G	H	I	J	K	L	M	N	O	P	Q	R	S	T	U

3114	1	2	3	4	5	6	7	8	9	10	11	12	13	14	15
BOVISMORBIFICANS	A	B	C	D	E	F	G	H	I	J	K	L	M	N	O

SL483	1	2	3	4	5	6	7	8
AGONA	A	B	C	D	E	F	G	H

RKS4594	1	2	3	4	5	6	7	8	9
PARATYPHI C	A	B	C	D	E	F	G	H	I

ATCC 10722	1	2	3	4	5	6	7	8	9	10	11	12	13	14	15	16	17	18	19	20	21	22	23
CFSAN001387	1	2	3	4	5	6			7	8	9	10	11	12	13	14	15	16	17	18	19	20	21
CFSAN070643	1	2	3	4	5	6	7	8	9	10	11	12	13	14	15	16	17	18	19	20	21	22	23
CFSAN076210	1	2	3	4	5	6			7	8	9	10	11	12	13	14	15	16	17	18	19	20	21
PIR00537	1	2	3	4	5	6	7	8	9	10	11	12	13	14	15	16	17	18	19	20	21	22	23
CFSAN070645	1	2	3	4	5	6	7	8	9	10			11	12	13	14	15	16	17	18	19	20	21
TXSC_TXSC08-19	1	2	3	4	5	6	7	8	9	10	11	12	13	14	15	16	17	18	19	20	21	22	23
TENNESSEE	A	B	C	D	E	F	G	H	I	J	K	L	M	N	O	P	Q	R	S	T	U	V	W

CFSAN051296	1	2	3	4	5						6		7	8	9	10				11	12	13		14	15	16	17
531954	1	2	3	4	5						6		7	8	9	10				11	12	13		14	15	16	17
507440-20	1	2	3	4	5						6		7	8	9	10				11	12	13		14	15	16	17
CDC 07-0954	1	2	3	4	5	6			7	8	9	10	11	12	13	14	15	16	17	18	19	20	21	22	23	24	25
CDC 08-1942	1	2	3	4	5	6	7	8	9	10	11	12	13	14	15						16	17		18	19	20	21
CDC 2010K-0257	1	2	3	4	5						6		7	8	9	10				11	12	13		14	15	16	17
CDC 2011K-1674	1	2	3	4	5	6		7	8	9			10	11	12	13			14	15	16	17		18	19	20	21
CDC 2012K-1544	1	2	3	4	5						6		7	8	9	10				11	12	13		14	15	16	17
CDC 2013K-0218	1	2	3	4	5	6	7	8	9	10	11	12	13	14	15						16	17		18	19	20	21
MONTEVIDEO-STM	A	B	C	D	E	F	G	H	I	J	K	L	M	N	O	P	Q	R	S	T	U	V	W	X	Y	Z	AA

FCC0123		1	2	3	4	5	6	7	8	9	10	11	12	13	14	15	16	17	18	19	20	21	22
CDC 2009K-0792				1	2	3	4	5	6	7	8	9	10	11	12	13	14	15	16	17	18	19	20
USDA-ARS-USMARC-1900		1	2	3	4	5	6	7	8	9	10	11	12	13	14	15	16	17	18	19	20	21	22
USDA-ARS-USMARC-1901				1	2	3	4	5	6	7	8	9	10	11	12	13	14	15	16	17	18	19	20
USDA-ARS-USMARC-1904				1	2	3	4	5	6	7	8	9	10	11	12	13	14	15	16	17	18	19	20
USDA-ARS-USMARC-1912				1	2	3	4	5	6	7	8	9	10	11					12	13	14	15	16
USDA-ARS-USMARC-1903	1	2	3	4	5	6	7	8	9	10	11	12	13	14	15	16	17	18	19	20	21	22	23
USDA-ARS-USMARC-1921	1	2	3	4	5	6	7	8	9	10	11	12	13	14	15	16	17	18	19	20	21	22	23
MONTEVIDEO-STY	A	B	C	D	E	F	G	H	I	J	K	L	M	N	O	P	Q	R	S	T	U	V	W

CFSAN000510	1	2	3	4	5	6	7	8	9	10	11	12	13	14	15	16	17
NCTC12419	1	2	3	4	5	6	7	8	9	10	11	12	13	14	15	16	17
66 SA19983605	1	2	3	4	5	6	7	8	9	10	11	12	13	14	15	16	17
BONGORI	A	B	C	D	E	F	G	H	I	J	K	L	M	N	O	P	Q

c.

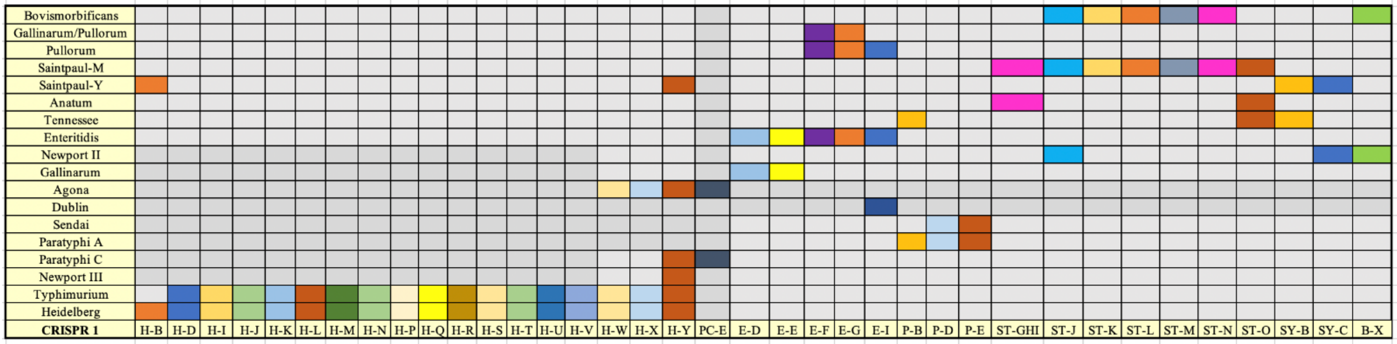
Bovismorbificans
Gallinarum/Pullorum
Pullorum
Saintpaul-M
Saintpaul-Y
Anatum
Tennessee
Enteritidis
Newport II
Gallinarum
Agona
Dublin
Sendai
Paratyphi A
Paratyphi C
Newport III
Typhimurium
Heidelberg
CRISPR 1
H-B H-D H-I H-J H-K H-L H-M H-N H-P H-Q H-R H-S H-T H-U H-V H-W H-X H-Y PC-E E-D E-E E-F E-G E-I P-B P-D P-E ST-GHI ST-J ST-K ST-L ST-M ST-N ST-O SY-B SY-C B-X

d.

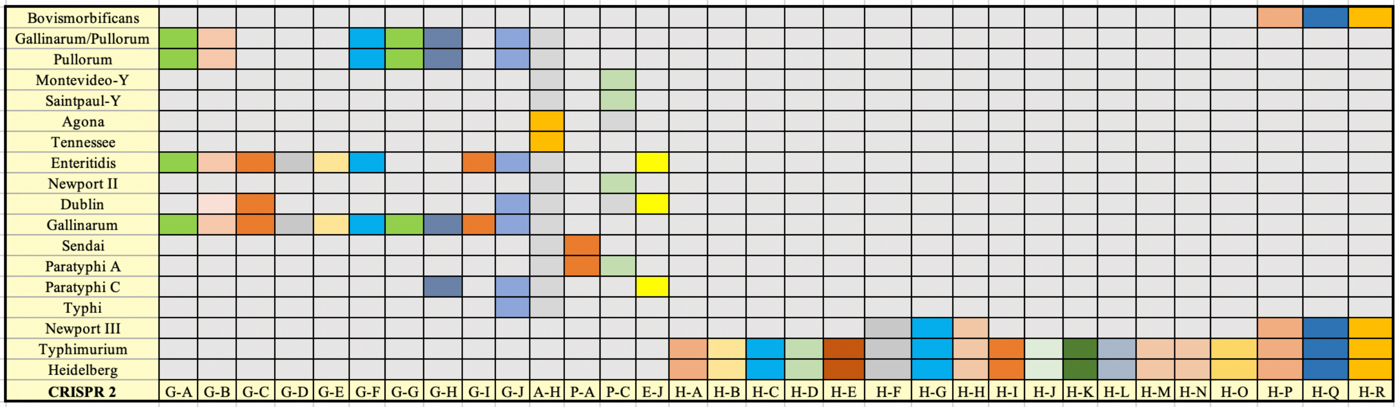
Bovismorbificans
Gallinarum/Pullorum
Pullorum
Montevideo-Y
Saintpaul-Y
Agona
Tennessee
Enteritidis
Newport II
Dublin
Gallinarum
Sendai
Paratyphi A
Paratyphi C
Typhi
Newport III
Typhimurium
Heidelberg
CRISPR 2
G-A G-B G-C G-D G-E G-F G-G G-H G-I G-J A-H P-A P-C E-J H-A H-B H-C H-D H-E H-F H-G H-H H-I H-J H-K H-L H-M H-N H-O H-P H-Q H-R

Figure S2

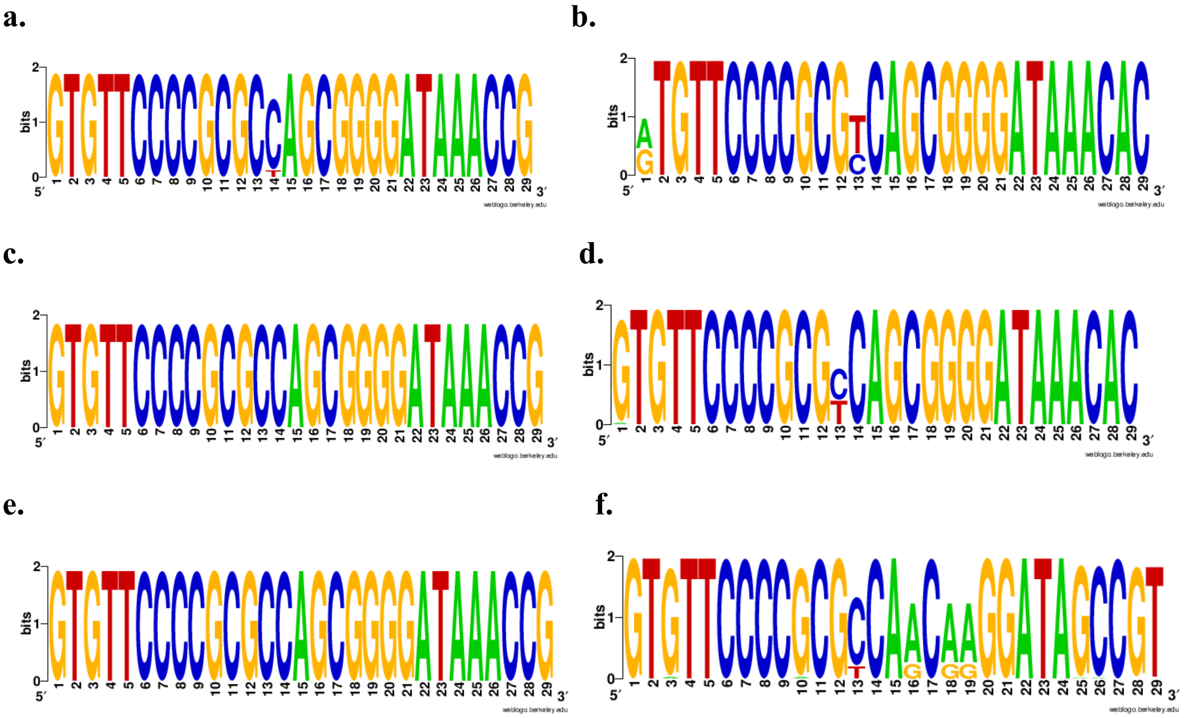
a.
b.
c.
d.
e.
f.
bits
5′
3′
weblogo.berkeley.edu

Figure S3

a.

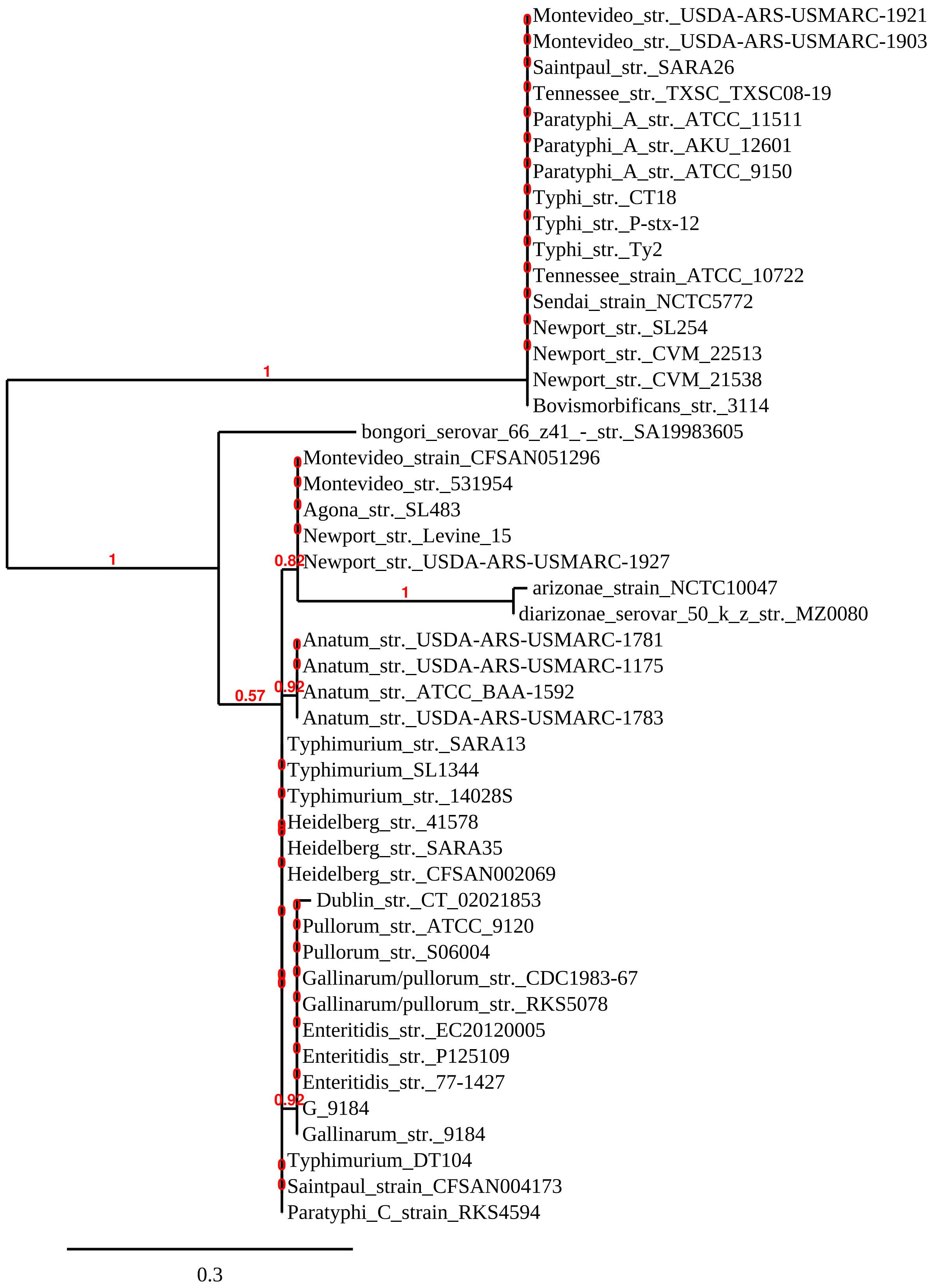
Montevideo_str._USDA-ARS-USMARC-1921
Montevideo_str._USDA-ARS-USMARC-1903
Saintpaul_str._SARA26
Tennessee_str._TXSC_TXSC08-19
Paratyphi_A_str._ATCC_11511
Paratyphi_A_str._AKU_12601
Paratyphi_A_str._ATCC_9150
Typhi_str._CT18
Typhi_str._P-stx-12
Typhi_str._Ty2
Tennessee_strain_ATCC_10722
Sendai_strain_NCTC5772
Newport_str._SL254
Newport_str._CVM_22513
Newport_str._CVM_21538
Bovismorbificans_str._3114
1
bongori_serovar_66_z41_-_str._SA19983605
Montevideo_strain_CFSAN051296
Montevideo_str._531954
Agona_str._SL483
Newport_str._Levine_15
Newport_str._USDA-ARS-USMARC-1927
0.82
1
1
arizonae_strain_NCTC10047
diarizonae_serovar_50_k_z_str._MZ0080
Anatum_str._USDA-ARS-USMARC-1781
Anatum_str._USDA-ARS-USMARC-1175
0.92
Anatum_str._ATCC_BAA-1592
0.57
Anatum_str._USDA-ARS-USMARC-1783
Typhimurium_str._SARA13
Typhimurium_SL1344
Typhimurium_str._14028S
Heidelberg_str._41578
Heidelberg_str._SARA35
Heidelberg_str._CFSAN002069
Dublin_str._CT_02021853
Pullorum_str._ATCC_9120
Pullorum_str._S06004
Gallinarum/pullorum_str._CDC1983-67
Gallinarum/pullorum_str._RKS5078
Enteritidis_str._EC20120005
Enteritidis_str._P125109
Enteritidis_str._77-1427
0.92
G_9184
Gallinarum_str._9184
Typhimurium_DT104
Saintpaul_strain_CFSAN004173
Paratyphi_C_strain_RKS4594
0.3

b.

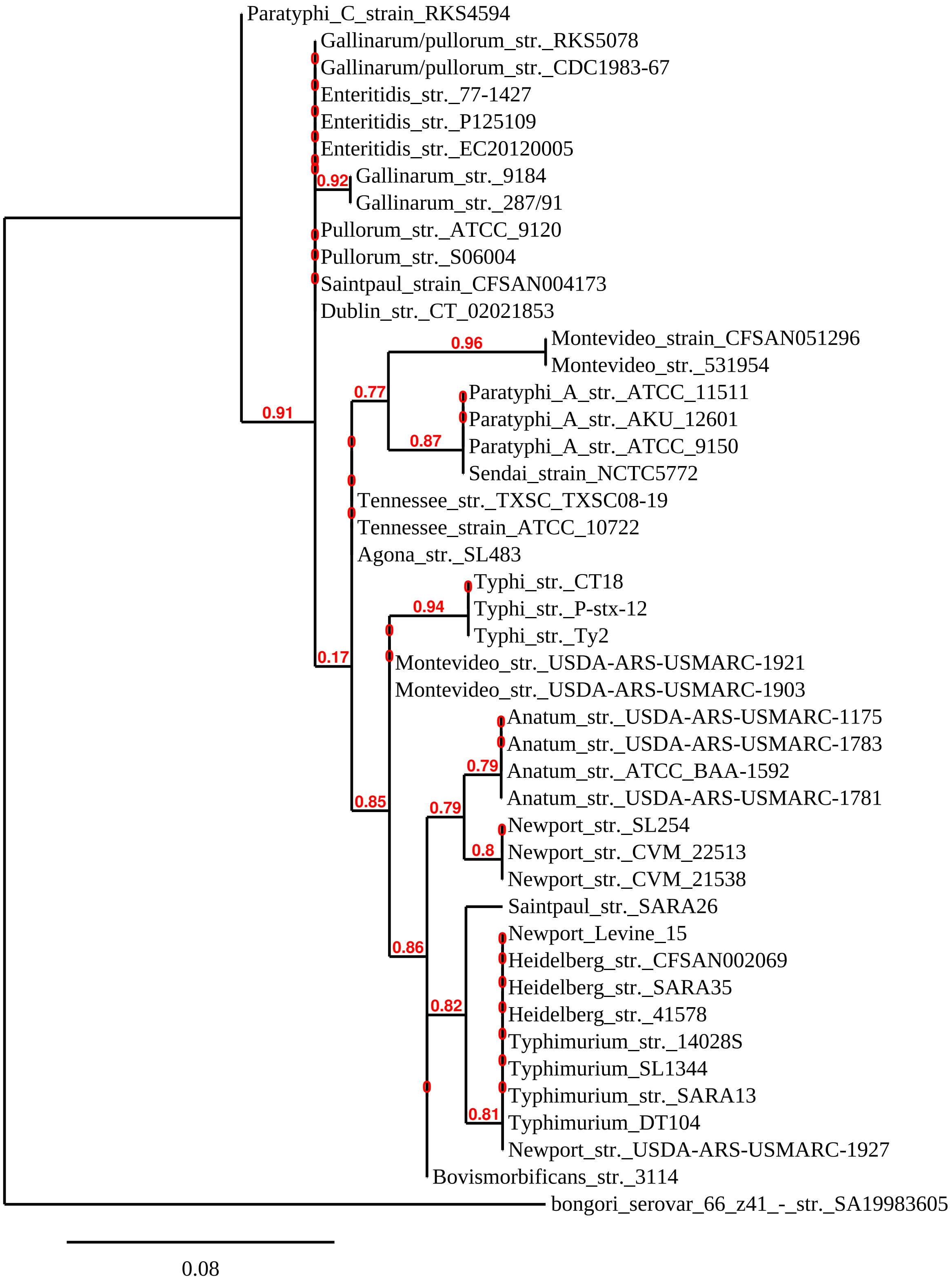
Paratyphi_C_strain_RKS4594
Gallinarum/pullorum_str._RKS5078
Gallinarum/pullorum_str._CDC1983-67
Enteritidis_str._77-1427
Enteritidis_str._P125109
Enteritidis_str._EC20120005
0.92
Gallinarum_str._9184
Gallinarum_str._287/91
Pullorum_str._ATCC_9120
Pullorum_str._S06004
Saintpaul_strain_CFSAN004173
Dublin_str._CT_02021853
0.96
Montevideo_strain_CFSAN051296
Montevideo_str._531954
0.77
Paratyphi_A_str._ATCC_11511
Paratyphi_A_str._AKU_12601
0.91
0.87
Paratyphi_A_str._ATCC_9150
Sendai_strain_NCTC5772
Tennessee_str._TXSC_TXSC08-19
Tennessee_strain_ATCC_10722
Agona_str._SL483
Typhi_str._CT18
0.94
Typhi_str._P-stx-12
Typhi_str._Ty2
0.17
Montevideo_str._USDA-ARS-USMARC-1921
Montevideo_str._USDA-ARS-USMARC-1903
Anatum_str._USDA-ARS-USMARC-1175
Anatum_str._USDA-ARS-USMARC-1783
0.79
Anatum_str._ATCC_BAA-1592
Anatum_str._USDA-ARS-USMARC-1781
0.85
0.79
Newport_str._SL254
0.8
Newport_str._CVM_22513
Newport_str._CVM_21538
Saintpaul_str._SARA26
Newport_Levine_15
0.86
Heidelberg_str._CFSAN002069
Heidelberg_str._SARA35
0.82
Heidelberg_str._41578
Typhimurium_str._14028S
Typhimurium_SL1344
Typhimurium_str._SARA13
0.81
Typhimurium_DT104
Newport_str._USDA-ARS-USMARC-1927
Bovismorbificans_str._3114
bongori_serovar_66_z41_-_str._SA19983605
0.08

Figure S4

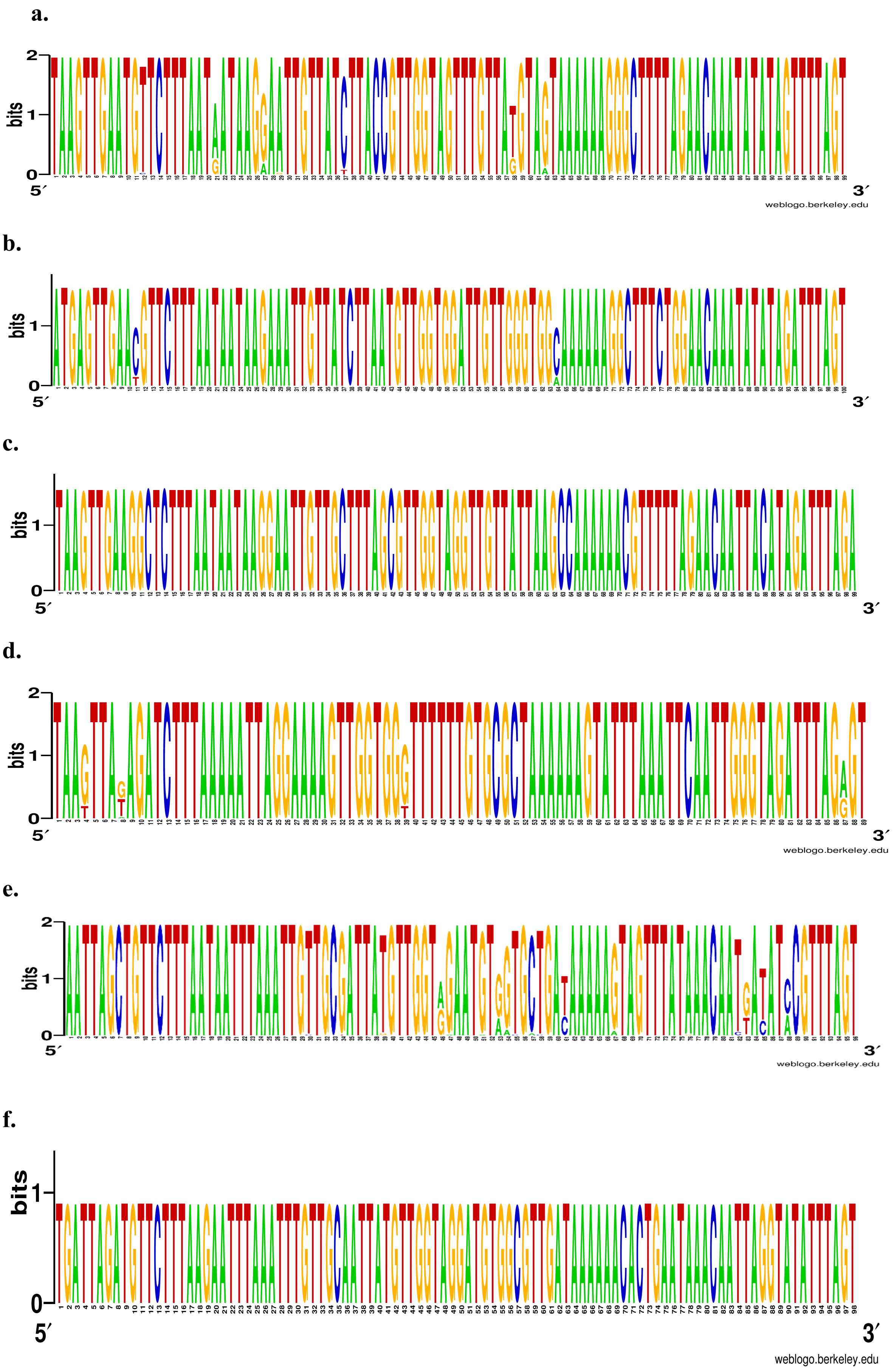

Figure S5

a.

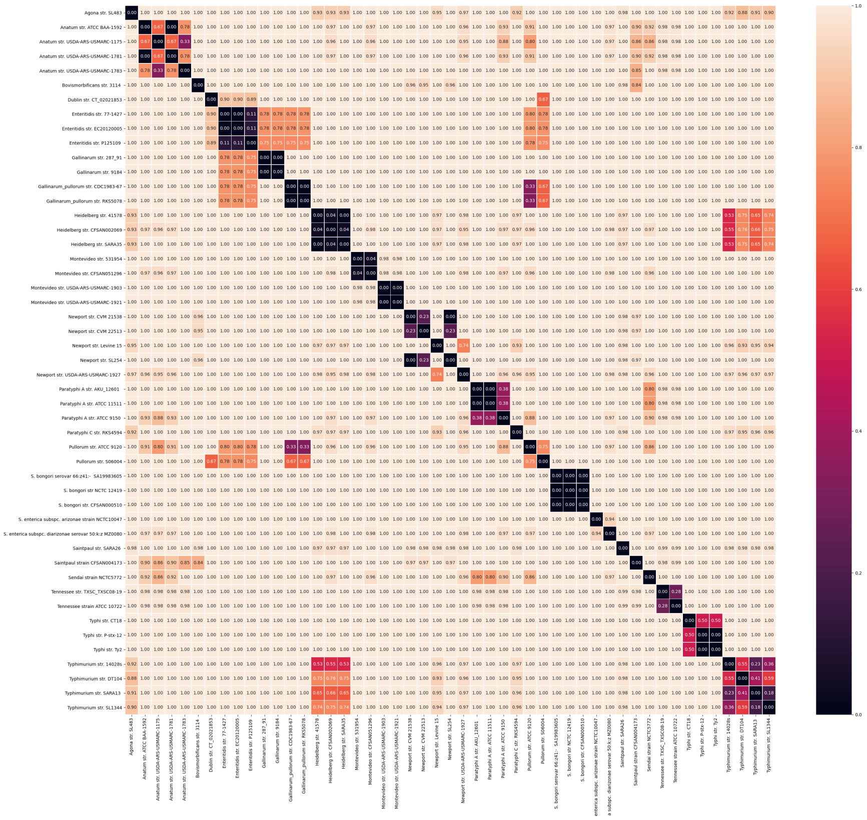

b.

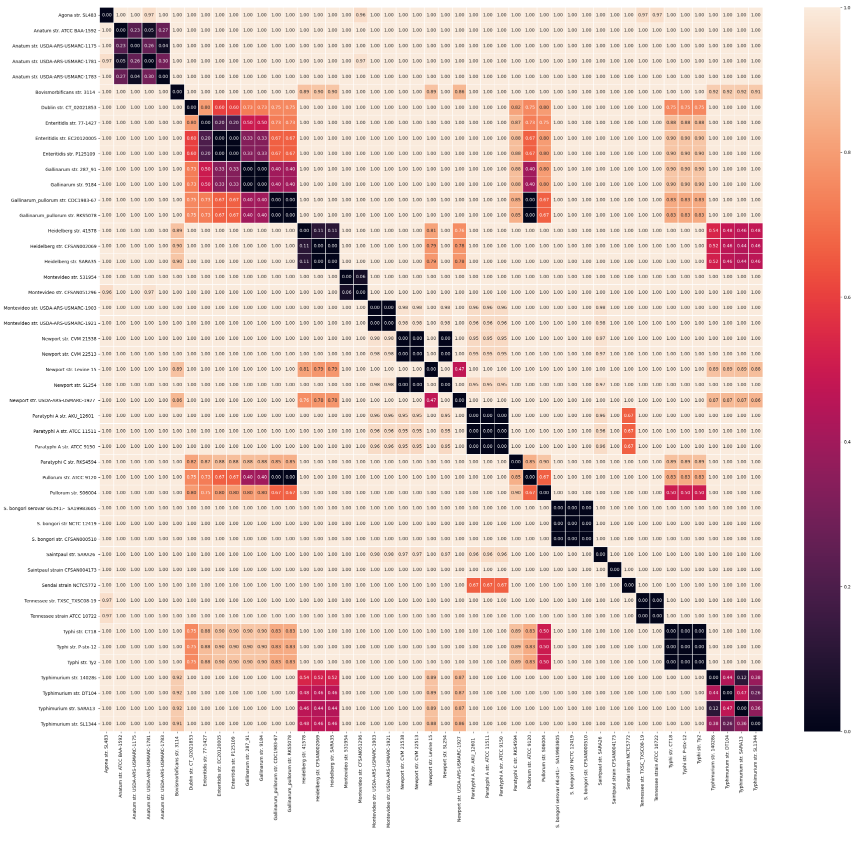

c.

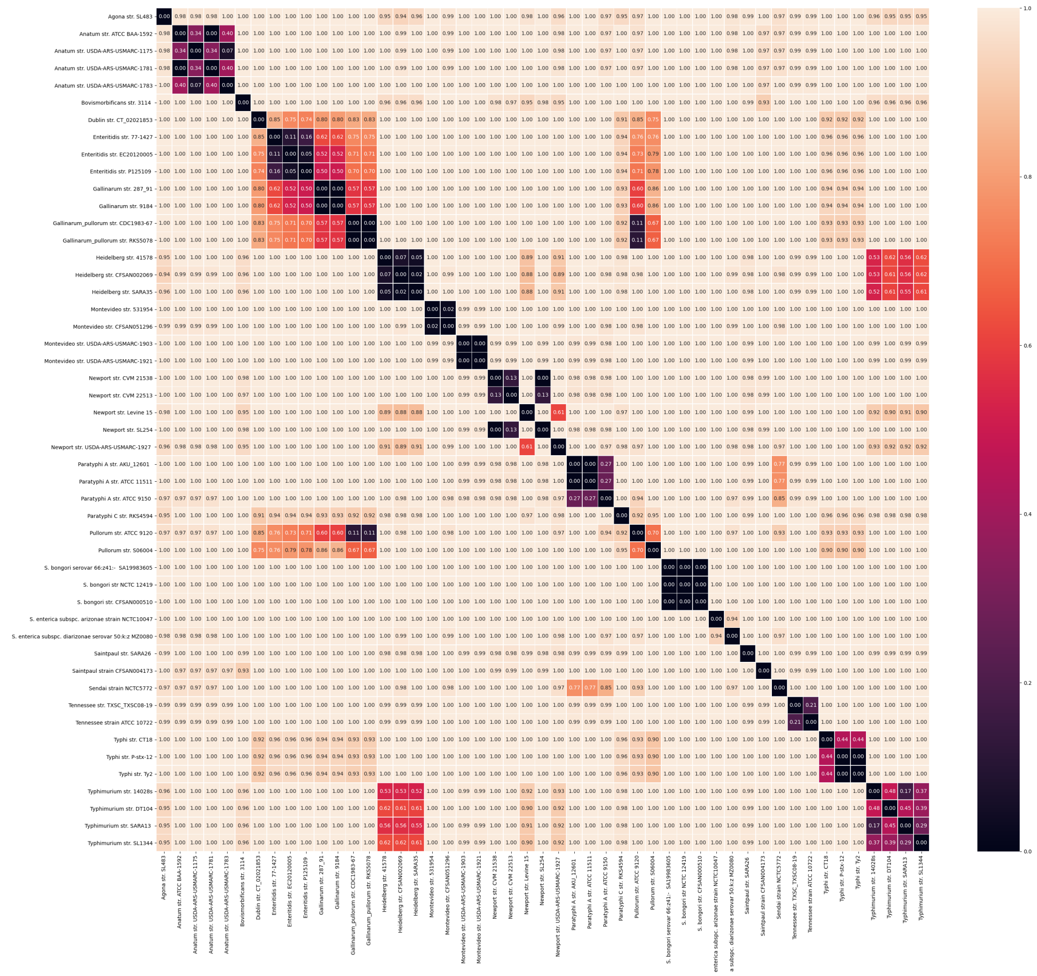

Figure S6

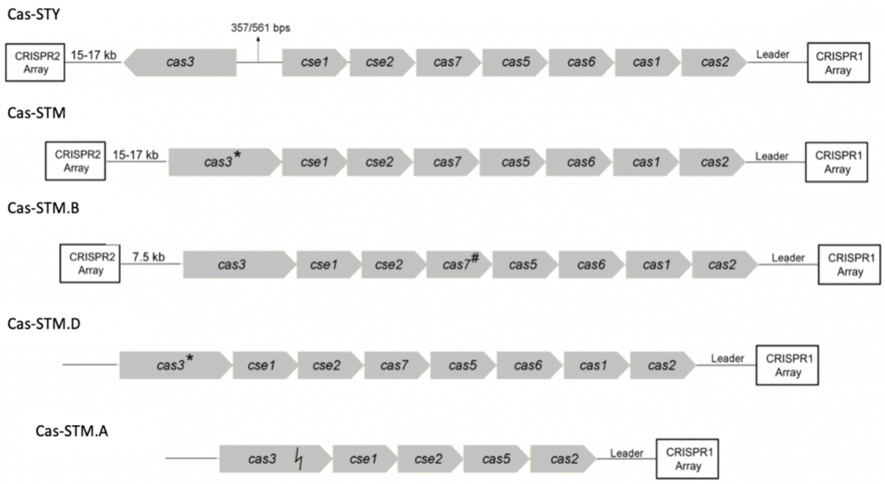

Figure S7

	Cas-STM	Cas-STY	Cas-STM.A	Cas-STM.D	Cas-STM.B
Cas-STM		28.6	87	87	85
Cas-STY			23.5	24	29
Cas-STM.A				99	88
Cas-STM.D					88.2
Cas-STM.B					

Figure S8

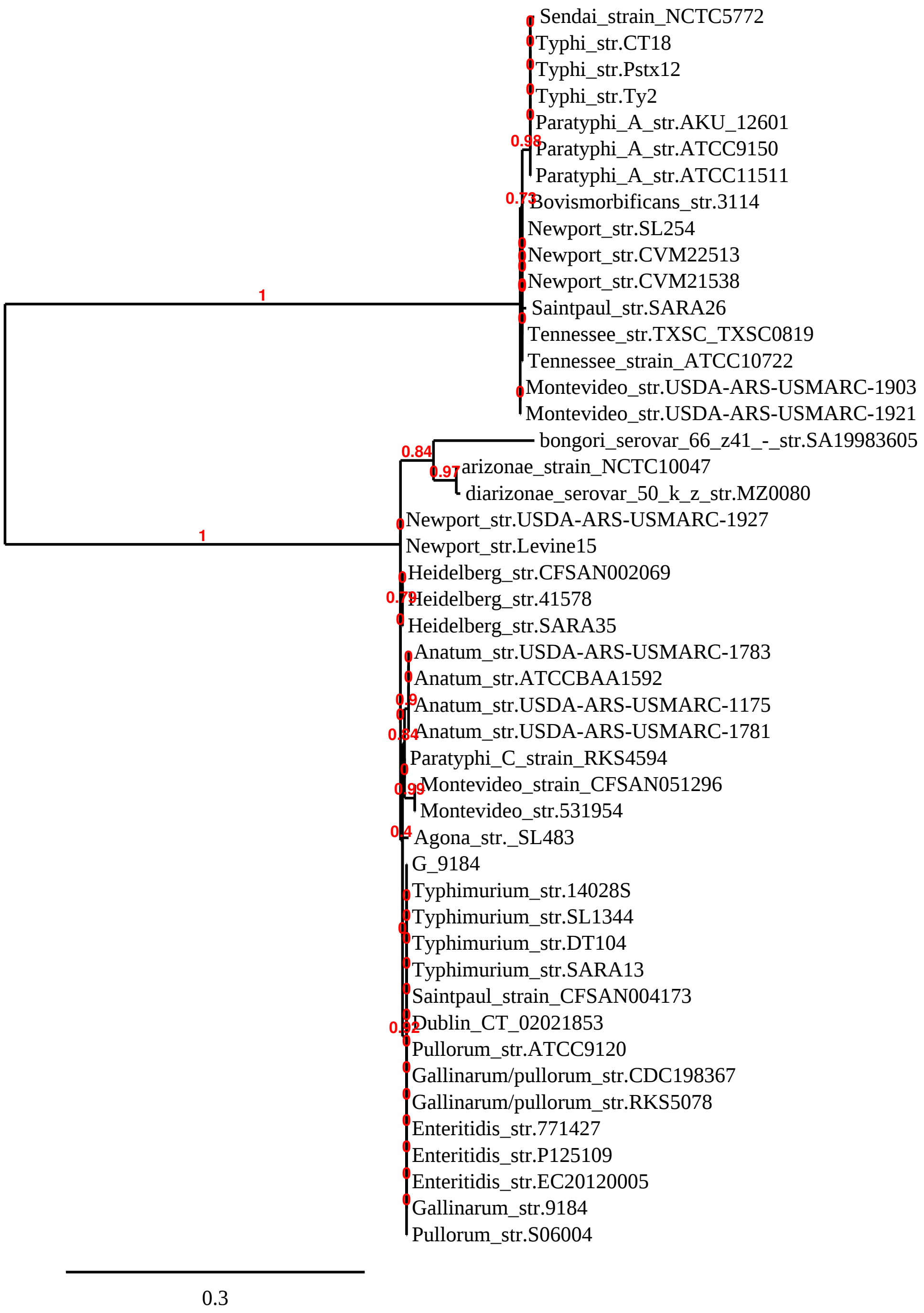
Sendai_strain_NCTC5772
Typhi_str.CT18
Typhi_str.Pstx12
Typhi_str.Ty2
Paratyphi_A_str.AKU_12601
0.98
Paratyphi_A_str.ATCC9150
Paratyphi_A_str.ATCC11511
0.73
Bovismorbificans_str.3114
Newport_str.SL254
Newport_str.CVM22513
Newport_str.CVM21538
1
Saintpaul_str.SARA26
Tennessee_str.TXSC_TXSC0819
Tennessee_strain_ATCC10722
Montevideo_str.USDA-ARS-USMARC-1903
Montevideo_str.USDA-ARS-USMARC-1921
bongori_serovar_66_z41_-_str.SA19983605
0.84
0.97
arizonae_strain_NCTC10047
diarizonae_serovar_50_k_z_str.MZ0080
Newport_str.USDA-ARS-USMARC-1927
1
Newport_str.Levine15
Heidelberg_str.CFSAN002069
Heidelberg_str.41578
Heidelberg_str.SARA35
Anatum_str.USDA-ARS-USMARC-1783
Anatum_str.ATCCBAA1592
Anatum_str.USDA-ARS-USMARC-1175
Anatum_str.USDA-ARS-USMARC-1781
Paratyphi_C_strain_RKS4594
Montevideo_strain_CFSAN051296
Montevideo_str.531954
Agona_str._SL483
G_9184
Typhimurium_str.14028S
Typhimurium_str.SL1344
Typhimurium_str.DT104
Typhimurium_str.SARA13
Saintpaul_strain_CFSAN004173
Dublin_CT_02021853
Pullorum_str.ATCC9120
Gallinarum/pullorum_str.CDC198367
Gallinarum/pullorum_str.RKS5078
Enteritidis_str.771427
Enteritidis_str.P125109
Enteritidis_str.EC20120005
Gallinarum_str.9184
Pullorum_str.S06004
0.3

Figure S9

a.

```
                 HD Domain - 48%
YWGKSRRGETDGGDDYHLLCWHSLDVAAVGYWMVINNIYFIDHYLKKLGIQDKEQAAQFF Typhimurium
YWGK+ +   +   +YHLL +H LDVAA G +++ NN +    + LK+  I     A  +
YWGKTDKSTENITCNYHLLAYHCLDVAACGEYIIKNNTFNSCNILKECNISGT-NAENWI Typhi

AWILCWHDIGKFAHSFQQLYRHEALNIFNEPTRHYEKIAHTTLGYMLWN----SWLSECP Typhimurium
 W L  HDIGKFA  FQ+        + +  +       H +LG+ LW     +W S
IWFLASHDIGKFARGFQKYAEFPDSPLVSPISGVSAFERHDSLGFYLWGKLFEAWSSGSN Typhi

ELFPPSSLSVR-KSKRVMALWMPVTTGHHGRPPEAIQELDHFRQQDKD--AARDFLLRIK Typhimurium
E+        R + +  +  WM ++TGHHG PP+ ++        D+D  AA  +L  +
EIIAGIEPENRARFESALNSWMLISTGHHGIPPDTMKNRSSLAFTDEDIVAATHYLEALS Typhi

ALFPLITLPEAWDEDEGIDQFQQLSWFISAAVVLADWTGSASRYFPRTAEKMPVDTYWQQ Typhimurium
 LFP  TLP+ W    G    +Q SWF +  V LADW GS    FP  +  MP+  YW
ELFPF-TLPQEWKTKAGRKCLKQHSWFFAGLVTLADWMGSDESQFPLLSSAMPLKDYWPL Typhi
                                  DEAD Box - 77%
ALAKAQTAITLFPSAANVSAFTGIETLFPFIQHPTPLQQKALELDINVDGAQLFILEDVT Typhimurium
A  KAQ AI   P  +  S +     LFPFIQ  TPLQQ+A ELDI+  GAQL +LEDVT
ACEKAQQAILRMPPLSQHSHYQDHRALFPFIQTLTPLQQRASELDISAPGAQLVVLEDVT Typhi

GAGKTEAALILAHRLMAAGKAQGLYFGLPTMATANAMFERMANTWLALYQPDSRPSLILA Typhimurium
GAGKTEAALIL HRLM+A K  GLY GLPTMATANAM++R+A+ + AL+  +SRPSLILA
GAGKTEAALILTHRLMSANKGHGLYVGLPTMATANAMYQRLASAYHALFTDESRPSLILA Typhi

HSARRLMDRFNQSIWSVTLSGTEE--PDEAQPYSQGCAAWFADSNKKALLAEVGVGTLDQ Typhimurium
H  R + D F QSIW  T +  E+   D+    ++ C  WFADS KKALLAEVGVGTLDQ
HGGREMSDSFRQSIWQPTENIAEDYARDDGNATTE-CHTWFADSRKKALLAEVGVGTLDQ Typhi

AMMAVMPFKHNNLRLLGLSNKILLADEIHACDAWMSRILEGLIERQASNGNATILLSATL Typhimurium
 +MAVMPF+H +LRLLG+ +KILL DE+HA D +M ++LEGL+   A+ G + I+LSATL
LLMAVMPFRHQSLRLLGMRDKILLLDEVHAYDGYMVKLLEGLLRFHAAQGGSAIILSATL Typhi

SQQQRDKLVAAFSRGVRRSVQAPLLGHDD--YPWLTQVTQTELISQRVDTRKEVERCVDI Typhimurium
    R+KL+ AFS G          G D+  YPWL+ +T + L+ Q + TR EV+R V +
PAALREKLLNAFSDGAEFMSAG---GSDNAGYPWLSHLTSSGLLEQPLATRPEVQRTVAV Typhi
                         Helicase C-terminal - 81%
GWLHSEEACLERIGEAVEKGNCIAWIRNSVDDAIRIYRQLQLSKVVVTENLLLFHSRFAF Typhimurium
 W+   +  L+ I    V  G CI WIRN+VDDA+  Y+QL    +V  ++LLLFHSRFAF
NWIQQRQEALDIIYRVVATGQCICWIRNTVDDALDTYQQLLHEGIVPEQDLLLFHSRFAF Typhi

YDRQRIESQTLNLFGKQSG-AQRAGKVIIATQVIEQSLDIDCDEMISDLAPVDLLIQRAG Typhimurium
 DR  IE++TLN FG  +  ++R GKV+IATQV+EQSLD+D D MI+DLAP+DLLIQRAG
IDRIAIENKTLNWFGNNAPVSERRGKVLIATQVVEQSLDLDFDWMITDLAPIDLLIQRAG Typhi

RLQRHIRDRNGLVKKSGQDERETPVLRILAPEWDDAPR                       Typhimurium
RLQRHIRD +G  K +   DER+ PVL I  P    A R
RLQRHIRDAHGQRKSTLPDERQPPVLHISGPSLAGAGR                       Typhi
```

b.

```
E.coli          ---MNLLIDNWIPVRPRNGGKVQIINLQSLYCSRDQWRLSLPRDDMELAALALLVCIGQI    57
Typhimurium     MDNFSLLTTPWLPVRFKDGSTGKLAPVDLA--DENVVDIAATRADLQGAAWQFLLGLLQC    58
Typhi           ---MDLTKEKWLPVIFSNGDKKKISLRDLL--DNRIQDLAYPRADFQGAAWQMLIGILQC    55
                   :.*    *:**   :*.. ::   :    ..    ::  * *:: **  :*: : *

E.coli          IAPAKDDVEFRHRIMNPLTEDEFQQLIAPWIDMFYLNHAEHPFMQTKGVKANDVTPMEKL    117
Typhimurium     SIAPKRYKNWEDIWFDGLHADVLHKALAPLEHAFQFGAETPSFMQDFEPLSGEKVSIASL    118
Typhi           TVAPEDKEEWADIWHESIEFEQWEKALNTISLALQFGEQKPSFLQSFDPLDSEYGSIAGL    115
                    :   :: .   : :  :  .: :      : :.     *:*      .:   :  *

E.coli          LAGVSGATNCA----FVNQPGQGEALCGGCTAIALFNQANQAPGFGGGFKSGLRGGTPVT    173
Typhimurium     LPEIPGAQTTKFNKDHFVKRGVTERFCPHCAALALFSLQLNAPAGGKGYRTGLRGGGPLT    178
Typhi           LVDAPGGNALKLNKDHFVKRGNVEQICPHCAAIALFAIQTNSPAGGAGYRVGMRGGGPLT    175
                *    *.        .. : *  * :*  *:*:***    ::*. * *:: *:*** *:*

E.coli          TFVRGIDLRS--------TVLLNVLTLPRLQKQFPNESHTENQPTWIKPIKSNESIPA--    223
Typhimurium     TLVELQEYQGERQTPLWRKLWLNVMPQDTADLPLPDQC-DATVFPWLAATRTSEQANAVT    237
Typhi           TLVVPQ---EEDKYPLWKKLWLNVLPQEEPP---NVTQ-HPLIFPWLAPTKTSEKAGNVV    228
                *:*               .: ***:                    *:   ::.*.

E.coli          --SSIGFVRGLFWQPAHIELC-DPIGIGKCSCCGQESNLRYTGFLKEKFTFTVNGLWPHP    280
Typhimurium     TPEQVNKLQAYWGMPRRIRLDFATLQSGCCDICGAESDELL-GFMTVKNYGVNYDGWRHP    296
Typhi           TPDNAHPLQAYWGMPRRIELDFTHTVAGICDLCGEHHESLL-LQMRSKNYGVQYDSWLHP    287
                  ..   ::. :  * :*.*       * *. ** . :      :  *   .  . * **

E.coli          HSPCLVTVKKGEVEEKFLAFTTS--AP---SWTQISRVVVDKIIQNENGNRVAAVVNQFR    335
Typhimurium     LTPYRAPVKDQN---AFFSVKPQPGGLIWRDWLGLSQNNQTEAN----YESPAQVVKVFN    349
Typhi           FSPYRQALKDPSA--PWLAFKGQPGGLSYKDWLGLMLNREDKFN----KMQPAKVVRAAG    341
                 :*    :*. .    :::.. .  .    .*  :      :          * **.

E.coli          NIAPQSPLELIMG--GY--RNNQAS---ILERRHDVLMFNQGWQQYGNVINEIVTVGLGY    388
Typhimurium     AR---SLTDVKAGIWGFGADFDNMKIRCWYEHHFPLLMTE----GLIPDLRKAVQTAARL    402
Typhi           QR-------NKMSLWCFAWDMNKAKVRCWYQHRIPLISVSHE-EQFLAALNIVLVLASES    393
                            .   :    :: .     :::  ::  .         :.  :  .

E.coli          ----KTALRKALYTFAEGFKNKDFKGAGVSVHETAERHFYRQSELLIPDVLANVNFSQAD    444
Typhimurium     LSLLRSALKEAWFADAKGAR-GDFSFIDIDFWNLTQGRFLNLIHDLE-------NGHKPD    454
Typhi           LSLLRNALKSAKFDCPKEAK-MDFSMVDIAFWQETEPAFRTLQEALAVDPLR--QDTQTR    450
                    :.**:.* :   :  :  **.  .: . : ::  *    . *        :  :

E.coli          EVIADLRDKLHQLCEMLFNQSVAPYAHHPKLI-STLALARATLYKHL-RELK-------P    495
Typhimurium     ERLNKWQRELWLFTRHYFDDHVFTNPYESSDLE-RIMTARKKYFTTSAEKQSAKAAKAKK    513
Typhi           HAVSQWEAELAHYLFHVFDRDALTNPDCPDDILQRQLTARQDLAS-SYRKHKARKDVLAL    509
                . : . . :*       *:  .       . :      **    .   .: .

E.coli          QGGPSNG    502
Typhimurium     QEAAE--    518
Typhi           VE-----    511
```

Figure S10

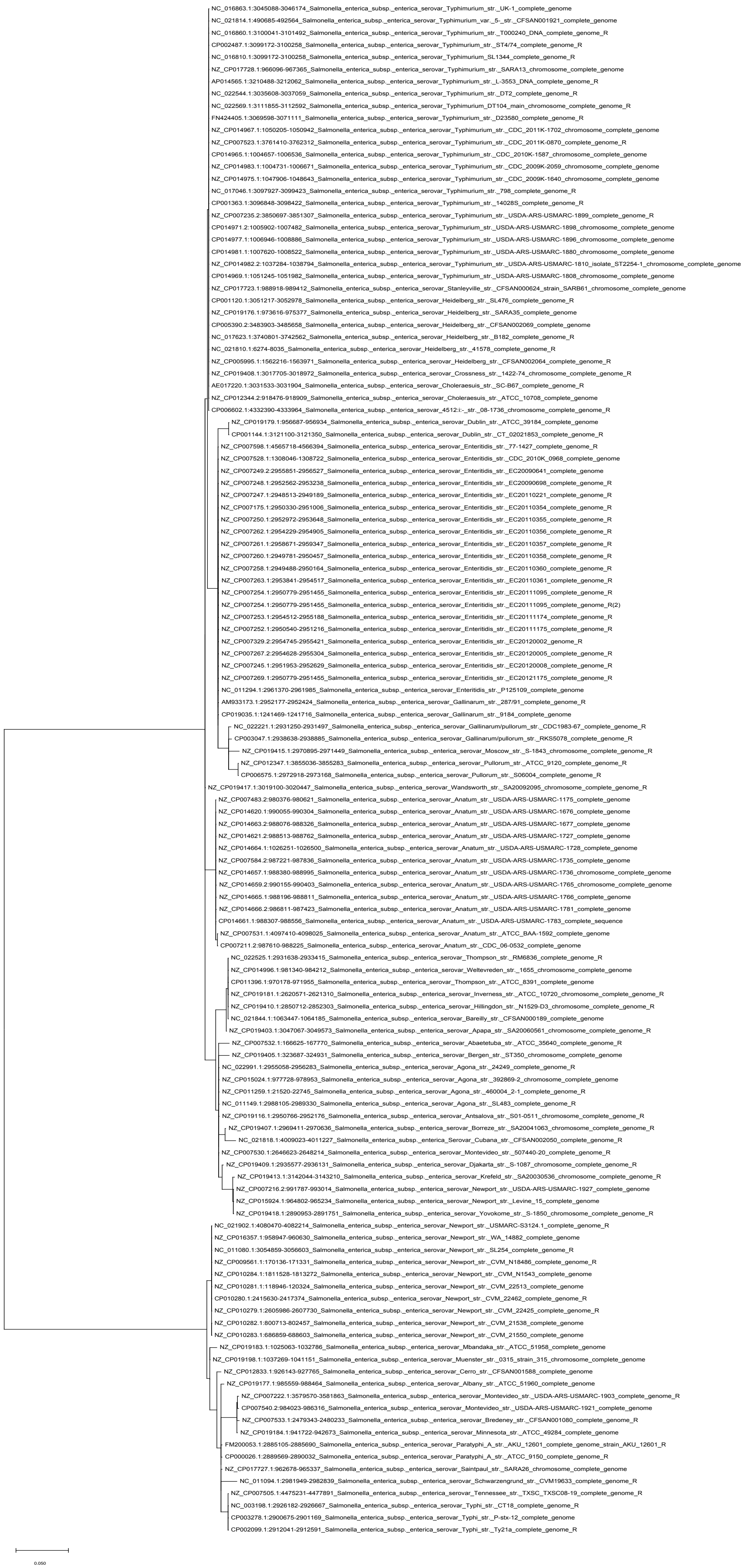
NC_016863.1:3045088-3046174_Salmonella_enterica_subsp._enterica_serovar_Typhimurium_str._UK-1_complete_genome
NC_021814.1:490685-492564_Salmonella_enterica_subsp._enterica_serovar_Typhimurium_var._5-_str._CFSAN001921_complete_genome
NC_016860.1:3100041-3101492_Salmonella_enterica_subsp._enterica_serovar_Typhimurium_str._T000240_DNA_complete_genome_R
CP002487.1:3099172-3100258_Salmonella_enterica_subsp._enterica_serovar_Typhimurium_str._ST4/74_complete_genome_R
NC_016810.1:3099172-3100258_Salmonella_enterica_subsp._enterica_serovar_Typhimurium_SL1344_complete_genome_R
NZ_CP017728.1:966096-967365_Salmonella_enterica_subsp._enterica_serovar_Typhimurium_str._SARA13_chromosome_complete_genome
AP014565.1:3210488-3212062_Salmonella_enterica_subsp._enterica_serovar_Typhimurium_str._L-3553_DNA_complete_genome_R
NC_022544.1:3035608-3037059_Salmonella_enterica_subsp._enterica_serovar_Typhimurium_str._DT2_complete_genome_R
NC_022569.1:3111855-3112592_Salmonella_enterica_subsp._enterica_serovar_Typhimurium_DT104_main_chromosome_complete_genome_R
FN424405.1:3069598-3071111_Salmonella_enterica_subsp._enterica_serovar_Typhimurium_str._D23580_complete_genome_R
NZ_CP014967.1:1050205-1050942_Salmonella_enterica_subsp._enterica_serovar_Typhimurium_str._CDC_2011K-1702_chromosome_complete_genome
NZ_CP007523.1:3761410-3762312_Salmonella_enterica_subsp._enterica_serovar_Typhimurium_str._CDC_2011K-0870_complete_genome_R
CP014965.1:1004657-1006536_Salmonella_enterica_subsp._enterica_serovar_Typhimurium_str._CDC_2010K-1587_chromosome_complete_genome
NZ_CP014983.1:1004731-1006671_Salmonella_enterica_subsp._enterica_serovar_Typhimurium_str._CDC_2009K-2059_chromosome_complete_genome
NZ_CP014975.1:1047906-1048643_Salmonella_enterica_subsp._enterica_serovar_Typhimurium_str._CDC_2009K-1640_chromosome_complete_genome
NC_017046.1:3097927-3099423_Salmonella_enterica_subsp._enterica_serovar_Typhimurium_str._798_complete_genome_R
CP001363.1:3096848-3098422_Salmonella_enterica_subsp._enterica_serovar_Typhimurium_str._14028S_complete_genome_R
NZ_CP007235.2:3850697-3851307_Salmonella_enterica_subsp._enterica_serovar_Typhimurium_str._USDA-ARS-USMARC-1899_complete_genome_R
CP014971.2:1005902-1007482_Salmonella_enterica_subsp._enterica_serovar_Typhimurium_str._USDA-ARS-USMARC-1898_chromosome_complete_genome
CP014977.1:1006946-1008886_Salmonella_enterica_subsp._enterica_serovar_Typhimurium_str._USDA-ARS-USMARC-1896_chromosome_complete_genome
CP014981.1:1007620-1008522_Salmonella_enterica_subsp._enterica_serovar_Typhimurium_str._USDA-ARS-USMARC-1880_chromosome_complete_genome
NZ_CP014982.2:1037284-1038794_Salmonella_enterica_subsp._enterica_serovar_Typhimurium_str._USDA-ARS-USMARC-1810_isolate_ST2254-1_chromosome_complete_genome
CP014969.1:1051245-1051982_Salmonella_enterica_subsp._enterica_serovar_Typhimurium_str._USDA-ARS-USMARC-1808_chromosome_complete_genome
NZ_CP017723.1:988918-989412_Salmonella_enterica_subsp._enterica_serovar_Stanleyville_str._CFSAN000624_strain_SARB61_chromosome_complete_genome
CP001120.1:3051217-3052978_Salmonella_enterica_subsp._enterica_serovar_Heidelberg_str._SL476_complete_genome_R
NZ_CP019176.1:973616-975377_Salmonella_enterica_subsp._enterica_serovar_Heidelberg_str._SARA35_complete_genome
CP005390.2:3483903-3485658_Salmonella_enterica_subsp._enterica_serovar_Heidelberg_str._CFSAN002069_complete_genome
NC_017623.1:3740801-3742562_Salmonella_enterica_subsp._enterica_serovar_Heidelberg_str._B182_complete_genome_R
NC_021810.1:6274-8035_Salmonella_enterica_subsp._enterica_serovar_Heidelberg_str._41578_complete_genome_R
NZ_CP005995.1:1562216-1563971_Salmonella_enterica_subsp._enterica_serovar_Heidelberg_str._CFSAN002064_complete_genome_R
NZ_CP019408.1:3017705-3018972_Salmonella_enterica_subsp._enterica_serovar_Crossness_str._1422-74_chromosome_complete_genome_R
AE017220.1:3031533-3031904_Salmonella_enterica_subsp._enterica_serovar_Choleraesuis_str._SC-B67_complete_genome_R
NZ_CP012344.2:918476-918909_Salmonella_enterica_subsp._enterica_serovar_Choleraesuis_str._ATCC_10708_complete_genome
CP006602.1:4332390-4333964_Salmonella_enterica_subsp._enterica_serovar_4512:i:-_str._08-1736_chromosome_complete_genome_R
NZ_CP019179.1:956687-956934_Salmonella_enterica_subsp._enterica_serovar_Dublin_str._ATCC_39184_complete_genome
CP001144.1:3121100-3121350_Salmonella_enterica_subsp._enterica_serovar_Dublin_str._CT_02021853_complete_genome_R
NZ_CP007598.1:4565718-4566394_Salmonella_enterica_subsp._enterica_serovar_Enteritidis_str._77-1427_complete_genome_R
NZ_CP007528.1:1308046-1308722_Salmonella_enterica_subsp._enterica_serovar_Enteritidis_str._CDC_2010K_0968_complete_genome
NZ_CP007249.2:2955851-2956527_Salmonella_enterica_subsp._enterica_serovar_Enteritidis_str._EC20090641_complete_genome
NZ_CP007248.1:2952562-2953238_Salmonella_enterica_subsp._enterica_serovar_Enteritidis_str._EC20090698_complete_genome_R
NZ_CP007247.1:2948513-2949189_Salmonella_enterica_subsp._enterica_serovar_Enteritidis_str._EC20110221_complete_genome_R
NZ_CP007175.1:2950330-2951006_Salmonella_enterica_subsp._enterica_serovar_Enteritidis_str._EC20110354_complete_genome_R
NZ_CP007250.1:2952972-2953648_Salmonella_enterica_subsp._enterica_serovar_Enteritidis_str._EC20110355_complete_genome_R
NZ_CP007262.1:2954229-2954905_Salmonella_enterica_subsp._enterica_serovar_Enteritidis_str._EC20110356_complete_genome_R
NZ_CP007261.1:2958671-2959347_Salmonella_enterica_subsp._enterica_serovar_Enteritidis_str._EC20110357_complete_genome_R
NZ_CP007260.1:2949781-2950457_Salmonella_enterica_subsp._enterica_serovar_Enteritidis_str._EC20110358_complete_genome_R
NZ_CP007258.1:2949488-2950164_Salmonella_enterica_subsp._enterica_serovar_Enteritidis_str._EC20110360_complete_genome_R
NZ_CP007263.1:2953841-2954517_Salmonella_enterica_subsp._enterica_serovar_Enteritidis_str._EC20110361_complete_genome_R
NZ_CP007254.1:2950779-2951455_Salmonella_enterica_subsp._enterica_serovar_Enteritidis_str._EC20111095_complete_genome_R
NZ_CP007254.1:2950779-2951455_Salmonella_enterica_subsp._enterica_serovar_Enteritidis_str._EC20111095_complete_genome_R(2)
NZ_CP007253.1:2954512-2955188_Salmonella_enterica_subsp._enterica_serovar_Enteritidis_str._EC20111174_complete_genome_R
NZ_CP007252.1:2950540-2951216_Salmonella_enterica_subsp._enterica_serovar_Enteritidis_str._EC20111175_complete_genome_R
NZ_CP007329.2:2954745-2955421_Salmonella_enterica_subsp._enterica_serovar_Enteritidis_str._EC20120002_genome_R
NZ_CP007267.2:2954628-2955304_Salmonella_enterica_subsp._enterica_serovar_Enteritidis_str._EC20120005_complete_genome_R
NZ_CP007245.1:2951953-2952629_Salmonella_enterica_subsp._enterica_serovar_Enteritidis_str._EC20120008_complete_genome_R
NZ_CP007269.1:2950779-2951455_Salmonella_enterica_subsp._enterica_serovar_Enteritidis_str._EC20121175_complete_genome_R
NC_011294.1:2961370-2961985_Salmonella_enterica_subsp._enterica_serovar_Enteritidis_str._P125109_complete_genome
AM933173.1:2952177-2952424_Salmonella_enterica_subsp._enterica_serovar_Gallinarum_str._287/91_complete_genome_R
CP019035.1:1241469-1241716_Salmonella_enterica_subsp._enterica_serovar_Gallinarum_str._9184_complete_genome
NC_022221.1:2931250-2931497_Salmonella_enterica_subsp._enterica_serovar_Gallinarum/pullorum_str._CDC1983-67_complete_genome_R
CP003047.1:2938638-2938885_Salmonella_enterica_subsp._enterica_serovar_Gallinarum/pullorum_str._RKS5078_complete_genome_R
NZ_CP019415.1:2970895-2971449_Salmonella_enterica_subsp._enterica_serovar_Moscow_str._S-1843_chromosome_complete_genome_R
NZ_CP012347.1:3855036-3855283_Salmonella_enterica_subsp._enterica_serovar_Pullorum_str._ATCC_9120_complete_genome_R
CP006575.1:2972918-2973168_Salmonella_enterica_subsp._enterica_serovar_Pullorum_str._S06004_complete_genome_R
NZ_CP019417.1:3019100-3020447_Salmonella_enterica_subsp._enterica_serovar_Wandsworth_str._SA20092095_chromosome_complete_genome_R
NZ_CP007483.2:980376-980621_Salmonella_enterica_subsp._enterica_serovar_Anatum_str._USDA-ARS-USMARC-1175_complete_genome
NZ_CP014620.1:990055-990304_Salmonella_enterica_subsp._enterica_serovar_Anatum_str._USDA-ARS-USMARC-1676_complete_genome
NZ_CP014663.2:988076-988326_Salmonella_enterica_subsp._enterica_serovar_Anatum_str._USDA-ARS-USMARC-1677_complete_genome
NZ_CP014621.2:988513-988762_Salmonella_enterica_subsp._enterica_serovar_Anatum_str._USDA-ARS-USMARC-1727_complete_genome
NZ_CP014664.1:1026251-1026500_Salmonella_enterica_subsp._enterica_serovar_Anatum_str._USDA-ARS-USMARC-1728_complete_genome
NZ_CP007584.2:987221-987836_Salmonella_enterica_subsp._enterica_serovar_Anatum_str._USDA-ARS-USMARC-1735_complete_genome
NZ_CP014657.1:988380-988995_Salmonella_enterica_subsp._enterica_serovar_Anatum_str._USDA-ARS-USMARC-1736_chromosome_complete_genome
NZ_CP014659.2:990155-990403_Salmonella_enterica_subsp._enterica_serovar_Anatum_str._USDA-ARS-USMARC-1765_chromosome_complete_genome
NZ_CP014665.1:988196-988811_Salmonella_enterica_subsp._enterica_serovar_Anatum_str._USDA-ARS-USMARC-1766_complete_genome
NZ_CP014666.2:986811-987423_Salmonella_enterica_subsp._enterica_serovar_Anatum_str._USDA-ARS-USMARC-1781_complete_genome
CP014661.1:988307-988556_Salmonella_enterica_subsp._enterica_serovar_Anatum_str._USDA-ARS-USMARC-1783_complete_sequence
NZ_CP007531.1:4097410-4098025_Salmonella_enterica_subsp._enterica_serovar_Anatum_str._ATCC_BAA-1592_complete_genome
CP007211.2:987610-988225_Salmonella_enterica_subsp._enterica_serovar_Anatum_str._CDC_06-0532_complete_genome
NC_022525.1:2931638-2933415_Salmonella_enterica_subsp._enterica_serovar_Thompson_str._RM6836_complete_genome_R
NZ_CP014996.1:981340-984212_Salmonella_enterica_subsp._enterica_serovar_Weltevreden_str._1655_chromosome_complete_genome
CP011396.1:970178-971955_Salmonella_enterica_subsp._enterica_serovar_Thompson_str._ATCC_8391_complete_genome
NZ_CP019181.1:2620571-2621310_Salmonella_enterica_subsp._enterica_serovar_Inverness_str._ATCC_10720_chromosome_complete_genome_R
NZ_CP019410.1:2850712-2852303_Salmonella_enterica_subsp._enterica_serovar_Hillingdon_str._N1529-D3_chromosome_complete_genome_R
NC_021844.1:1063447-1064185_Salmonella_enterica_subsp._enterica_serovar_Bareilly_str._CFSAN000189_complete_genome
NZ_CP019403.1:3047067-3049573_Salmonella_enterica_subsp._enterica_serovar_Apapa_str._SA20060561_chromosome_complete_genome_R
NZ_CP007532.1:166625-167770_Salmonella_enterica_subsp._enterica_serovar_Abaetetuba_str._ATCC_35640_complete_genome_R
NZ_CP019405.1:323687-324931_Salmonella_enterica_subsp._enterica_serovar_Bergen_str._ST350_chromosome_complete_genome
NC_022991.1:2955058-2956283_Salmonella_enterica_subsp._enterica_serovar_Agona_str._24249_complete_genome_R
NZ_CP015024.1:977728-978953_Salmonella_enterica_subsp._enterica_serovar_Agona_str._392869-2_chromosome_complete_genome
NZ_CP011259.1:21520-22745_Salmonella_enterica_subsp._enterica_serovar_Agona_str._460004_2-1_complete_genome_R
NC_011149.1:2988105-2989330_Salmonella_enterica_subsp._enterica_serovar_Agona_str._SL483_complete_genome_R
NZ_CP019116.1:2950766-2952176_Salmonella_enterica_subsp._enterica_serovar_Antsalova_str._S01-0511_chromosome_complete_genome_R
NZ_CP019407.1:2969411-2970636_Salmonella_enterica_subsp._enterica_serovar_Borreze_str._SA20041063_chromosome_complete_genome_R
NC_021818.1:4009023-4011227_Salmonella_enterica_subsp._enterica_Serovar_Cubana_str._CFSAN002050_complete_genome_R
NZ_CP007530.1:2646623-2648214_Salmonella_enterica_subsp._enterica_serovar_Montevideo_str._507440-20_complete_genome_R
NZ_CP019409.1:2935577-2936131_Salmonella_enterica_subsp._enterica_serovar_Djakarta_str._S-1087_chromosome_complete_genome_R
NZ_CP019413.1:3142044-3143210_Salmonella_enterica_subsp._enterica_serovar_Krefeld_str._SA20030536_chromosome_complete_genome_R
NZ_CP007216.2:991787-993014_Salmonella_enterica_subsp._enterica_serovar_Newport_str._USDA-ARS-USMARC-1927_complete_genome
NZ_CP015924.1:964802-965234_Salmonella_enterica_subsp._enterica_serovar_Newport_str._Levine_15_complete_genome
NZ_CP019418.1:2890953-2891751_Salmonella_enterica_subsp._enterica_serovar_Yovokome_str._S-1850_chromosome_complete_genome_R
NC_021902.1:4080470-4082214_Salmonella_enterica_subsp._enterica_serovar_Newport_str._USMARC-S3124.1_complete_genome_R
NZ_CP016357.1:958947-960630_Salmonella_enterica_subsp._enterica_serovar_Newport_str._WA_14882_complete_genome
NC_011080.1:3054859-3056603_Salmonella_enterica_subsp._enterica_serovar_Newport_str._SL254_complete_genome_R
NZ_CP009561.1:170136-171331_Salmonella_enterica_subsp._enterica_serovar_Newport_str._CVM_N18486_complete_genome_R
NZ_CP010284.1:1811528-1813272_Salmonella_enterica_subsp._enterica_serovar_Newport_str._CVM_N1543_complete_genome
NZ_CP010281.1:118946-120324_Salmonella_enterica_subsp._enterica_serovar_Newport_str._CVM_22513_complete_genome
CP010280.1:2415630-2417374_Salmonella_enterica_subsp._enterica_serovar_Newport_str._CVM_22462_complete_genome_R
NZ_CP010279.1:2605986-2607730_Salmonella_enterica_subsp._enterica_serovar_Newport_str._CVM_22425_complete_genome_R
NZ_CP010282.1:800713-802457_Salmonella_enterica_subsp._enterica_serovar_Newport_str._CVM_21538_complete_genome
NZ_CP010283.1:686859-688603_Salmonella_enterica_subsp._enterica_serovar_Newport_str._CVM_21550_complete_genome
NZ_CP019183.1:1025063-1032786_Salmonella_enterica_subsp._enterica_serovar_Mbandaka_str._ATCC_51958_complete_genome
NZ_CP019198.1:1037269-1041151_Salmonella_enterica_subsp._enterica_serovar_Muenster_str._0315_strain_315_chromosome_complete_genome
NZ_CP012833.1:926143-927765_Salmonella_enterica_subsp._enterica_serovar_Cerro_str._CFSAN001588_complete_genome
NZ_CP019177.1:985559-988464_Salmonella_enterica_subsp._enterica_serovar_Albany_str._ATCC_51960_complete_genome
NZ_CP007222.1:3579570-3581863_Salmonella_enterica_subsp._enterica_serovar_Montevideo_str._USDA-ARS-USMARC-1903_complete_genome_R
CP007540.2:984023-986316_Salmonella_enterica_subsp._enterica_serovar_Montevideo_str._USDA-ARS-USMARC-1921_complete_genome
NZ_CP007533.1:2479343-2480233_Salmonella_enterica_subsp._enterica_serovar_Bredeney_str._CFSAN001080_complete_genome_R
NZ_CP019184.1:941722-942673_Salmonella_enterica_subsp._enterica_serovar_Minnesota_str._ATCC_49284_complete_genome
FM200053.1:2885105-2885690_Salmonella_enterica_subsp._enterica_serovar_Paratyphi_A_str._AKU_12601_complete_genome_strain_AKU_12601_R
CP000026.1:2889569-2890032_Salmonella_enterica_subsp._enterica_serovar_Paratyphi_A_str._ATCC_9150_complete_genome_R
NZ_CP017727.1:962678-965337_Salmonella_enterica_subsp._enterica_serovar_Saintpaul_str._SARA26_chromosome_complete_genome
NC_011094.1:2981949-2982839_Salmonella_enterica_subsp._enterica_serovar_Schwarzengrund_str._CVM19633_complete_genome_R
NZ_CP007505.1:4475231-4477891_Salmonella_enterica_subsp._enterica_serovar_Tennessee_str._TXSC_TXSC08-19_complete_genome_R
NC_003198.1:2926182-2926667_Salmonella_enterica_subsp._enterica_serovar_Typhi_str._CT18_complete_genome_R
CP003278.1:2900675-2901169_Salmonella_enterica_subsp._enterica_serovar_Typhi_str._P-stx-12_complete_genome
CP002099.1:2912041-2912591_Salmonella_enterica_subsp._enterica_serovar_Typhi_str._Ty21a_complete_genome_R
0.050

Figure S11

a.

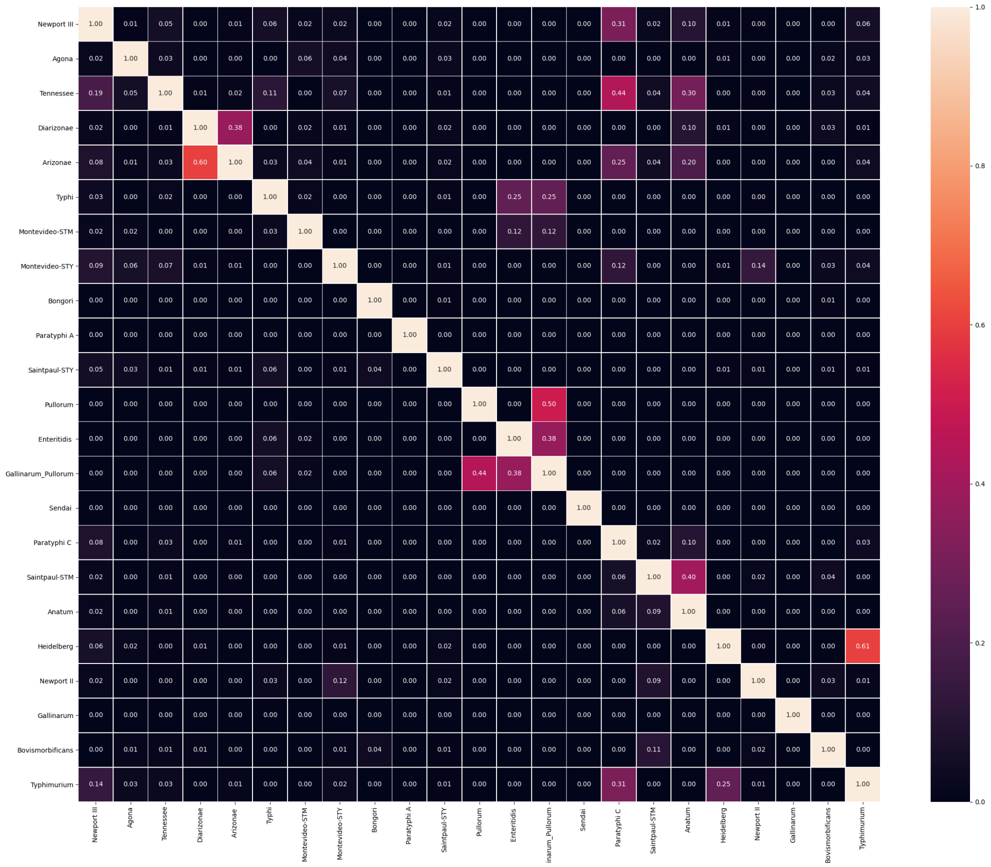

b.

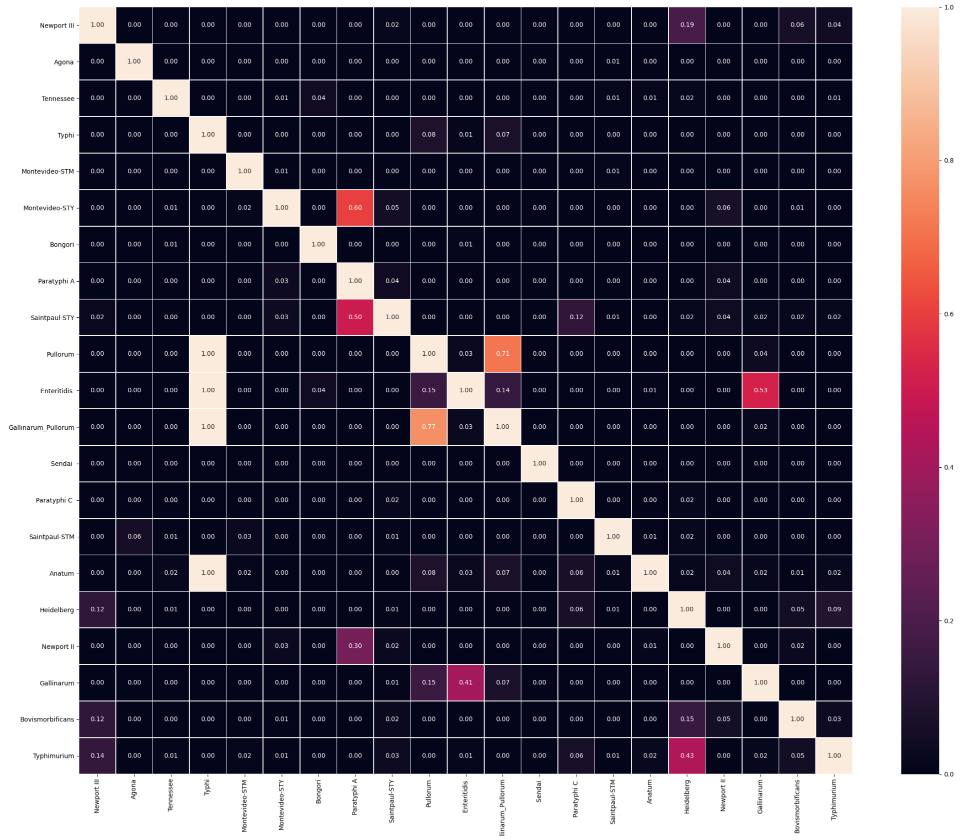

Figure S12

a.

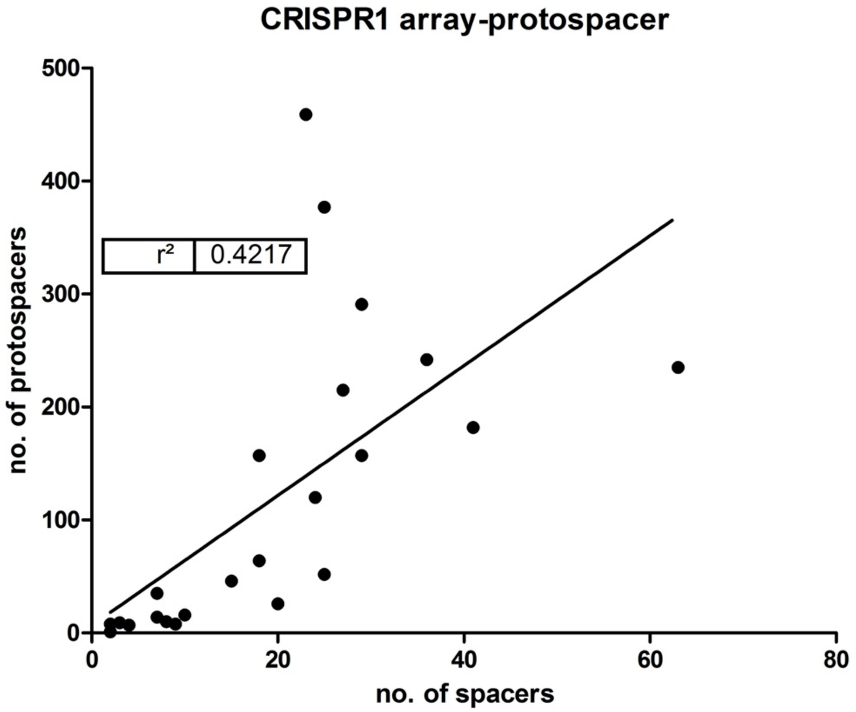

b.

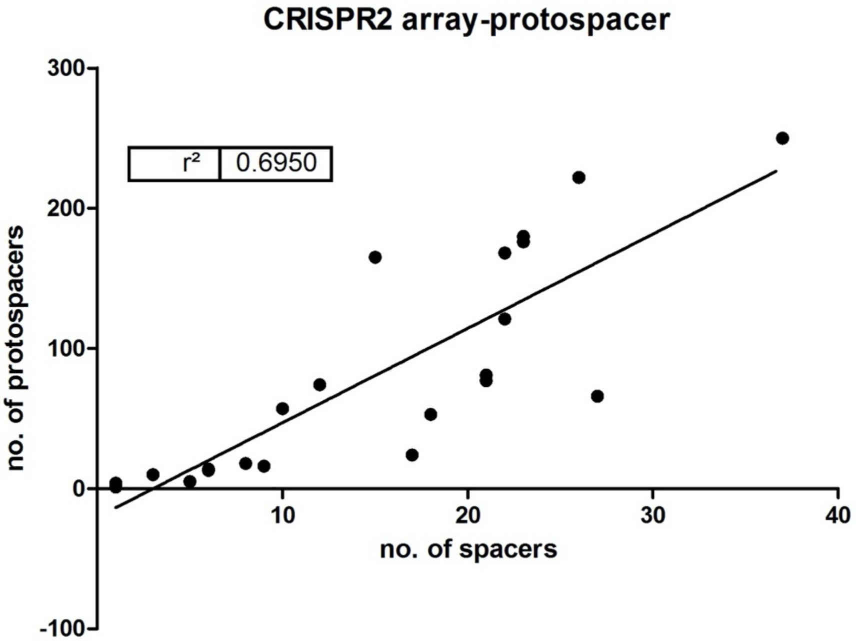

Figure S13

Description	Max Score	Total Score	Query cover	E Value	Per. Ident	Accession
CP006048.1:492564-522104 Salmonella enterica	6680	12231	51%	0.0	81.98	Query_42183

```
Alignments: >Seq1
Sequence ID: Query_42183 Length: 29541
Range 1: 853 to 8764 Score:6680 bits(3617), Expect:0.0, Identities:6533/7969(82%),  Gaps:87/7969(1%), Strand: Plus/Plus
Query  13152  GTAGCCCATCTGCTGAAATTGCTGACGAATATAATCAGCAGACAACATTTCTGCCGGAGA  13211
Sbjct  853    GTATCCCATCTGGGTAAATTGTTGGCGTAAATAATCGGCAGACAACATCTCGGCGGGGGA  912
Query  13212  GCCCGTTACGCGGCCTGGGAAAAACGTTGCGATATATCGAGCCTGGGTATTTGCGATATC  13271
Sbjct  913    GCCCGTCATTCTCCCGGGAAAAAAGGTGGCGATATGTCGTGCCTGAGTATTCGCTATTTC  972
Query  13272  TCCAGG-TTTGGAAGATGTTGCCTGTGCGGGAAGGATAAAGCACGCGCCGAGCGCCAGGA  13330
Sbjct  973    ACCAGGCCCTGG-TGATGCTGCTTGTGCAGGAAGGATAAAGCCTACGCCGAGCGCCAG-A  1030
Query  13331  TTGCAAG-AAGGGGGCGCGTTGCGGAAAACATAATGAGTCCTTAAATACCAAGCAGAAAT  13389
Sbjct  1031   ATGACGGCAAAGCGGCGCGTTGCGGAAAACATAATGAGTCCTTAAATAC--AGAACAAAA  1088
Query  13390  TTTTA-GCCGCTAGTATGAAACCGTGATCGAGGTAAAACAATTTCATTTGCTCCTAAATG  13448
Sbjct  1089   TTTTACGTGGCTAGTATGAAACCGCGATCGTGTTAATACAATTTCATTTGCTCCTAAATG  1148
Query  13449  CCCTAAATTCGGTAGCTTAAGCACTTTTTGATATTTGCTTTTCCGG-ATCGTTATTCCTT  13507
Sbjct  1149   CTGGCAATCCGGTACGTTAAGCATTTTTTGATATT-GGTTTTTGGGCCGCGTTATTCCTT  1207
[illegible]
Sbjct  8178   CATCAGAGATGAGCAGGTGCTTAATGCGCTTGCTGCAGTGCCGCGCGAGAAATTTATTGA  8237
Query  20564  TGAGGCGTTTGAACATAAGGCCTGGGACAATATTGCCCTGCCGATTGGTCAGGGGCAGAC  20623
Sbjct  8238   TGAAGCGTTTGAACATAAGGCCTGGGAAAATATCGCTTTGCCGATAGGCCAGGGTCAGAC  8297
Query  20624  GATTTCGCAGCCTTATATGGTTGCGCGAATGACCGAACTGCTTGAGCTAACGCCGCAGTC  20683
Sbjct  8298   GATTTCGCAGCCCTATATGGTGGCGCGAATGACGGAGCTGCTCGAACTGACGCCGCAATC  8357
Query  20684  TCGGGTGCTGGAAATTGGTACTGGCTCTGGCTACCAGACGGCGATTCTGGCGCACCTGGT  20743
Sbjct  8358   CAGGGTGCTGGAAATTGGTACCGGTTCCGGCTATCAGACGGCGATTCTGGCGCATCTGGT  8417
Query  20744  GCATCATGTGTGCTCCGTTGAGCGCATTAAAGGTTTGCAATGGCAGGCGCGTCGCCGCCT  20803
Sbjct  8418   ACATCACGTTTGCTCCGTTGAGCGGATTAAGGGGCTGCAATGGCAGGCGCGTCGCCGCCT  8477
Query  20804  GAAGCAGCTTGATTTACATAATGTTTCGACCCGTCATGGTGATGGATGGCAAGGCTGGCA  20863
Sbjct  8478   GAAGCAGCTCGATTTACATAATGTTTCTACCCGTCATGGCGATGGCTGGCAAGGCTGGCA  8537
Query  20864  GGCGCGAGCCCCGTTTGACGCTATCATTGTAACGGCAGCGCCGCCCGAGATCCCAACCGC  20923
Sbjct  8538   GGCGCGTGCGCCATTTGACGCTATCATTGTGACGGCCGCGCCGCCGGAAATTCCTACCGC  8597
Query  20924  ATTGATGATGCA-AC-TGGATGAGGGCGGCATTCTCGTCTTACCCGTGGGCGATGAGCAC  20981
Sbjct  8598   GCTCATG--GCACAGTTGGATGAAGGCGGCATTCTTGTTCTGCCCGTGGGCGATGAGCAG  8655
Query  20982  CAGTTTCTGAAGCGGGTGCGTCGTCGGGGAGGCGAATTTATTATCGATACCGTGGAGGCC  21041
Sbjct  8656   CAGTTTTTGAAACGCGTGCGTCGCCGGGGCGGCGAATTTATTATCGATACCGTGGAGGCC  8715
Query  21042  GTACGTTTCGTTCCCTTAGTGAAAGGGGAGCTTGCGTAGATAGCGCTGC  21090
Sbjct  8716   GTTCGCTTCGTCCCGTTAGTCAAGGGAGAGCTGGCCTAGTTTGCCCTGC  8764
Range 2: 23767 to 26869 Score:2833 bits(1534), Expect:0.0, Identities:2584/3107(83%),  Gaps:7/3107(0%), Strand: Plus/Minus
Query  7215   TTCCCTTTTAATCTTGTTGGGATTTCTCTTTGTTGCAATCATTGTCTGAACCAACTGCCG  7274
Sbjct  26869  TTCCCTCTTAATTTTGTTGAAATTTCTCTTTGTTGCGAGCATGGTCTGAACTAATTGCCT  26810
[illegible]
Range 3: 8874 to 10655 Score:2052 bits(1111), Expect:0.0, Identities:1561/1783(88%),  Gaps:12/1783(0%), Strand: Plus/Plus
Query  21146  GCCTTGCACGGTGATTACGTCACTGGTT-TTAACC-AA--TTTTCCTGGGGGATAAATGA  21201
Sbjct  8874   GCGTTGCACGGTTCTTACGTCACTGGTTGTTAACCAAATTTTTTCCTGGGGGATAAATGA  8933
Query  21202  GCGCGGGAAGCCACAAATTTACCGTTCGCCGCATTGCGGCATTGTCACTGGTTTCACTAT  21261
Sbjct  8934   GCGCGGGAAGCCCAAAATTCACCGTTAGTCGCATTGCGGCACTATCACTGGTTTCACTAT  8993
Query  21262  GGCTGGCAGGTTGTTCAAACACCTCGAATCCACCAGCGCCGGTCAGCTCGGTTAACGGTA  21321
Sbjct  8994   GGCTGGCAGGTTGTACCAGTTCGTCAAACCCTCCGGCGCCGGTCACGTCTGTGGACAGTG  9053
Query  21322  GTGCATCAACGAACACCAGTTCTGGCATGCTGATTACGCCTCCACCCAAAATGGGCTCGA  21381
Sbjct  9054   GTTCGTCATCAAATACCAATTCCGGGATGTTGATCACACCGCCGCCAAAAATGGGCGCGA  9113
Query  21382  CGTCG--AC--A-G-TACAGCAGACGCCACAGATCCAGTCGGTGCAGCAACCAACCACAC  21435
Sbjct  9114   CGACGCAACAAACGCCGCAACAAGCGCCGCAAATTCAACCCGTTCAGCGTCCTGTAACGC  9173
Query  21436  AGCCAACGCAAATCCAGCCTGTTGCTCAACAGCCTGTGCAGATGGAAAACGGTCGTATTG  21495
Sbjct  9174   AGCCCATGCAAACTCAACCGGTCACGGAACAACCCGTGCAGATGGAAAATGGGCGAATCG  9233
Query  21496  TATACAATCGTAAGTATGGGAATATTCCGAAAGGTAGCTACACGGGCGGCAGTACCTATA  21555
Sbjct  9234   TATACAATCGCCAGTATGGGAATATTCCGAAAGGTAGCTATACGGGTGGCAGTACTTACA  9293
Query  21556  CTGTTAAGAAAGGCGATACCCTTTTCTATATTGCCTGGATAACCGGGAACGATTTCCGTG  21615
Sbjct  9294   CCGTGAAAAAGGGTGATACGCTTTTTACATCGCCTGGATTACCGGGAACGATTTCCGCG  9353
Query  21616  ACCTCGCACAACGCAATAACGTTCCGGCCCCGTATGGCCTGAATGTCGGTCAAACTCTGC  21675
Sbjct  9354   ATCTGGCCCAGAGAAACAGTATTTCTGCCCCGTATAGCCTGAATGTAGGGCAAACTTTGC  9413
Query  21676  AAGTGGGCAACGCGTCTGGTGCACCGATTACCGGTGGCAATGCCATCACCCAGGCGGATG  21735
Sbjct  9414   AAGTTGGTAACGCATCCGGTACGCCAATTACTGGCGGAAATGCGATCACCCAGGCGGATG  9473
Query  21736  CAGCACAGCAAGGAGTTGTGACCAACCCTGCACAAAATTCCACCGTTGCTGTTGCTTCGA  21795
Sbjct  9474   CAGCACAGCAAGGAGTTGTGACCAGGTCTGCACAAAATTCCACCGTTGCAGTTGCGTCTC  9533
Query  21796  AACCAACAATTACGTATTCTGAGGATTCAGGTGAACAAAGTGCTAACAAAATGTTGCCGA  21855
Sbjct  9534   AACCAACAATTACGTATTCTGAGGGCTCAGGTGAACAAAGTGCTAACAAAATGTTGCCAA  9593
Query  21856  ACAACAAGCCTGCTGGGACTGTGGTCACAGCGCCTGTAACGGCTCCAGCAGTTAGCACAA  21915
Sbjct  9594   ACAACAAGCCTGCTGGGACGGTTGTCACAGCGCCTGTAACGGCACCAACGGTAAGCACGA  9653
Query  21916  CCGAATCAACTGCAAGCTCAACGTCGACTAGCTCGCCAATCTCTACCTGGCGCTGGCCGA  21975
Sbjct  9654   CCGAACCAAATGCAAGCAGTACGTCAACCAGCGCGCCGATTTCCGCATGGCGCTGGCCGA  9713
Query  21976  CTGACGGCAAAGTGATCGAAAACTTTGCGGCTACCGAAGGTGGCAATAAAGGGATCGACA  22035
Sbjct  9714   CGGATGGCAAAGTGATCGAAAACTTTGGCGCTTCCGAAGGGGGCAATAAAGGGATCGACA  9773
Query  22036  TTGCAGGCAGCAAAGGACAGGCTATTGTCGCGACCGCAGATGGACGCGTCGTCTATGCCG  22095
Sbjct  9774   TTGCAGGCAGTAAGGGACAGGCTATCGTCGCAACCGCTGATGGGCGCGTCGTATATGCCG  9833
Query  22096  GTAATGCGCTGCGTGGTTACGGTAATCTTATTATCATCAAACATAACGATGATTACCTGA  22155
Sbjct  9834   GTAACGCACTGCGTGGTTACGGTAATCTTATTATCATCAAACATAACGATGATTACCTGA  9893
Query  22156  GCGCCTACGCCCATAACGATACAATGCTGGTCCGGGAACAACAAGAAATCAAGGCGGGGC  22215
[illegible]
Query  22875  GTACGATCCGACTGCCGATTCACATTGTAAAAGAGCTGAACGT  22917
Sbjct  10613  GTACGATTCGCTTGCCGATTCACATTGTTAAAGAGCTGAACGT  10655
Range 4: 10654 to 11165 Score:664 bits(359), Expect:0.0, Identities:466/518(90%),  Gaps:6/518(1%), Strand: Plus/Minus
Query  10346  TAAAGAAAAAGGCCAGTCTCAATGAGACTGGCCTTTCTTGATAGAACTTGTGCTTACTCG  10405
Sbjct  11165  TAAA-AAAAAGGCCAG--TCGA-CAGACTGGCCTTTTTTTG-ACAA-GGGTACTTACTCG  11112
Query  10406  CGGAAAAGCGCTTCGATATTCAGTCCCTGAGTCTGCAGAATTTCACGCAGACGACGCAGA  10465
Sbjct  11111  CGGAACAGCGCTTCGATATTCAGCCCCTGCGTCTGCAGAATTTCGCGCAGACGGCGCAGG  11052
Query  10466  CCTTCAACCTGAATCTGGCGAACACGTTCACGAGTGAGACCAATTTCACGGCCCACATCT  10525
Sbjct  11051  CCTTCAACCTGAATCTGACGAACACGTTCACGCGTAAGACCGATTTCACGGCCTACATCT  10992
Query  10526  TCCAGTGTCGCAGCTTCGTACCCCAGCAGACCAAAACGACGTGCCAGCACTTCACGCTGC  10585
Sbjct  10991  TCCAGTGTCGCAGCTTCATATCCCAGCAGACCGAAACGGCGCGCCAGCACTTCACGCTGT  10932
Query  10586  TTGGCGTTCAGTTCGAACAGCCATTTGACGATGCTCTGTTTCATATCATCGTCTTGCGTG  10645
Sbjct  10931  TTGGCGTTCAGTTCGAACAACCATTTGACGATGCTCTGTTTCATATCGTCATCTTGCGTG  10872
Query  10646  GTGTCTTCCGGACCGTTTTCCTTTTCATCGGCCAGAATATCCAGCAACGCTTTTTCGGAA  10705
Sbjct  10871  GTGTCTTCCGGACCGTTCTCTTTTTCATCGGCCAGGATGTCCAGCAACGCTTTTTCGGAA  10812
Query  10706  TCGCCACCCAGCGGGGTGTCTACCGAGGTAATACGCTCGTTGAGTCGGAGCATACGGCTG  10765
Sbjct  10811  TCACCGCCCAGCGGGGTGTCTACCGAGGTAATGCGCTCGTTGAGACGAAGCATACGGCTG  10752
Query  10766  ACGTCATCAACCGGTTTATCCAGTTGTTCAGCAATCTCTTCTGCACTCGGTTCGTGGTCC  10825
Sbjct  10751  ACGTCATCAACCGGTTTATCCAGTTGCTCTGCAATTTCTTCCGCACTCGGTTCGTGGTCC  10692
Query  10826  AGTTTATGCGACAACTCACGCGCGGTACGCAGATAGAC  10863
Sbjct  10691  AGTTTATGCGACAACTCACGTGCGGTGCGCAGGTATAC  1065
```

Supplementary Methodology

CRISPR loci data collection in correct orientation:

Our study comprises 133 strains belonging to two species, *S. bongori,* and *S. enterica,* including 22 serovars and three subspecies (supplementary table S1). These samples were primitively isolated from multiple sources, including primates, poultry, swine, cattle, food specimens, and natural environment (GenBank database). The complete genome sequences for all these annotated strains were obtained from the GenBank database. Only experimentally validated sequences were considered to ensure the legitimacy of the data being used. The CRISPR loci were identified in two steps - the annotation and orientation of the CRISPR array were retrieved from the online database of CRISPR- Cas++[19]. The upstream and downstream regions of these arrays were aligned with the leader sequences previously reported by[19] to know the correct sequence of the CRISPR array. The arrays were then classified as CRISPR1 and CRISPR2 after verifying the leader sequence and its position with respect to the *cas* operon[20].

Most strains of *S. enterica* subsp. *enterica* had both, the CRISPR1 and CRISPR2 arrays. However, all the analyzed strains of *S. enterica* subsp. *enterica* serovar Heidelberg, a few strains of serovar Typhimurium, and one strain of serovar Tennessee are reported to harbor more than two CRISPR arrays[19]. Instead, our analysis confirmed that the CRISPR1 array of serovars Typhimurium and Heidelberg were divided into two parts by a stretch of 74 nucleotides consisting of two truncated spacers and a direct repeat (DR) (supplementary fig. S1A). The two parts of the CRISPR1 array taken together in concatenation aligned well with the intact CRISPR1 array of other strains of serovar Typhimurium. Similarly, the CRISPR1 array of serovar Tennessee strain (str.) CFSAN070645 was divided into three parts (containing 19, 24, and 16 spacers) and the CRISPR2 into two parts (consisting 10 and 11 spacers) due to the presence of mutated DRs rendering a stretch of 91bp undetectable as a part of the CRISPR array. Therefore, we considered the concatenated forms of these CRISPR arrays as a single unit for further analysis. Our analysis also indicated the occurrence of CRISPR1 array with two spacers each, in the serovars Dublin, Gallinarum, Pullorum, and Gallinarum/Pullorum. However, neither of these CRISPR arrays were described as valid in the CRISPR-Cas++ database, and the CRISPRCasFinder software allocated 27bp long DRs and 34bp long spacer sequences. Likewise, the CRISPR2 arrays of serovar Typhi and serovar Pullorum str. S06004 identified through our analysis were not detectable by this database. The CRISPR2 array of serovar Typhi possessed only one erratic spacer and that of serovar Pullorum str. S06004 had two spacers. We considered all these strains and their respective CRISPR-Cas systems in our analysis.

Protospacer analysis:

The spacer sequences were extracted from the CRISPR-Cas++ database. The data of all the strains belonging to one particular serovar was combined. A unique spacer set was created for that serovar. The spacer sequences were then uploaded on CRISPRTarget tool[20]. Genbank Phage, RefSeq-Plasmid and IMGVR databases were selected as the target databases. The parameters for initial BLAST screen in CRISPRTarget were kept default. Output obtained gave the accession number of the protospacer sources corresponding to these spacers. The hits obtained for Genbank Phage and RefSeq-Plasmid had accession numbers corresponding to NCBI. While, the accession number for the hits obtained from IMGVR database corresponds to IMG/VR viral resource. The accession numbers of the protospacer hits obtained were matched across serovars using a customized bash script. Based on these matches a heat map was created.

Mapping 236 spacer- and Cascade-binding sites obtained by ChIP seq of Cas5:

The ChIP analysis done by Stringer *et al.*[21] revealed 236 binding sites. We mapped these sites on the complete genome (CP001363) available at NCBI and extracted the genes present at these sites. The functions of these gene were checked using UniProt. The genes having role in virulence (with support from literature) are marked in bold in Table S8.

References:

1. Bogomolnaya, L.M., Tilvawala, R., Elfenbein, J.R., Cirillo, J.D. & Andrews-Polymenis, H.L. Linearized Siderophore Products Secreted via MacAB Efflux Pump Protect Salmonella enterica Serovar Typhimurium from Oxidative Stress. *mBio.* **11**(2020).
2. Bearson, B.L. *et al.* Iron regulated genes of Salmonella enterica serovar Typhimurium in response to norepinephrine and the requirement of fepDGC for norepinephrine-enhanced growth. *Microbes. Infect.* **10**, 807-16 (2008).
3. Hensel, M., Hinsley, A.P., Nikolaus, T., Sawers, G. & Berks, B.C. The genetic basis of tetrathionate respiration in Salmonella typhimurium. *Mol. Microbiol.* **32**, 275-87 (1999).
4. Rodríguez-Morales, O. *et al.* Salmonella enterica serovar Typhimurium ompS1 and ompS2 mutants are attenuated for virulence in mice. *Infect. Immun.* **74**, 1398-402 (2006).
5. Coombes, B.K., Brown, N.F., Kujat-Choy, S., Vallance, B.A. & Finlay, B.B. SseA is required for translocation of Salmonella pathogenicity island-2 effectors into host cells. *Microbes. Infect.* **5**, 561-70 (2003).
6. Mühlig, A., Kabisch, J., Pichner, R., Scherer, S. & Müller-Herbst, S. Contribution of the NO-detoxifying enzymes HmpA, NorV and NrfA to nitrosative stress protection of Salmonella Typhimurium in raw sausages. *Food Microbiol.* **42**, 26-33 (2014).
7. Mariscotti, J.F. & García-del Portillo, F. Genome expression analyses revealing the modulation of the Salmonella Rcs regulon by the attenuator IgaA. *J. Bacteriol.* **191**, 1855-67 (2009).
8. Crouch, M.L., Castor, M., Karlinsey, J.E., Kalhorn, T. & Fang, F.C. Biosynthesis and IroC-dependent export of the siderophore salmochelin are essential for virulence of Salmonella enterica serovar Typhimurium. *Mol. Microbiol.* **67**, 971-83 (2008).
9. Johnson, R. *et al.* The Type III Secretion System Effector SptP of Salmonella enterica Serovar Typhi. *J. Bacteriol.* **199**(2017).
10. López-Garrido, J. & Casadesús, J. Crosstalk between virulence loci: regulation of Salmonella enterica pathogenicity island 1 (SPI-1) by products of the std fimbrial operon. *PLoS One.* **7**, e30499 (2012).
11. Tsolis, R.M. *et al.* SspA is required for lethal Salmonella enterica serovar Typhimurium infections in calves but is not essential for diarrhea. *Infect. Immun.* **68**, 3158-63 (2000).
12. El Hag, M. *et al.* Contribution of the csgA and bcsA genes to Salmonella enterica serovar Pullorum biofilm formation and virulence. *Avian. Pathol.* **46**, 541-547 (2017).
13. Choi, E. *et al.* Elongation factor P controls translation of the mgtA gene encoding a Mg. *Microbiologyopen.* **8**, e00680 (2019).
14. Velayudhan, J. *et al.* Distinct roles of the Salmonella enterica serovar Typhimurium CyaY and YggX proteins in the biosynthesis and repair of iron-sulfur clusters. *Infect. Immun.* **82**, 1390-401 (2014).
15. Tamayo, R., Prouty, A.M. & Gunn, J.S. Identification and functional analysis of Salmonella enterica serovar Typhimurium PmrA-regulated genes. *F.E.M.S. Immunol. Med. Microbiol.* **43**, 249-58 (2005).
16. Ledeboer, N.A., Frye, J.G., McClelland, M. & Jones, B.D. Salmonella enterica serovar Typhimurium requires the Lpf, Pef, and Tafi fimbriae for biofilm formation on HEp-2 tissue culture cells and chicken intestinal epithelium. *Infect. Immun.* **74**, 3156-69 (2006).
17. Clayton, D.J. *et al.* Analysis of the role of 13 major fimbrial subunits in colonisation of the chicken intestines by Salmonella enterica serovar Enteritidis reveals a role for a novel locus. *B.M.C. Microbiol.* **8**, 228 (2008).
18. Waskom, M. mwaskom/seaborn. *Zendo* https://doi.org/10.5281/zenodo.592845 (2020).
19. Couvin, D. *et al.* CRISPRCasFinder, an update of CRISRFinder, includes a portable version, enhanced performance and integrates search for Cas proteins. *Nucleic Acids Res.* **46**, W246-W251 (2018).
20. Shariat, N., Timme, R.E., Pettengill, J.B., Barrangou, R. & Dudley, E.G. Characterization and evolution of *Salmonella* CRISPR-Cas systems. *Microbiology.* **161**, 374-86 (2015).
21. Biswas, A., Gagnon, J.N., Brouns, S.J., Fineran, P.C. & Brown, C.M. CRISPRTarget: bioinformatic prediction and analysis of crRNA targets. *RNA Biol.* **10**, 817-27 (2013)
22. Stringer, A.M., Baniulyte, G., Lasek-Nesselquist, E., Seed, K.D. & Wade, J.T. Transcription termination and antitermination of bacterial CRISPR arrays. *Elife.* **9**(2020)
